# Development of a Prognostic Model Based on the Identification of EMT-Related lncRNAs in Triple-Negative Breast Cancer

**DOI:** 10.1155/2021/9219961

**Published:** 2021-11-27

**Authors:** Jiani Guo, Xuesong Yi, Zhuqing Ji, Mengchu Yao, Yu Yang, Wei Song, Mingde Huang

**Affiliations:** ^1^Department of Medical Oncology, The Affiliated Huaian No. 1 People's Hospital of Nanjing Medical University, Huai'an, Jiangsu, China; ^2^Department of Medical Oncology, The Huaian Clinical College of Xuzhou Medical University, Huai'an, Jiangsu, China; ^3^Department of Breast and Thyroid Surgery, The Affiliated Huaian No. 1 People's Hospital of Nanjing Medical University, Huai'an, Jiangsu, China

## Abstract

**Background:**

Triple-negative breast cancer (TNBC) remains the most incurable subtype of breast cancer owing to high heterogeneity, aggressive nature, and lack of treatment options. It is generally acknowledged that epithelial-mesenchymal transition (EMT) is the key step in tumor metastasis.

**Methods:**

With the application of TCGA and GEO databases, we identified EMT-related lncRNAs by the Cox univariate regression analysis. Optimum risk scores were calculated and used to divide TNBC patients into high-/low-risk subgroups by the median value using the Lasso regression analysis. The Kaplan–Meier and ROC curve analyses were applied for model validation. Then, we assessed the risk model from multi-omic aspects including immune infiltration, drug sensitivity, mutability spectrum, signaling pathways, and clinical indicators. We also analyzed the expression pattern of lncRNAs involved in the model using qRT-PCR in TNBC cell lines and constructed the ceRNA network.

**Results:**

The risk model was composed of EMT-related long noncoding RNAs (lncRNAs), which seemed to be valuable in the prognostic prediction of TNBC patients. The model could act as an independent prognostic factor of TNBC and showed a robust prognostic ability in the stratification analysis. Further investigation demonstrated that the expression of lncRNAs was different between high aggressive and low aggressive TNBC cell lines, as well as TNBC patients.

**Conclusions:**

Together, our study successfully established a risk model with great accuracy and efficacy in the prognostic prediction of TNBC patients.

## 1. Introduction

Triple-negative breast cancer (TNBC) is defined as a highly aggressive subtype of breast cancer, which lacks estrogen receptor (ER), progesterone receptor (PR) expression, and no amplification of human epidermal growth factor receptor 2 (HER2) [1]. TNBC represents almost 20% of all subtypes and is more likely to be diagnosed in young females under 40 years [2, 3]. The pathological characteristics of TNBC, such as high histological grade and central necrosis, make it more likely to develop relapse and visceral metastasis than other subtypes [4, 5]. Due to the absence of molecular therapeutic targets, the standard management of TNBC remains chemotherapy and radiotherapy [6]. Unfortunately, tumor resistance arises rapidly, followed by patient relapse, or metastasizes quickly, and results in poor prognosis [7]. Even immunotherapy, i.e., immune checkpoint inhibition (ICI), which has been effectively used in several types of solid tumors, has shown little efficacy for TNBC patients [8–10]. Hence, there is an urgent need to explore novel biomarkers and potential therapeutic approaches to improve the outcome of TNBC.

It is widely acknowledged that the epithelial-mesenchymal transition (EMT) is the most important step that leads to the metastasis of malignant tumors, including TNBC [11]. EMT is the process of polar epithelia transforming into the cells capable of free movement, which enhances the invasiveness of tumor cells into peripheral circulation [12]. It is recently found that EMT is closely related to multiple signaling pathways including Notch, Hedgehog, PI3K/AKT, and Wnt/*β*-catenin pathways, revealing its key position in TNBC development and great potential in improving clinical outcome of TNBC patients [13].

On the other side, long noncoding RNAs (lncRNAs) are a class of noncoding RNAs (ncRNAs) with a length of more than 200 nucleotides [14]. Dysregulation of lncRNAs had been confirmed to be crucial in TNBC progression, including cell proliferation, apoptosis, invasion, metastasis, and regulation of drug resistance [15]. Although numerous researches have focused on developing novel lncRNA-based therapeutics, there is still a long way to apply it in clinical practice.

In this study, using multi-omic analysis, we successfully identified several EMT-related lncRNAs and constructed a novel risk score prognostic model with strong efficiency on prognostic prediction. Our aim in this investigation was to understand the potential clinical application of EMT-related lncRNAs in prognostic stratification and their potential significance as biomarkers for targeted TNBC therapy. We systematically analyzed the expression and prognosis of EMT-related lncRNAs, conducted bioinformatic analyses to discuss the molecular mechanisms, and established prognostic markers for TNBC patients. These findings could provide great hope for individual treatment and prognostic prediction in TNBC patients.

## 2. Materials and Methods

### 2.1. Datasets

**Figure 1:**
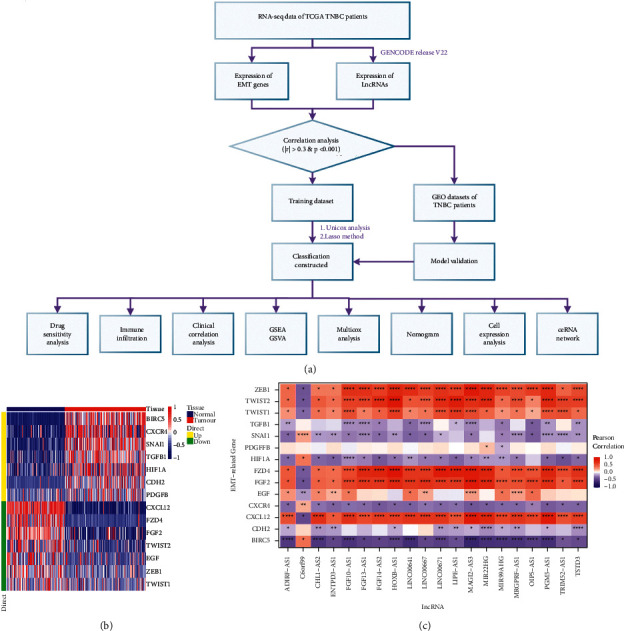
Workflow of the study and EMT-related lncRNAs in TNBC. (a) Workflow of this study. (b) Identification of 14 differential EMT genes in the expression profile between the tumor group and the normal group by differential analysis (|logFC| > 1 and *P* < 0.05), including 7 upregulated genes and 7 downregulated genes. (c) Correlation between 20 randomly selected lncRNAs and 14 EMT-related genes.

The workflow of our study is shown in Figure 1(a). The Cancer Genome Atlas (TCGA, https://portal.gdc.cancer.gov/), a landmark cancer genomic program, molecularly characterized over 20,000 primary cancer and matched normal samples spanning 33 cancer types. In this study, the lncRNA and mRNA expression profiles were extracted, respectively, from the TNBC data (processed gene expression tables from the raw files), which contained 159 TNBC and 113 non-tumor tissues, and were downloaded from TCGA database. On the other hand, the Gene Expression Omnibus (GEO, https://www.ncbi.nlm.nih.gov/geo/) represents the most extensive and comprehensive public gene expression data resources, which contain data of RNA expression, single nucleotide polymorphisms (SNPs), methylation, protein binding, and expression from almost all diseases. 83 TNBC cases with complete expression profile and survival information were extracted from the GSE135565 series matrix file, and 107 TNBC cases with complete expression profile and survival information were extracted from the GSE103091 series matrix file. Both of them were downloaded from the GEO database and annotated based on the Agilent GPL570 platform (Affymetrix Human Genome U133 Plus 2.0 Array). The EMT gene set including 22 EMT-related inducible factors, transcription factors, and signaling pathway genes was obtained from published literatures [16, 17].

### 2.2. Construction of Prognostic Model

The EMT genes with differential expression between the tumor group and the normal group were screened out (|logFC| > 1 and *P* < 0.05), and lncRNAs related to EMT genes (|*r*| > 0.3 and *P* < 0.001) were screened by correlation analysis. TNBC cases were randomly divided into training dataset and testing dataset at the ratio of 4 : 1, and then, the “glmnet” package was used on the training dataset to perform the Lasso regression analysis to further construct the prognostic model. After incorporating the expression value of each specific gene, the risk score formula of each patient was constructed and weighted with its estimated regression coefficient in the Lasso regression analysis. The EMT-lncRNA risk model formula is as follows: risk score=∑(*β*_*f*_*∗*Exp_*f*_), where *β*_*f*_ represented the Lasso coefficient of *f*^th^ gene and Exp_*f*_ represented the expression value of *f*^th^ gene. According to the formula, the training dataset patients were divided into low-risk group and high-risk group using the median risk score as the cut-off point. In addition, the TCGA testing dataset and the two GEO external testing datasets (GSE135565 and GSE103091) calculated the score of each patient through the risk score formula and grouped them according to the median value. The survival differences between the two groups were assessed by the Kaplan–Meier survival curves using log-rank tests. The Lasso regression analysis and the stratified analysis were applied for examining the role of the risk score in predicting clinical outcomes. The “survivalROC” package was used to make ROC curves to investigate the accuracy of model prediction.

### 2.3. Immunocyte Infiltration Analysis

CIBERSORT is a way to characterize cell composition from gene expression profile, as well as the most commonly cited tool for estimating and analyzing immune cell infiltration. CIBERSORT algorithm was used to analyze RNA-seq data of different TNBC subgroups to infer the relative proportion of 22 immune infiltrating cells. The sum of all estimated immune cell types in each sample was equal to 1. Spearman's correlation analysis was performed based on gene expression and immune cell content, and *P* < 0.05 was considered to be statistically significant.

### 2.4. Drug Sensitivity Analysis

Based on the GDSC database (https://www.cancerrxgene.org/), an R package “pRRophetic” was employed in the chemosensitivity prediction of each tumor sample. The IC50 of each specific chemotherapy drug was estimated by the regression method, and the GDSC training dataset was used for 10-fold cross-validation to test the regression and prediction accuracy. Default values were selected for all parameters, including “combat” to remove batch effect and the average value of repeated gene expression.

### 2.5. Mutation Spectrum

SNP-related data were downloaded from TNBC, and the mutant genes were obtained from the SNP data of TNBC sample VarScan. We selected the genes with the mutation frequency of top 30 as the display, compared the differences in the mutant genes between the two groups, and drew the mutation landscape with R package “ComplexHeatmap” to show the difference in the proportion of gene mutations between the two groups. Gene sequencing in the mutation map was based on the sum of mutation frequencies of genes in all samples.

### 2.6. Gene Set Enrichment Analysis (GSEA) and Gene Set Variation Analysis (GSVA)

The gene set enrichment analysis (GSEA, http://www.broadinstitute.org/gsea) is an effective method for genome-wide expression microarray data, which uses predefined datasets to sort the genes based on their expression in two kinds of samples, and tests their enrichment in the sorting table. In this study, GSEA on expression profiles of TNBC patients was applied for identifying differentially expressed genes between the high-risk and low-risk groups. The maximum and minimum sizes of 500 and 15 genes were used to filter the gene set. After 100 permutations, rich gene sets were obtained (*P* < 0.05, false discovery rate (FDR) < 0.25).

The gene set variation analysis (GSVA) is a nonparametric and unsupervised method used to evaluate the enrichment of transcriptome gene sets. Unlike GSEA, sample grouping is not required in GSVA. It changes the gene level into the pathway level by comprehensively scoring the gene set of interest and then judges the biological function of samples. To reduce the interference of redundant information of pathways, duplicate genes and genes that have appeared in two or more pathways were removed from each gene set. In this study, we downloaded gene sets from the Molecular Signatures Database (v7.0 version, 50 hallmark pathways) and scored each gene set comprehensively to evaluate the potential biological function changes in different samples using GSVA algorithm from “GSVA” package.

### 2.7. Risk and Independent Prognostic Analysis

The survival curves were generated by the Kaplan–Meier method and analyzed by log-rank test. The Cox proportional hazards model was used for multivariate analysis. The results of logistic regression or Cox regression were visualized by nomogram model through “rms” package, the incidence risk or proportional risk was given, and the calibration curve was generated for model verification. All statistical analyses were performed in R language (version 4.0). All statistical tests were bilateral (*P* < 0.05).

### 2.8. Cell Culture

Four TNBC cell lines (MDA-MB-231, MDA-MB-468, Hs 578T, and BT-549) and one normal breast epithelial cell line (MCF 10A) were purchased from ZQXZBIO (Shanghai, China) and identified by STR authentication. All the cell lines were maintained according to the vendor's instructions. In brief, MDA-MB-231, MDA-MB-468, and Hs 578T were maintained in Dulbecco's modified Eagle's medium (DMEM; Gibco BRL, USA) containing high glucose, with 10% fetal bovine serum (FBS; Gibco, Grand Island, NY, USA) and penicillin-streptomycin. BT-549 cell lines were cultured in RPMI 1640 medium (Gibco BRL, USA) containing high glucose, with 10% FBS and penicillin-streptomycin. MCF 10A cell lines were cultured in special medium obtained from ZQXZBIO (Shanghai, China; ZQ-1311: DMEM added with 5% horse serum, 1% penicillin-streptomycin, and 2% growth supplement). All cells were placed in 37°C, 5% CO_2_ incubator.

### 2.9. RNA Isolation and qRT-PCR

Total RNA was isolated from tissues or cultured cells with TRIzol reagent (Life Technologies, USA). One microgram of total RNA was used for the reverse transcription reaction with random primers under standard conditions using PrimeScript RT Reagent Kit with gDNA Eraser (Takara, Dalian, China). The corresponding cDNA was used for subsequent qRT-PCRs using SYBR Premix Ex Taq (Takara, Dalian, China) by the manufacturer's instructions. The expression of GAPDH was used to normalize the results. An ABI 7900 Real-Time PCR System (Applied Biosystems, Foster City, CA, USA) was used to perform the data analysis. The data calculation was based on the cycle threshold (CT) (2^−ΔΔCT^) method. The assay was run in triplicate for each sample. The primer sequences are summarized in Supplementary Table 1.

### 2.10. Construction of ceRNA Network

Using multi-database analysis, we constructed the ceRNA network based on the identified lncRNAs. Firstly, the lncRNA-mediated miRNAs were investigated through NPInter database (http://bigdata.ibp.ac.cn/npinter) [18]. A total of 165 lncRNA-miRNA interactions were predicted, including 6 lncRNAs and 142 miRNAs. Next, the obtained miRNAs were used to predict 1758 miRNA-target gene interactions, which were intersected with the 165 lncRNA-miRNA interactions to construct a ceRNA network.

### 2.11. Statistical Analysis

Survival curves were generated by the Kaplan–Meier method and compared by log-rank test. The Cox proportional hazards model was used for multivariate analysis. All statistical analyses were conducted with the R language (version 3.6.1). All statistical tests were two-tailed, if applicable, and *P* < 0.05 was considered to be significant unless specified.

## 3. Results

### 3.1. Identification of EMT-Associated lncRNAs in TNBC Cohort

Our study has downloaded the original mRNA expression data of TNBC (FRKM raw files) from TCGA database and extracted 22 EMT-related regulators. Firstly, we screened a total of 14 EMT genes in the expression profile between the tumor group and the normal group by differential expression analysis (|logFC| > 1 and *P* < 0.05), including 7 upregulated genes and 7 downregulated genes (Figure 1(b)). After that, the expression data of 3234 lncRNAs from TNBC, as well as data from EMT genes, were screened by correlation analysis to find the lncRNAs highly correlated with EMT. It revealed that a total of 1033 lncRNAs were highly associated with EMT (Supplementary Table 2). Finally, the significantly downregulated lncRNAs were screened out (the expression level was 0 in more than half of the samples, or the average expression level was less than 0.3 in the samples), and ultimately, 536 lncRNAs were used as candidate gene sets for further modeling and analysis. Among them, 20 lncRNAs and 14 EMT genes were randomly selected to show the correlation in the form of heat map (Figure 1(c)).

### 3.2. Gain of Prognostic Genes and Construction of Prognostic Model

**Figure 2:**
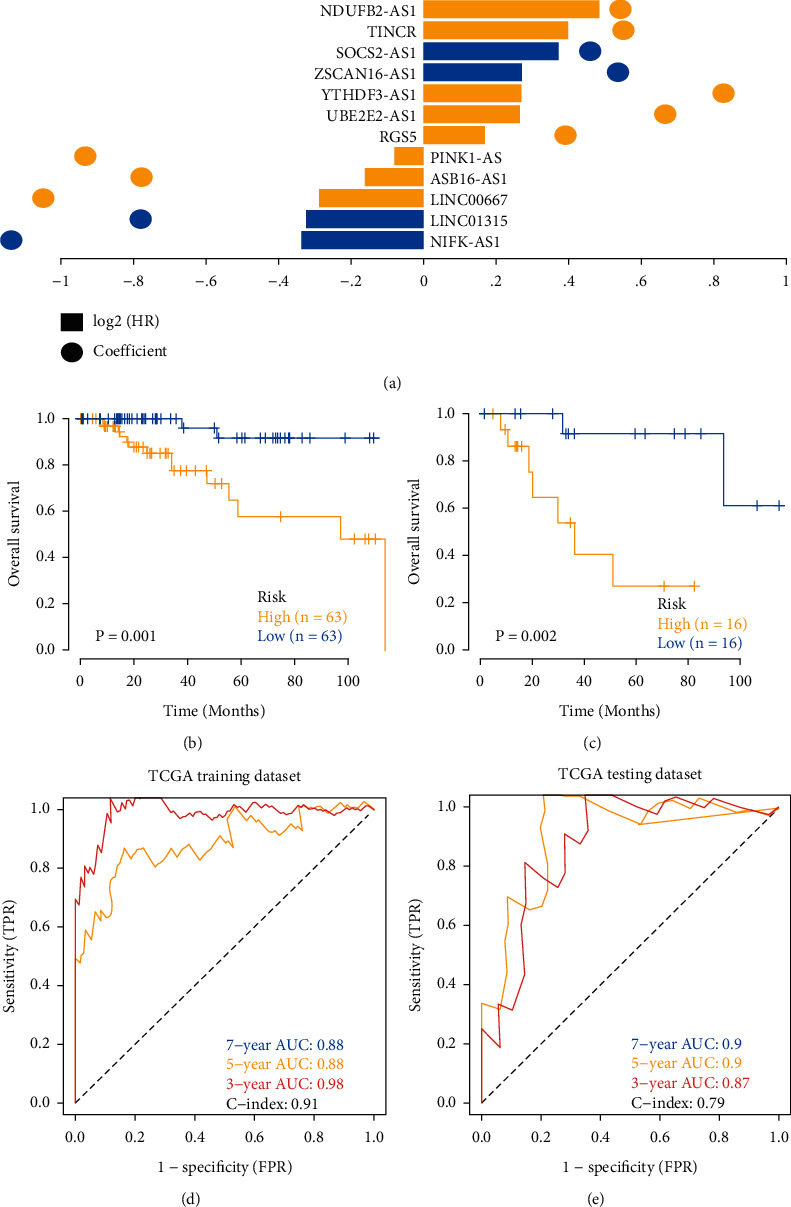
Prognostic genes used for model construction. (a) Prognostic lncRNAs were screened out from TNBC data. The overall survival (OS) of the high-risk group in both sets was significantly lower than that of the low-risk group analyzed by the Kaplan–Meier curve ((b) TCGA training dataset; (c) TCGA testing dataset). The model's efficiency is evaluated by ROC curve ((d) TCGA training dataset; (e) TCGA testing dataset).

To further identify the key genes in the screened lncRNAs set, we collected clinical information of TNBC patients and screened out the feature genes in TNBC by the Cox univariate regression and the Lasso regression feature selection algorithm (Figure 2(a), Supplementary Figure 1). It was demonstrated that 285 lncRNAs (shared genes of candidate gene set) were screened by the Cox univariate regression analysis to find the prognostic genes (Supplementary Table 3), in which 22 prognostic genes with significance (*P* value <0.05) were obtained as follows: YTHDF3-AS1, UBE2E2-AS1, SOCS2-AS1, TINCR, A2M-AS1, CYB561D2, TUG1, NIFK-AS1, LINC00667, NDUFB2-AS1, CASC15, PINK1-AS, ZSCAN16-AS1, EPB41L4A-AS1, TRIM52-AS1, LINC00839, ASB16-AS1, RGS5, LINC01023, SLC16A1-AS1, MBNL1-AS1, and LINC01315. The patients from TCGA were randomly divided into training dataset and testing dataset at a ratio of 4 : 1, and we used the Lasso regression analysis to get the best risk score value for further analysis (risk score = NIFK-AS1 × (−0.3368) + LINC01315 × (−0.3223) + LINC00667 × (−0.2887) + ASB16-AS1 × (−0.1614) + PINK1-AS × (−0.0799) + RGS5 × 0.1696 + UBE2E2-AS1 × 0.2653 + YTHDF3-AS1 × 0.2685 + ZSCAN16-AS1 × 0.2717 + SOCS2-AS1 × 0.3714 + TINCR × 0.3981 + NDUFB2-AS1 × 0.4845). According to the median of risk score, patients were divided into high-risk group and low-risk group (median value of TCGA training dataset: −0.2096; median value of TCGA testing dataset: −0.2946) and analyzed by the Kaplan–Meier curve. The overall survival (OS) of the high-risk group in both sets was significantly lower than that of the low-risk group (Figures 2(b), 2(c)). Additionally, ROC curve showed that the C-index of both sets is 0.91 and 0.79, respectively (Figures 2(d), 2(e)), indicating the model's better verification efficiency.

### 3.3. Clinical Predictive Value of the Model Based on Multi-Omic Analysis

**Figure 3:**
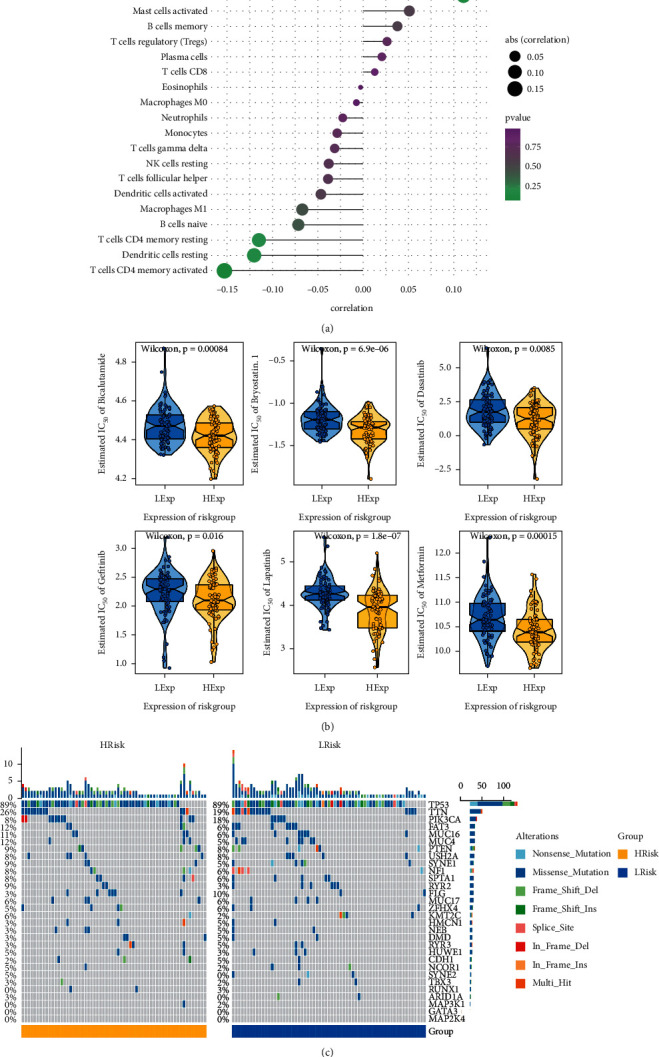
Clinical predictive value of the model. (a) Relationship between the model and tumor immune infiltration. (b) Relationship between the model and sensitivity of common chemotherapy drugs. (c) Mutation spectrum of high-/low-risk groups (left: high-risk group; right: low-risk group).

The tumor microenvironment is mainly composed of tumor-associated fibroblasts, immune cells, extracellular matrix, multiple growth factors, inflammatory factors, specific physical and chemical characteristics, and cancer cells. The tumor microenvironment significantly affects the diagnosis, survival outcome, and sensitivity of clinical treatment in cancers. Through analyzing the relationship between risk score and tumor immune infiltration, we further investigated the potential molecular mechanism of risk score in TNBC development, which demonstrated that risk score was positively correlated with macrophage M2, mast cells resting, NK cells activated, mast cells activated, etc., and negatively correlated with T-cell CD4 memory activated, dendritic cells resting, T-cell CD4 memory resting, B-cell naive, etc. (Figure 3(a)). Since surgery combined with chemotherapy is effective in early breast cancer, our research was based on the drug sensitivity data of GDSC database, and the sensitivity of each tumor sample was predicted by R package “pRRophetic” to further explore the relationship between risk score and sensitivity of common chemotherapy drugs. The results showed that risk score significantly affected the sensitivity of patients to bicalutamide, bryostatin 1, dasatinib, gefitinib, lapatinib, and metformin (Figure 3(b)). By investigating the mutation spectrum of high-/low-risk groups, we found that there was a significant difference between the two groups in the mutation proportion of multiple genes (Figure 3(c)).

### 3.4. Prognostic Model-Related Signal Mechanism

**Figure 4:**
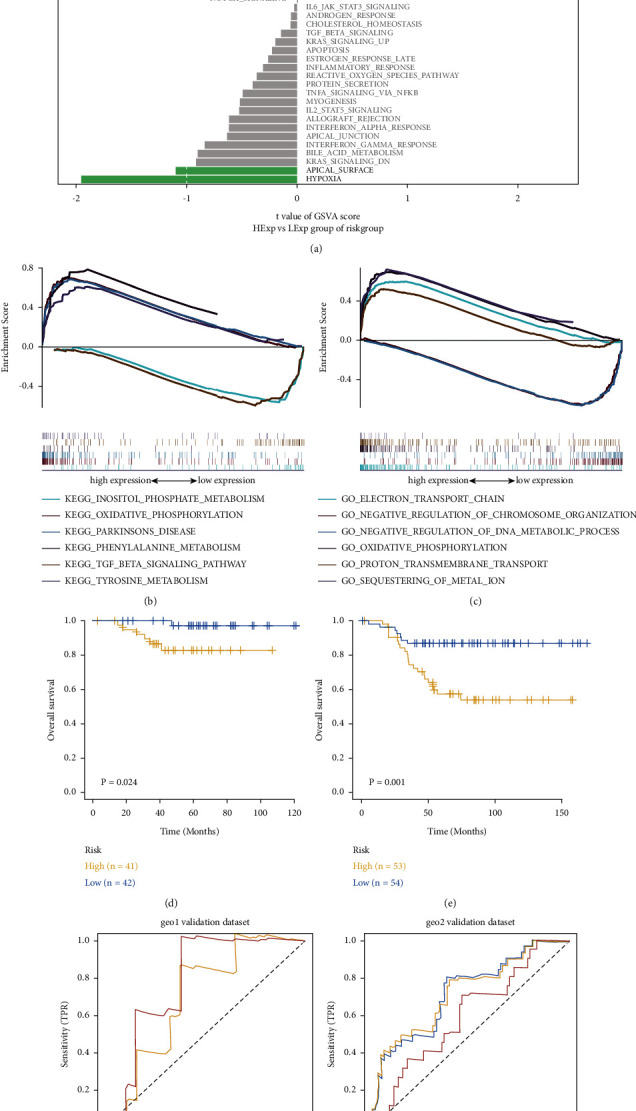
GSVA, GESA, and robustness analysis by external datasets. (a) Results of GSVA showed the differential pathways of high-/low-risk groups. (b) Results of GSEA showed the significant enrichments in various related pathways by KEGG. (c) Results of GSEA showed the significant enrichments in various related pathways by GO. (d) The survival differences between the high-/low-risk groups were evaluated by the Kaplan–Meier analysis in GSE135565. (e) The survival differences between the high-/low-risk groups were evaluated by the Kaplan–Meier analysis in GSE103091. (f) Prediction efficiency of the model verified by ROC curve in GSE135565. (g) Prediction efficiency of the model verified by ROC curve in GSE103091.

Subsequently, we analyzed the signaling pathways involved in high-/low-risk models to explore the potential molecular mechanism of risk score affecting tumor progression. Results of GSVA revealed that the differential pathways of the two groups were mainly enriched in UV response up, adipogenesis, unfolded protein response, P53 pathway, DNA repair, mitotic spindle, angiogenesis, E2F targets, G2M checkpoint, fatty acid metabolism, and hypoxia and apical surface (Figure 4(a)). Finally, we found that there were significant enrichments in various related pathways through GSEA. Some of the highly significant signaling pathways were shown (Figures 4(b), 4(c)), which suggested that the disturbance of these signaling pathways in the high-/low-risk groups affected the prognosis of TNBC. Among the enriched pathways, some of them had been clarified to play critical roles in TNBC development. For example, the P53 pathway can induce the transcription of target genes responsible for various cellular mechanisms (mainly DNA repair) and activate diverse forms of stimuli (such as hypoxia), which are consistent with our enrichment results [19]. It is widely acknowledged that the loss of P53 function may lead to deficiency in cell cycle checkpoint, genome instability, cellular immortalization, and excessive cell proliferation [20, 21]. Besides, we found the enrichment of TGF-*β* pathway, which has been recently proven to epigenetically regulate the progression of TNBC, especially through lncRNA and miRNA [22]. On the other hand, our results contained several metabolism pathways, including fatty acid metabolism and oxidative phosphorylation. According to the transcriptome analysis of metabolism dysregulation and metabolic pathway-based subtyping of TNBC, oxidative phosphorylation is reported to be the most upregulated metabolic pathway, and the MPS1 subtype is characterized by higher level of fatty acid metabolism (while the MPS2 subtype showed an upregulation of carbohydrate and nucleotide metabolism), which suggested metabolic heterogeneity, diverse prognosis, and treatment strategies between subtypes [23]. In conclusion, our enrichment results partially reflected the epigenetic and metabolic features of TNBC progression.

### 3.5. Robustness Analysis by External Datasets

We downloaded the data of TNBC patients with survival data processed in GEO databases (GSE135565 and GSE103091), predicted the clinical classification of TNBC based on the model, evaluated the survival differences between two groups through the Kaplan–Meier analysis, and investigated the stability of the prediction model. The results demonstrated that the OS of the high-risk group was obviously lower than that of the low-risk group in both GEO external verification sets (Figures 4(d), 4(e)). To verify the accuracy of the model, we did ROC curve analysis using external datasets, which showed that the model had a strong efficiency on prognostic prediction (GSE135565-C-index = 0.72, GSE103091-C-index = 0.65) (Figures 4(f), 4(g)).

### 3.6. Risk and Independent Prognostic Analysis

**Figure 5:**
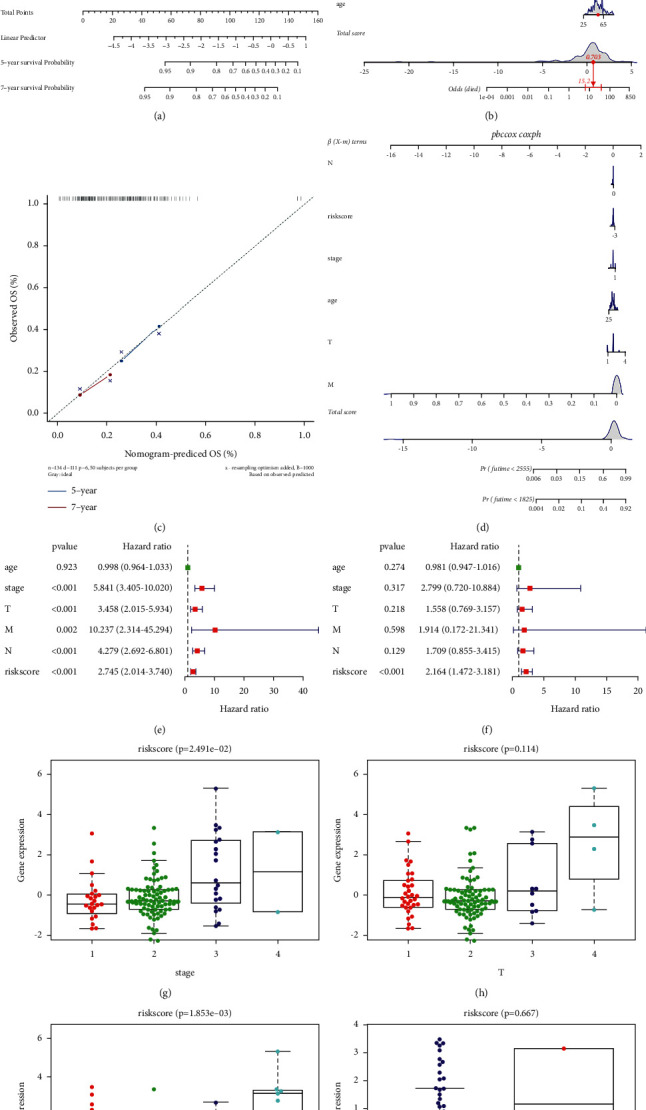
Independent prognostic analysis and correlation analysis of clinical parameters. (a) Logistic regression analysis showed the relationship between TNBC stages and distribution of risk score value. The effect of distribution of risk score and clinical parameters on TNBC stage scoring was analyzed by (b) general linear model and (d) Cox proportional hazards model. (c) Prediction analyses on the 5-year and 7-year periods. Risk score as an independent prognostic factor proved by (e) univariate and (f) bivariate analyses. Risk score values are grouped by different clinical parameters and analyzed by the Kruskal–Wallis test: (g) stage; (h) tumor; (i) lymph node; (j) metastasis.

Since the samples were divided into the high-/low-risk groups by the median value of risk score, the results of regression analysis were displayed by nomogram. The results of logistic regression analysis and generalized linear model (GLM) analysis showed that, in all our samples, risk score value has a significant contribution to the scoring process of nomogram prediction model (Figures 5(a)–5(d)). Among these, the different stages of TNBC were obviously associated with the distribution of risk score value (Figure 5(a)). We further found that the distribution of risk score and several clinical parameters (such as age, stage, and T) had different contributions to the scoring in distinct stages of cancer (Figure 5(b)). We also did some prediction analyses on the 5-year and 7-year periods (Figure 5(c)), and it was found that the prediction results were more consistent. At the same time, through univariate and bivariate analysis, it was found that our risk score was an independent prognostic factor for TNBC patients (Figures 5(e), 5(f)).

### 3.7. Correlation Analysis of Risk and Multiple Clinical Parameters

We grouped all the risk score values by different clinical parameters (tumor stage, T, N, M), which was shown in the form of boxplot graph (Figures 5(g)–5(h)), and found that these risk scores were significant among the groups with multiple clinical indicators through the Kruskal–Wallis test (*P* < 0.05) (Figures 5(g), 5(i)). As the risk score rose, the stage grade and lymph node involvement increased.

### 3.8. lncRNAs Dysregulated in TNBC and Construction of ceRNA Network

**Figure 6:**
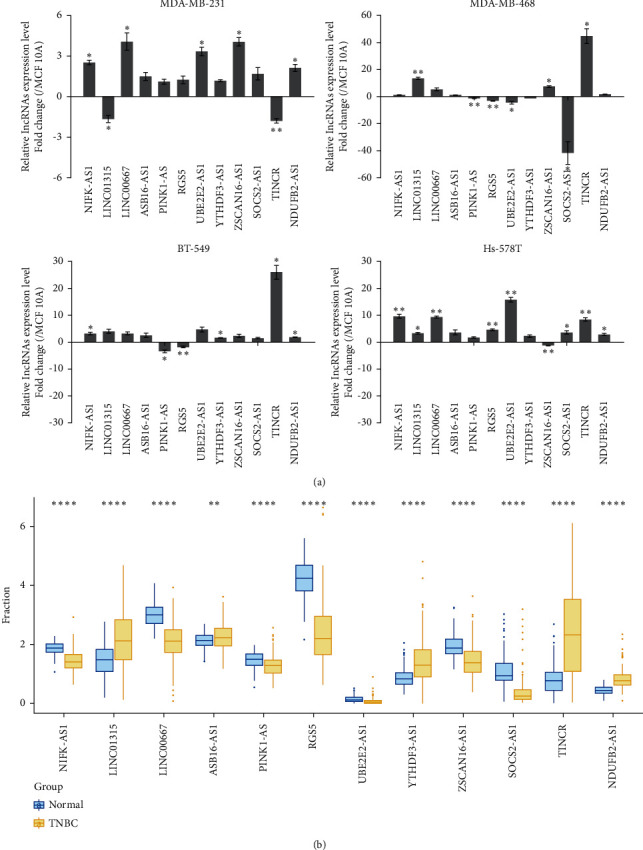
LncRNA expression in TNBC cell lines and samples. (a) Relative lncRNA expression in 4 TNBC cell lines (MDA-MB-231, MDA-MB-468, Hs 578T, and BT-549) and 1 normal breast epithelial cell line (MCF 10A). Fold change ＞0 presented the higher expression of lncRNAs in TNBC cell lines, whereas the fold change <0 meant the lower expression of lncRNAs in TNBC cell lines than in MCF 10A cells. (b) lncRNA expression in TNBC patients' tumor samples (right, yellow) and normal samples (left, blue) from TCGA. ^*∗*^*P* < 0.05, ^∗∗^*P* < 0.01, and ^∗∗∗^*P* < 0.001.

To further evaluate the expression pattern of lncRNAs involved in the risk score model, we analyzed the mRNA levels of the lncRNAs in 4 TNBC cell lines and 1 normal breast epithelial cell line, which showed the distinct expression of the lncRNAs (Figure 6(a)). Furthermore, we calculated the risk score of each cell line to verify the efficacy of the model. The results demonstrated that the risk scores based on our model were quite different between TNBC cell lines, especially in high aggressive cells (MDA-MB-231, BT-549, Hs 578T) and low aggressive cells (MDA-MB-468) [24]. The risk score of each cell line was as follows: MDA-MB-231 1.646195791, MDA-MB-468 -3.195350021, BT-549 10.36881901, and Hs 578T 3.672140084. We further analyzed the expression pattern of the lncRNAs in TNBC patients from TCGA database using the Kruskal–Wallis test, which showed the distinct expression of the lncRNAs in TNBC patients' normal and tumor tissues (Figure 6(b)). In addition, we built the ceRNA (lncRNA-miRNA-target gene) network to explore the potential mechanism of dysregulated lncRNAs in TNBC, where 6 lncRNAs were involved: TINCR, SOCS2-AS1, NDUFB2-AS1, LINC00667, PINK1-AS, and YTHDF3-AS1 (Supplementary Figure 2).

## 4. Discussion

TNBC has remained an unmet medical challenge for decades, since prone to recurrence and metastasis after operation, and no therapeutic targets have been identified [23, 25]. It is widely acknowledged that metastasis of TNBC is correlated with aberrant activation of EMT [26]. EMT is a multistep, plastic, and reversible process that allows tumor cells to acquire a mesenchymal phenotype [27]. The important characteristics of EMT include the downregulation of cell adhesion molecules (such as E-cadherin), activation of transcription factor (such as Snail2), and upregulation of mesenchymal cell markers (such as vimentin) [28]. However, the entirety accomplishment of the EMT progression demands an intricate genetic procedure, and the precise role of transcriptional and epigenetic regulators in modulating diverse EMT processes in tumorigenesis (including TNBC) is still not fully understood [25, 27, 29]. Recent studies have focused on the biological role of lncRNAs in malignant evolvement and EMT. With their multifunction, lncRNAs are proved to be related to EMT in a wide spectrum of physiological and pathological processes [30]. The promoting and suppressing effects of lncRNAs on EMT underly the complexity and plasticity of tumor cells [31]. For example, lncRNA CAR10 was reported to be an EMT promoter. CAR10 could induce EMT by directly binding with miR-30 and miR-203, and then regulating the expression of Snail1 and Slug in lung adenocarcinoma metastasis [32]. On the contrary, Han et al. [33] investigated the inhibitory effect of lncRNA CRCMSL in colorectal cancer. They pointed out that CRCMSL could bind to protein HMGB2 and stabilize the localization in the cytoplasm, hence attenuating the interaction between HMGB2 and OCT4 and inhibiting EMT. In TNBC, multiple lncRNAs had been identified to regulate EMT pathways and tumor invasion via interacting with various molecules, such as LINC01638 [34], GAS5 [35], UCA1 [36], ARNILA [37], and NNT-AS1 [38]. A better understanding of how lncRNAs regulate EMT process at diverse molecular levels can accelerate the development of therapeutic strategies and prognostic targets.

Currently, there are some applications of risk models with prognostic function in clinical. The most widely used model is the 21-gene expression assay (Oncotype DX, Genomic Health), which can provide prognostic information in hormone receptor-positive breast cancer [39]. Nevertheless, there is still a lack of simple and effective prognostic prediction model in TNBC. Researchers have begun to pay close attention to establish signatures with the combination of coding and noncoding RNAs in clinical prognosis. Recently, Lin et al. [40] constructed a hypoxia signature in the glioma groups. The hypoxia risk model could reflect the overall immune response intensity of tumor microenvironment and predict prognosis. Another research established a m6A-related lncRNA prognostic signature, which could predict the OS of lower-grade glioma patients [41]. Furthermore, Hong et al. [42] identified a novel signature. Unlike previous strategies, they paid attention to the immune-related gene pairing and built a reasonable model with two-lncRNA combinations to predict the immune landscape in hepatocellular carcinoma.

In this study, we firstly established a novel risk score prediction model based on EMT-related lncRNAs in TNBC. In combination with TCGA and GEO databases, along with 14 screened EMT factors, we performed a differential co-expression analysis to classify 536 candidate lncRNAs. 12 of them were confirmed to have prognostic value in both datasets and used to establish a model for predicting the OS of TNBC patients. According to the median value of risk score, the patients were divided into high-/low-risk groups with the significant difference in OS. Our results demonstrated that the risk score was an independent risk factor in TNBC. Since the prediction model was preliminary built, its accuracy and efficacy were carefully compared and verified from several aspects, including tumor immune infiltration, drug sensitivity, mutability spectrum, signaling pathways, and clinical parameters (age, stage, grade, clinical classification, lymph node involvement, etc.).

Among the lncRNAs involved in the model, several of them were reported to be associated with tumor progression, such as lncRNA TINCR [43–45] and TUG1 [46–48]. A recent study revealed that serum lncRNA TINCR level was significantly increased in TNBC and correlated with clinical outcome [49]. Tang et al. [50] reported that lncRNA TUG1 could act as a miR-197 sponge to enhance cisplatin sensitivity in TNBC. Additionally, LINC01315 was newly identified as a prognostic biomarker in TNBC [51]. Using qRT-PCR, we analyzed the expression pattern of the 12 lncRNAs finally involved in the risk score model, which showed that the mRNA levels between TNBC cell lines were different. In particular, the risk scores of high aggressive cells were higher than that of low aggressive cells, which further validated the effectiveness of our model. Moreover, the expression and function of several lncRNAs analyzed in previous studies are shown in Table 1. Since most of the lncRNAs were not fully investigated in TNBC, we hope that EMT-related lncRNAs might create novel insights in TNBC development.

On the other side, there existed several shortcomings and limitations. For instance, the raw data obtained from TCGA and GEO databases were incomplete and lack regional specificity, making the final model unreliable in different regions. More independent TNBC cohorts should be collected for further validation. In summary, our study demonstrated that an effective prognostic model constructed by EMT-related lncRNAs could serve as an independent risk factor and provide new strategies for TNBC patients.

## Figures and Tables

**Table 1:** lncRNAs in cancers.

lncRNA	Cancer type	Role in cancer	Molecular mechanism	Refs
NIFK-AS1	Endometrial cancer	Inhibit	Sponge miR-146a, inhibits M2-like polarization of macrophages	[53]
LINC01315	Colorectal cancer	Promote	Sponge miR-205-3p, upregulates PRKAA1	[54]
	Oral squamous cell carcinoma	Inhibit	Sponge miR-211, upregulates DLG3, activates Hippo signaling	[55]

LINC00667	Colorectal cancer	Promote	Sponge miR-449b-5p, activated by YY1	[56]
	NSCLC		Recruits EIF4A3 to stabilize VEGFA	[57]
	Cholangiocarcinoma		Sponge miR-200c-3p, promotes PDK1, activated by YY1	[58]
	Glioma		USF1/linc00667/miR-429/ALDH1A1 axis	[59]

ASB16-AS1	Gastric cancer	Promote	Sponge miR-3918 and miR-4676-3p, cooperates with ATM, induces TRIM37 phosphorylation	[60]
	HCC		Regulates miR-1827/FZD4 axis, activates wnt/*β*-catenin pathway	[61]
	Osteosarcoma		Sponge miR-760, upregulates HDGF	[62]
	Cervical cancer		miR-1305/wnt/*β*-catenin axis	[63]
	Glioma		Affects EMT signaling pathway	[64]
	Adrenocortical carcinoma	Inhibit	Promotes ubiquitination of HuR	[65]
	Clear cell renal cell carcinoma		miR-185-5p-miR-214-3p-LARP1 pathway	[66]

PINK1-AS	Gastric cancer	Promote	Sponge miR-200a, upregulates G*α*i1	[67]
ZSCAN16-AS1	HCC	Promote	Regulates miR-181c-5p/SPAG9 axis, activates the JNK pathway	[68]
SOCS2-AS1	Prostate cancer	Promote	Inhibits apoptosis pathway, promotes androgen signaling by modulating the epigenetic control for AR target genes	[69]
	Colorectal cancer	Inhibit	Sponge miR-1264, upregulates SOCS2	[70]
	Endometrial cancer		Regulates AURKA degradation	[71]

TINCR	Breast cancer	Promote	Recruits DNMT1 and increases the methylation and suppresses the transcriptional expression of miR-503-5p, sponge miR-503-5p, and upregulates EGFR, stimulates JAK2-STAT3 signaling downstream from EGFR	[45]
			Guides STAU1 to OAS1 mRNA to mediate its stability	[72]
	HCC		Interacts with TCPTP, activates STAT3 signaling	[73]
	Laryngeal squamous cell carcinoma	Inhibit	miR-210/BTG2 pathway	[74]
	Melanoma		Prevents ATF4 translation	[75]

## Data Availability

The datasets analyzed during the current study are available in the TCGA (https://portal.gdc.cancer.gov/, TNBC dataset) and GEO (https://www.ncbi.nlm.nih.gov/geo/, GSE135565 and GSE103091 datasets) repository. Data from screening are included in the additional files.

## Abbreviations

TNBC: Triple-negative breast cancer
ER: Estrogen receptor
PR: Progesterone receptor
HER2: Human epidermal growth factor receptor 2
ICI: Immune checkpoint inhibition
EMT: Epithelial-mesenchymal transition
lncRNAs: Long noncoding RNAs
ncRNAs: Noncoding RNAs
TCGA: The Cancer Genome Atlas
GEO: Gene Expression Omnibus
GSVA: Gene set variation analysis
GSEA: Gene set enrichment analysis
OS: Overall survival
GLM: General linear model
CoxPH: Cox proportional hazards.

## Additional Points

A preprint has previously been published (Jiani Guo et al. 2021) [52].

## Supplementary Materials

Supplementary Materials Supplementary Figure 1: prognostic lncRNAs were screened out from TNBC data. (A) Cox univariate regression analysis. (B) Lasso regression analysis. Supplementary Figure 2: CeRNA network. Supplementary Table 1: primers used in qRT-PCR. Supplementary Table 2: a total of 1033 lncRNAs highly associated with EMT. Supplementary Table 3: a total of 285 prognostic lncRNAs screened by Cox regression analysis.
