# Supplementary material for: Development of a Prognostic Model Based on the Identification of EMT-Related lncRNAs in Triple-Negative Breast Cancer
Source: J Oncol. 2021 Nov 27;2021:9219961. doi: 10.1155/2021/9219961 (PMC8643262; doi:10.1155/2021/9219961)
Supplement: Supplementary Materials — Supplementary Figure 1: prognostic lncRNAs were screened out from TNBC data. (A) Cox univariate regression analysis. (B) Lasso regression analysis. Supplementary Figure 2: CeRNA network. Supplementary Table 1: primers used in qRT-PCR. Supplementary Table 2: a total of 1033 lncRNAs highly associated with EMT. Supplementary Table 3: a total of 285 prognostic lncRNAs screened by Cox regression analysis. [file 9219961.f1.zip › 9219961.f1/Table S1 (1).docx]

**Supplementary Table 1. The sequence for primers**

| Gene | Sequence |
| --- | --- |
| NIFK-F | CTGTTTGGTGAAAGACTCTTGG |
| NIFK-R | TCCGATTCCGATTATACCGTTT |
| LINC01315-F | TTATGACATCATGGCTGGCTGCTG |
| LINC01315-R | AATTCTCCTGACTTCACGCCTGTTAC |
| LINC00667-F | CGCCTGTTCTCGCCAATCTCTATG |
| LINC00667-R | CGTGATTCTGGGAGGTCCATTCAAC |
| ASB16-F | ATGATTGTGGAGACTGTGAGC |
| ASB16-R | CACAGTCTGTGTAGCCTCG |
| PINK1-F | CAGGGCAGTGATTGACTACAG |
| PINK1-R | GATTGACAAGCCCGAAGATTTC |
| RGS5-F | GTCATTCCGTACAATGAGAAGC |
| RGS5-R | GCAATCCAGAACTCAAGGTTTT |
| UBE2E2-F | AGGTTACCTTCCGAACAAGAAT |
| UBE2E2-R | AAATAGTTAAAGCCGGACTCCA |
| YTHDF3-F | AGCAGCAGTGGTATGACTAGCATTG |
| YTHDF3-R | GGGTTTAAGTTTCGGTTGAGGTTTGG |
| ZSCAN16-F | CAGTTCCTGAGCATTCTTCCTA |
| ZSCAN16-R | GAGTATGTCCCGTCTTTCTGAA |
| SOCS2-F | AGATAGCTCGCATTCAGACTAC |
| SOCS2-R | TCCTTGCACATCTGAACATAGT |
| TINCR-F | CTGCTGTGTGACTTTGAGGTTGTTG |
| TINCR-R | TCTGTGCTTCTCTTCTTGCGATGC |
| NDUFB2-F | CTCATGTGGTTCTGGATTCTCT |
| NDUFB2-R | GAAGGATCAGGATACGGAAAGT |
| human-GAPDH-F | CAGGAGGCATTGCTGATGAT |
| human-GAPDH-R | GAAGGCTGGGGCTCATTT |
