# Supplementary material for: Development of a Prognostic Model Based on the Identification of EMT-Related lncRNAs in Triple-Negative Breast Cancer
Source: J Oncol. 2021 Nov 27;2021:9219961. doi: 10.1155/2021/9219961 (PMC8643262; doi:10.1155/2021/9219961)
Supplement: Supplementary Materials — Supplementary Figure 1: prognostic lncRNAs were screened out from TNBC data. (A) Cox univariate regression analysis. (B) Lasso regression analysis. Supplementary Figure 2: CeRNA network. Supplementary Table 1: primers used in qRT-PCR. Supplementary Table 2: a total of 1033 lncRNAs highly associated with EMT. Supplementary Table 3: a total of 285 prognostic lncRNAs screened by Cox regression analysis. [file 9219961.f1.zip › 9219961.f1/Table S2 (1).pdf]

lncRNA	Gene	cor	p
A2M-AS1	CXCL12	0.599261	≤0.0001
A2M-AS1	FZD4	0.716116	≤0.0001
A2M-AS1	FGF2	0.66626	≤0.0001
A2M-AS1	TWIST2	0.573254	≤0.0001
A2M-AS1	ZEB1	0.630537	≤0.0001
A2M-AS1	BIRC5	-0.42302	3.11E-13
A2M-AS1	TWIST1	0.370966	2.67E-10
AADACL2-	CDH2	0.342182	6.90E-09
AATBC	CXCL12	-0.30562	2.74E-07
AATBC	TWIST2	-0.30165	3.96E-07
AATBC	FGF2	-0.30018	4.54E-07
ABCA9-AS	ZEB1	0.341824	7.17E-09
AC000067	TGFB1	0.582103	≤0.0001
AC000123	ZEB1	0.354847	1.72E-09
AC002066	FZD4	0.67322	≤0.0001
AC002066	FGF2	0.528305	≤0.0001
AC002066	TWIST2	0.596621	≤0.0001
AC002066	ZEB1	0.692337	≤0.0001
AC002066	CXCL12	0.379329	9.76E-11
AC002066	TWIST1	0.373554	1.96E-10
AC002066	BIRC5	-0.30984	1.83E-07
AC002401	CDH2	0.346139	4.50E-09
AC003984	FZD4	0.452435	4.00E-15
AC003984	TWIST2	0.404423	3.98E-12
AC003984	ZEB1	0.381558	7.42E-11
AC003984	FGF2	0.353464	2.01E-09
AC003985	TGFB1	0.467855	4.44E-16
AC004053	EGF	0.312222	1.46E-07
AC004490	FZD4	0.774386	≤0.0001
AC004490	FGF2	0.62757	≤0.0001
AC004490	TWIST2	0.689636	≤0.0001
AC004490	ZEB1	0.678358	≤0.0001
AC004490	TWIST1	0.595684	≤0.0001
AC004490	CXCL12	0.43902	3.04E-14
AC004490	BIRC5	-0.35975	9.86E-10
AC004637	PDGFB	0.368165	3.72E-10
AC004947	CXCL12	0.691953	≤0.0001
AC004947	BIRC5	-0.53898	≤0.0001
AC004947	FZD4	0.587734	≤0.0001
AC004947	FGF2	0.567225	≤0.0001
AC004947	TWIST2	0.528549	≤0.0001
AC004947	ZEB1	0.56693	≤0.0001
AC004947	TWIST1	0.344455	5.40E-09
AC004947	CXCR4	-0.31614	9.98E-08
AC005256	HIF1A	0.309685	1.86E-07
AC005394	CDH2	0.338955	9.75E-09
AC005498	BIRC5	-0.44939	6.22E-15
AC005498	CXCL12	0.374939	1.66E-10
AC005592	ZEB1	0.417256	6.97E-13
AC006041	ZEB1	0.300881	4.26E-07
AC006150	CDH2	0.351672	2.45E-09
AC006378	BIRC5	-0.37736	1.24E-10
AC006378	ZEB1	0.315508	1.06E-07
AC006538	BIRC5	0.454771	2.66E-15
AC006538	ZEB1	-0.4234	2.95E-13
AC006538	CXCL12	-0.40447	3.96E-12
AC006538	FGF2	-0.37989	9.11E-11

AC006538	FZD4	-0.3552	1.65E-09
AC006840	CDH2	0.353558	1.98E-09
AC007098	CDH2	0.37287	2.13E-10
AC007106	HIF1A	0.373323	2.02E-10
AC007364	ZEB1	0.332788	1.86E-08
AC007557	CDH2	0.305657	2.73E-07
AC007563	BIRC5	-0.38047	8.49E-11
AC007563	CXCL12	0.363007	6.78E-10
AC007563	FZD4	0.352754	2.17E-09
AC007563	ZEB1	0.332218	1.98E-08
AC007563	FGF2	0.323294	4.91E-08
AC007563	BIRC5	-0.38163	7.36E-11
AC007563	CXCL12	0.359853	9.74E-10
AC007563	FZD4	0.343218	6.18E-09
AC007563	ZEB1	0.324818	4.21E-08
AC007563	FGF2	0.319947	6.86E-08
AC007743	CXCL12	0.540552	≤0.0001
AC007743	FZD4	0.714723	≤0.0001
AC007743	FGF2	0.59979	≤0.0001
AC007743	TWIST2	0.517451	≤0.0001
AC007743	ZEB1	0.66224	≤0.0001
AC007743	BIRC5	-0.37768	1.19E-10
AC007792	ZEB1	0.432508	7.95E-14
AC007792	FZD4	0.355322	1.63E-09
AC007792	CXCL12	0.321641	5.79E-08
AC007792	FGF2	0.301412	4.05E-07
AC007950	ZEB1	0.35372	1.95E-09
AC007952	CDH2	0.336449	1.27E-08
AC008175	CXCR4	0.30999	1.81E-07
AC008268	BIRC5	-0.44183	2.00E-14
AC008268	EGF	0.416397	7.85E-13
AC008268	CXCL12	0.362826	6.93E-10
AC008937	ZEB1	0.389245	2.85E-11
AC008991	FZD4	0.598427	≤0.0001
AC008991	FGF2	0.53697	≤0.0001
AC008991	TWIST2	0.488322	≤0.0001
AC008991	ZEB1	0.548559	≤0.0001
AC008991	CXCL12	0.464987	4.44E-16
AC008991	TWIST1	0.397138	1.04E-11
AC008991	BIRC5	-0.35785	1.22E-09
AC009110	CDH2	0.354216	1.84E-09
AC009120	CDH2	0.350006	2.95E-09
AC009299	TWIST2	0.354016	1.89E-09
AC009299	EGF	0.312216	1.46E-07
AC009495	EGF	0.358247	1.17E-09
AC009495	TWIST2	-0.40095	6.30E-12
AC009495	FZD4	-0.33384	1.67E-08
AC009495	TWIST1	-0.30975	1.85E-07
AC009498	CXCL12	0.369295	3.25E-10
AC009506	BIRC5	-0.45248	4.00E-15
AC009506	CXCL12	0.331815	2.06E-08
AC009506	EGF	0.329427	2.64E-08
AC009506	SNAI1	-0.31354	1.28E-07
AC010468	CDH2	0.319502	7.17E-08
AC010731	ZEB1	0.348996	3.29E-09
AC010731	CDH2	0.338948	9.75E-09
AC010745	EGF	0.645447	≤0.0001
AC010745	BIRC5	-0.3973	1.01E-11

AC010745	CXCL12	0.397099	1.04E-11
AC010969	CXCL12	0.494305	≤0.0001
AC010969	FZD4	0.698896	≤0.0001
AC010969	FGF2	0.536258	≤0.0001
AC010969	TWIST2	0.607379	≤0.0001
AC010969	ZEB1	0.622988	≤0.0001
AC010969	BIRC5	-0.35814	1.18E-09
AC010975	CDH2	0.423589	2.87E-13
AC010976	CXCL12	0.481617	≤0.0001
AC010976	FZD4	0.542955	≤0.0001
AC010976	ZEB1	0.488806	≤0.0001
AC010976	TWIST2	0.452798	3.77E-15
AC010976	FGF2	0.387026	3.76E-11
AC010976	BIRC5	-0.35959	1.00E-09
AC010976	TWIST1	0.322035	5.57E-08
AC011239	ZEB1	0.410366	1.79E-12
AC011306	FZD4	0.601022	≤0.0001
AC011306	FGF2	0.480363	≤0.0001
AC011306	ZEB1	0.570924	≤0.0001
AC011306	TWIST2	0.420983	4.14E-13
AC011306	CXCL12	0.4011	6.18E-12
AC011306	BIRC5	-0.34121	7.66E-09
AC011524	CXCL12	0.343734	5.84E-09
AC011524	ZEB1	0.330082	2.46E-08
AC011524	CXCL12	0.304129	3.15E-07
AC011625	FZD4	0.452334	4.00E-15
AC011625	TWIST2	0.386861	3.84E-11
AC011625	ZEB1	0.354991	1.69E-09
AC011625	FGF2	0.337653	1.12E-08
AC011995	FZD4	0.390322	2.48E-11
AC011995	TWIST2	0.365759	4.93E-10
AC011998	TGFB1	0.362603	7.11E-10
AC012368	EGF	0.448816	6.88E-15
AC012368	BIRC5	-0.315	1.11E-07
AC012462	TGFB1	0.437714	3.69E-14
AC012462	CDH2	0.32201	5.58E-08
AC012462	TGFB1	0.358957	1.08E-09
AC012613	ZEB1	0.461185	8.88E-16
AC013448	CDH2	0.349826	3.00E-09
AC016644	CDH2	0.343345	6.09E-09
AC016716	CDH2	0.439237	2.95E-14
AC016910	ZEB1	0.533725	≤0.0001
AC016910	FZD4	0.362178	7.46E-10
AC016910	FGF2	0.3442	5.55E-09
AC016910	CXCL12	0.329965	2.49E-08
AC016910	BIRC5	-0.30194	3.86E-07
AC017002	CXCL12	-0.30139	4.06E-07
AC018685	CDH2	0.319786	6.97E-08
AC018685	HIF1A	0.337512	1.14E-08
AC022182	CXCL12	-0.30981	1.84E-07
AC022182	FGF2	-0.30831	2.12E-07
AC022182	BIRC5	0.308296	2.13E-07
AC024028	CDH2	0.351597	2.47E-09
AC024560	TWIST2	-0.32543	3.96E-08
AC024560	FZD4	-0.30921	1.95E-07
AC026904	TGFB1	0.479873	≤0.0001
AC027601	BIRC5	0.332001	2.02E-08
AC034228	SNAI1	0.309977	1.81E-07

AC055764	CDH2	0.354642	1.76E-09
AC062017	TWIST2	0.526199	⩽0.0001
AC062017	FZD4	0.453022	3.55E-15
AC062017	TWIST1	0.395845	1.23E-11
AC062017	FGF2	0.313914	1.24E-07
AC062017	HIF1A	-0.30249	3.67E-07
AC064834	CDH2	0.330587	2.34E-08
AC067956	CDH2	0.357475	1.28E-09
AC068057	EGF	0.515871	⩽0.0001
AC068057	BIRC5	-0.42513	2.30E-13
AC068057	CXCL12	0.357839	1.23E-09
AC068489	CDH2	0.317342	8.87E-08
AC073130	ZEB1	0.348173	3.60E-09
AC073130	ZEB1	0.532268	⩽0.0001
AC073130	FZD4	0.434959	5.55E-14
AC073130	TWIST2	0.405714	3.35E-12
AC073130	CXCL12	0.365532	5.06E-10
AC073130	BIRC5	-0.33675	1.23E-08
AC073130	FGF2	0.331137	2.21E-08
AC073316	CDH2	0.31789	8.40E-08
AC073316	ZEB1	0.415467	8.93E-13
AC073409	HIF1A	0.380382	8.58E-11
AC073409	SNAI1	0.305458	2.78E-07
AC073626	CDH2	0.353102	2.09E-09
AC073636	BIRC5	-0.35208	2.34E-09
AC073636	EGF	0.347941	3.70E-09
AC073636	ZEB1	0.330603	2.33E-08
AC073636	CXCL12	0.324919	4.17E-08
AC074286	CXCL12	0.555042	⩽0.0001
AC074286	BIRC5	-0.44238	1.84E-14
AC074286	EGF	0.40291	4.87E-12
AC074286	ZEB1	0.349293	3.19E-09
AC079154	CDH2	0.349806	3.01E-09
AC079354	CXCL12	-0.43268	7.77E-14
AC079354	BIRC5	0.382095	6.95E-11
AC079354	ZEB1	-0.35993	9.66E-10
AC079354	FZD4	-0.31792	8.38E-08
AC079354	FGF2	-0.31469	1.15E-07
AC079354	TWIST2	-0.30741	2.31E-07
AC083843	EGF	0.384259	5.32E-11
AC083843	BIRC5	-0.32371	4.71E-08
AC083900	ZEB1	0.352387	2.26E-09
AC084125	BIRC5	0.41439	1.04E-12
AC084125	CXCL12	-0.38255	6.57E-11
AC084125	FGF2	-0.3498	3.01E-09
AC084125	ZEB1	-0.34499	5.10E-09
AC084125	FZD4	-0.34001	8.71E-09
AC090616	CDH2	0.410229	1.83E-12
AC092162	ZEB1	0.383967	5.51E-11
AC092162	CXCL12	0.3209	6.24E-08
AC092168	CDH2	0.306561	2.50E-07
AC092171	FGF2	-0.33356	1.72E-08
AC092171	ZEB1	-0.3123	1.45E-07
AC092171	FGF2	-0.37813	1.13E-10
AC092171	CXCL12	-0.3417	7.27E-09
AC092171	FZD4	-0.31317	1.33E-07
AC092535	CDH2	0.300512	4.41E-07
AC092687	ZEB1	-0.38829	3.21E-11

AC092687	CXCL12	-0.37969	9.34E-11
AC092687	BIRC5	0.361663	7.92E-10
AC092687	FZD4	-0.33027	2.42E-08
AC092687	FGF2	-0.32402	4.57E-08
AC093382	EGF	0.348769	3.37E-09
AC093382	BIRC5	-0.31035	1.75E-07
AC093484	CDH2	0.35706	1.34E-09
AC093843	CDH2	0.348099	3.63E-09
AC098617	ZEB1	0.372869	2.13E-10
AC099754	EGF	0.41672	7.51E-13
AC099754	BIRC5	-0.33113	2.21E-08
AC099754	CXCL12	0.308809	2.02E-07
AC104781	CDH2	0.359554	1.01E-09
AC104794	CXCL12	0.413997	1.09E-12
AC105760	CXCL12	0.475126	≤0.0001
AC105760	BIRC5	-0.50829	≤0.0001
AC105760	ZEB1	0.440122	2.58E-14
AC105760	EGF	0.41764	6.61E-13
AC105760	FGF2	0.393366	1.69E-11
AC105760	SNAI1	-0.31696	9.21E-08
AC108025	CXCL12	-0.42545	2.20E-13
AC108025	BIRC5	0.407843	2.52E-12
AC108025	FGF2	-0.38715	3.71E-11
AC108025	FZD4	-0.31937	7.26E-08
AC108025	TWIST2	-0.31305	1.35E-07
AC108142	CDH2	0.354514	1.78E-09
AC113167	ZEB1	0.356018	1.51E-09
AC114752	CDH2	0.344035	5.65E-09
AC116035	CXCL12	0.339665	9.04E-09
AC116035	ZEB1	0.311657	1.54E-07
AC116035	FZD4	0.305472	2.78E-07
AC133785	CXCL12	-0.34004	8.68E-09
AC133785	ZEB1	-0.30336	3.38E-07
AC133785	HIF1A	0.302745	3.58E-07
AC135050	ZEB1	0.349249	3.20E-09
AC138035	EGF	0.323323	4.90E-08
AC139887	ZEB1	0.304163	3.14E-07
AC144652	CXCL12	-0.36109	8.45E-10
AC144652	ZEB1	-0.30192	3.87E-07
AC144831	CXCL12	0.48094	≤0.0001
AC144831	FZD4	0.483715	≤0.0001
AC144831	TWIST2	0.490449	≤0.0001
AC144831	ZEB1	0.486792	≤0.0001
AC144831	FGF2	0.40205	5.45E-12
AC144831	BIRC5	-0.38548	4.56E-11
AC144833	CDH2	0.355925	1.52E-09
AC226118	EGF	0.539441	≤0.0001
ACTA2-AS	EGF	0.61527	≤0.0001
ACTA2-AS	BIRC5	-0.45433	2.89E-15
ACTA2-AS	CXCL12	0.355185	1.65E-09
ACVR2B-A	EGF	0.41894	5.52E-13
ADAMTS9	ZEB1	0.563213	≤0.0001
ADAMTS9	TWIST2	0.339534	9.16E-09
ADAMTS9	TWIST1	0.336609	1.25E-08
ADAMTS9	FZD4	0.324352	4.41E-08
ADAMTS9	FGF2	0.313758	1.26E-07
ADAMTS9	CXCL12	0.495848	≤0.0001
ADAMTS9	BIRC5	-0.50514	≤0.0001

ADAMTS9	EGF	0.499267	≤0.0001
ADAMTS9	ZEB1	0.490669	≤0.0001
ADAMTS9	FGF2	0.410084	1.86E-12
ADAMTS9	SNAI1	-0.30257	3.64E-07
ADD3-AS1	CXCL12	0.715512	≤0.0001
ADD3-AS1	BIRC5	-0.52255	≤0.0001
ADD3-AS1	ZEB1	0.50014	≤0.0001
ADD3-AS1	FGF2	0.413035	1.25E-12
ADD3-AS1	FZD4	0.357505	1.27E-09
ADD3-AS1	TWIST2	0.31374	1.26E-07
ADIPOQ-A	FZD4	0.841756	≤0.0001
ADIPOQ-A	FGF2	0.662253	≤0.0001
ADIPOQ-A	TWIST2	0.647185	≤0.0001
ADIPOQ-A	ZEB1	0.623139	≤0.0001
ADIPOQ-A	TWIST1	0.498423	≤0.0001
ADIPOQ-A	CXCL12	0.40617	3.15E-12
ADIPOQ-A	BIRC5	-0.32496	4.15E-08
ADIRF-AS1	CXCL12	0.494275	≤0.0001
ADIRF-AS1	BIRC5	-0.50318	≤0.0001
ADIRF-AS1	FGF2	0.469666	2.22E-16
ADIRF-AS1	FZD4	0.459101	1.33E-15
ADIRF-AS1	TWIST2	0.389292	2.83E-11
ADIRF-AS1	ZEB1	0.388348	3.19E-11
ADIRF-AS1	CXCR4	-0.31773	8.54E-08
ADIRF-AS1	SNAI1	-0.31574	1.04E-07
ADIRF-AS1	HIF1A	-0.30643	2.53E-07
ADPGK-AS	CDH2	0.329747	2.55E-08
AF001548.	BIRC5	-0.33112	2.21E-08
AF038458.	CDH2	0.330803	2.29E-08
AF067845.	FZD4	0.681914	≤0.0001
AF067845.	FGF2	0.589433	≤0.0001
AF067845.	TWIST2	0.674591	≤0.0001
AF067845.	ZEB1	0.620523	≤0.0001
AF067845.	TWIST1	0.572015	≤0.0001
AF067845.	CXCL12	0.450159	5.77E-15
AF067845.	BIRC5	-0.3682	3.70E-10
AF131215.	BIRC5	-0.39314	1.74E-11
AF131215.	EGF	0.361685	7.90E-10
AF131215.	CXCL12	0.300936	4.24E-07
AF230666.	CDH2	0.300793	4.29E-07
AFAP1-AS	BIRC5	0.396597	1.11E-11
AFAP1-AS	CXCL12	-0.34786	3.73E-09
AFF2-IT1	CDH2	0.354456	1.79E-09
AGAP11	CXCL12	0.645919	≤0.0001
AGAP11	BIRC5	-0.50828	≤0.0001
AGAP11	FZD4	0.781578	≤0.0001
AGAP11	FGF2	0.737794	≤0.0001
AGAP11	TWIST2	0.556099	≤0.0001
AGAP11	ZEB1	0.728432	≤0.0001
AGAP11	TWIST1	0.38017	8.80E-11
AGAP11	CXCR4	-0.32554	3.91E-08
AGAP2-AS	ZEB1	-0.39429	1.50E-11
AGAP2-AS	FZD4	-0.31042	1.74E-07
AGBL4-IT1	CDH2	0.353981	1.89E-09
AGBL5-IT1	CXCL12	0.318568	7.86E-08
AGBL5-IT1	BIRC5	-0.30482	2.95E-07
AIRN	CDH2	0.313561	1.28E-07
AJ011932.1	TWIST2	0.35974	9.87E-10

AL589986.	CDH2	0.30359	3.31E-07
ALDH1L1-	FZD4	0.759784	≤0.0001
ALDH1L1-	FGF2	0.621337	≤0.0001
ALDH1L1-	TWIST2	0.69319	≤0.0001
ALDH1L1-	ZEB1	0.571639	≤0.0001
ALDH1L1-	TWIST1	0.666907	≤0.0001
ALDH1L1-	CXCL12	0.405864	3.29E-12
ALDH1L1-	BIRC5	-0.34599	4.58E-09
ALG13-AS	ZEB1	0.344858	5.17E-09
ALG1L9P	BIRC5	0.35487	1.71E-09
ALG1L9P	FZD4	-0.34829	3.56E-09
ALG1L9P	ZEB1	-0.32854	2.88E-08
ALG1L9P	TWIST2	-0.32104	6.15E-08
ALKBH3-A	ZEB1	0.326341	3.61E-08
ALMS1-IT:	CXCL12	-0.32438	4.40E-08
ALOX12-A	EGF	0.328246	2.97E-08
ANKRD62I	EGF	0.471259	2.22E-16
AP000345.	CDH2	0.354371	1.81E-09
AP000345.	EGF	0.37022	2.92E-10
AP000439.	CXCL12	0.506033	≤0.0001
AP000439.	BIRC5	-0.45832	1.55E-15
AP000439.	EGF	0.362156	7.48E-10
AP000439.	CXCL12	0.340741	8.06E-09
AP000442.	ZEB1	0.41587	8.44E-13
AP000442.	FZD4	0.35227	2.29E-09
AP000442.	FGF2	0.310438	1.73E-07
AP001057.	EGF	0.316087	1.00E-07
AP001063.	BIRC5	0.381188	7.77E-11
AP001189.	CXCL12	0.361921	7.69E-10
AP001257.	ZEB1	0.571829	≤0.0001
AP001257.	FZD4	0.468561	4.44E-16
AP001257.	CXCL12	0.41567	8.68E-13
AP001257.	FGF2	0.409476	2.02E-12
AP001257.	TWIST2	0.372249	2.29E-10
AP001412.	BIRC5	0.45113	4.88E-15
AP001412.	CXCL12	-0.44171	2.04E-14
AP001412.	ZEB1	-0.41647	7.77E-13
AP001412.	FZD4	-0.35727	1.31E-09
AP001412.	FGF2	-0.33044	2.37E-08
AP001429.	CDH2	0.32416	4.50E-08
AP001471.	TGFB1	0.345447	4.85E-09
AP001476.	TGFB1	0.544954	≤0.0001
AP001476.	TWIST1	0.351956	2.37E-09
AP001476.	TGFB1	0.49562	≤0.0001
AP001627.	FZD4	0.598274	≤0.0001
AP001627.	FGF2	0.479133	≤0.0001
AP001627.	TWIST2	0.561161	≤0.0001
AP001627.	ZEB1	0.596546	≤0.0001
AP001627.	CXCL12	0.461893	8.88E-16
AP001627.	BIRC5	-0.4541	3.11E-15
AP001627.	TWIST1	0.361484	8.08E-10
AP002856.	EGF	0.480104	≤0.0001
AP003774.	CXCR4	0.308382	2.11E-07
AP004782.	CXCL12	0.497049	≤0.0001
AP004782.	FZD4	0.781194	≤0.0001
AP004782.	FGF2	0.682774	≤0.0001
AP004782.	TWIST2	0.655191	≤0.0001
AP004782.	ZEB1	0.631297	≤0.0001

AP004782.	TWIST1	0.500995	≤0.0001
AP004782.	BIRC5	-0.41995	4.79E-13
APTR	TWIST2	-0.32317	4.97E-08
APTR	ZEB1	-0.31396	1.23E-07
APTR	FZD4	-0.30158	3.99E-07
AQP4-AS1	EGF	0.348229	3.58E-09
ARHGAP3	CXCL12	-0.36285	6.91E-10
ARHGAP3	BIRC5	0.301065	4.19E-07
ARHGAP5	EGF	0.402109	5.41E-12
ARHGAP5	BIRC5	-0.36823	3.69E-10
ARHGEF26	CXCL12	-0.36346	6.44E-10
ARHGEF26	TWIST2	-0.32426	4.45E-08
ARHGEF26	FGF2	-0.30735	2.32E-07
ARHGEF26	FZD4	-0.30501	2.90E-07
ARHGEF7-	ZEB1	0.554278	≤0.0001
ARHGEF7-	FZD4	0.36441	5.77E-10
ARHGEF7-	FGF2	0.305141	2.86E-07
ARHGEF7-	CXCL12	0.54544	≤0.0001
ARHGEF7-	FZD4	0.837339	≤0.0001
ARHGEF7-	FGF2	0.70673	≤0.0001
ARHGEF7-	TWIST2	0.786522	≤0.0001
ARHGEF7-	ZEB1	0.724198	≤0.0001
ARHGEF7-	TWIST1	0.658122	≤0.0001
ARHGEF7-	BIRC5	-0.43188	8.73E-14
ARHGEF7-	ZEB1	0.351722	2.44E-09
ARHGEF7-	CDH2	0.349449	3.13E-09
ARMC2-A	ZEB1	0.400822	6.41E-12
ARMC2-A	BIRC5	-0.30509	2.88E-07
ARMCX3-/	CDH2	0.309083	1.97E-07
ARPP21-A	CDH2	0.322277	5.44E-08
ARRDC1-/	TGFB1	0.352104	2.33E-09
ARRDC1-/	ZEB1	-0.34479	5.21E-09
ARRDC1-/	FGF2	-0.31956	7.13E-08
ASB16-AS	FZD4	-0.33297	1.83E-08
ASB16-AS	TWIST2	-0.31836	8.02E-08
ASH1L-AS	FZD4	-0.41329	1.20E-12
ASH1L-AS	ZEB1	-0.38141	7.56E-11
ASH1L-AS	CXCL12	-0.37211	2.33E-10
ASH1L-AS	BIRC5	0.371642	2.46E-10
ASH1L-AS	FGF2	-0.33353	1.72E-08
ASH1L-AS	TWIST2	-0.31567	1.04E-07
ASTN2-AS	CDH2	0.323587	4.77E-08
ATP13A5-	CXCL12	-0.31825	8.11E-08
ATP1A1-A	EGF	0.427018	1.76E-13
ATP1A1-A	BIRC5	-0.31664	9.50E-08
ATP1B3-A	ZEB1	0.415966	8.33E-13
ATP2A1-A	CXCL12	-0.47487	≤0.0001
ATP2A1-A	BIRC5	0.528179	≤0.0001
ATP2A1-A	ZEB1	-0.43977	2.73E-14
ATP2A1-A	FGF2	-0.40301	4.80E-12
ATP2A1-A	FZD4	-0.36728	4.13E-10
ATP2A1-A	TWIST2	-0.3227	5.21E-08
ATP2B2-IT	CDH2	0.336657	1.24E-08
B3GALT5-	EGF	0.507451	≤0.0001
B4GALT1-	CXCL12	0.510187	≤0.0001
B4GALT1-	FZD4	0.829466	≤0.0001
B4GALT1-	FGF2	0.638487	≤0.0001
B4GALT1-	TWIST2	0.73524	≤0.0001

B4GALT1-	ZEB1	0.619473	≤0.0001
B4GALT1-	TWIST1	0.412679	1.31E-12
B4GALT1-	BIRC5	-0.38338	5.93E-11
BAALC-AS	TGFB1	0.32172	5.75E-08
BACE1-AS	TGFB1	-0.31176	1.53E-07
BCDIN3D-	CXCL12	0.499633	≤0.0001
BCDIN3D-	ZEB1	0.491767	≤0.0001
BCDIN3D-	BIRC5	-0.44827	7.55E-15
BCDIN3D-	FGF2	0.422999	3.12E-13
BCDIN3D-	FZD4	0.398692	8.47E-12
BCDIN3D-	SNAI1	-0.34375	5.83E-09
BCDIN3D-	EGF	0.319085	7.47E-08
BDNF-AS	BIRC5	-0.48937	≤0.0001
BDNF-AS	EGF	0.514913	≤0.0001
BDNF-AS	CXCL12	0.464156	6.66E-16
BDNF-AS	SNAI1	-0.39436	1.48E-11
BDNF-AS	FGF2	0.357749	1.24E-09
BDNF-AS	TGFB1	-0.32333	4.89E-08
BDNF-AS	HIF1A	-0.31286	1.37E-07
BEAN1-AS	BIRC5	-0.37687	1.32E-10
BEAN1-AS	ZEB1	0.346245	4.45E-09
BLACAT1	CXCL12	-0.33607	1.32E-08
BLACAT1	FZD4	-0.30431	3.10E-07
BMS1P14	FZD4	0.497784	≤0.0001
BMS1P14	TWIST2	0.387742	3.44E-11
BMS1P14	ZEB1	0.348981	3.30E-09
BMS1P14	FGF2	0.338677	1.00E-08
BMS1P4	ZEB1	0.306827	2.44E-07
BOK-AS1	FZD4	0.325834	3.80E-08
BRWD1-A	CXCL12	-0.34774	3.78E-09
BVES-AS1	CDH2	0.321368	5.95E-08
C10orf111	FZD4	-0.33445	1.57E-08
C10orf111	TWIST2	-0.32774	3.13E-08
C10orf95	ZEB1	-0.39634	1.15E-11
C10orf95	FGF2	-0.38051	8.44E-11
C10orf95	FZD4	-0.37626	1.42E-10
C10orf95	CXCL12	-0.3592	1.05E-09
C10orf95	BIRC5	0.339782	8.92E-09
C10orf95	TWIST2	-0.33724	1.17E-08
C14orf132	CXCL12	0.447025	9.10E-15
C14orf132	BIRC5	-0.43498	5.55E-14
C14orf132	SNAI1	-0.31547	1.06E-07
C14orf132	EGF	0.307749	2.24E-07
C17orf100	FZD4	-0.35389	1.91E-09
C17orf100	ZEB1	-0.34907	3.26E-09
C17orf100	EGF	0.302457	3.68E-07
C17orf82	TGFB1	0.623815	≤0.0001
C1orf140	ZEB1	0.431719	8.93E-14
C1QTNF9-	CXCL12	0.397922	9.36E-12
C1QTNF9-	BIRC5	-0.33493	1.49E-08
C1QTNF9-	FGF2	0.304604	3.01E-07
C20orf203	EGF	0.519915	≤0.0001
C20orf203	BIRC5	-0.40372	4.37E-12
C20orf203	CXCL12	0.315414	1.07E-07
C21orf62-	BIRC5	-0.48726	≤0.0001
C21orf62-	CXCL12	0.40146	5.89E-12
C21orf62-	EGF	0.362944	6.83E-10
C21orf62-	ZEB1	0.315961	1.02E-07

C2orf48	BIRC5	0.499787	≤0.0001
C2orf48	CXCL12	-0.4513	4.66E-15
C2orf48	FGF2	-0.37554	1.54E-10
C2orf48	FZD4	-0.35242	2.25E-09
C2orf48	ZEB1	-0.32596	3.75E-08
C2orf48	TWIST2	-0.31622	9.90E-08
C3orf67-A	CDH2	0.303317	3.40E-07
C5orf64	CXCL12	0.524355	≤0.0001
C5orf64	FZD4	0.678241	≤0.0001
C5orf64	FGF2	0.542392	≤0.0001
C5orf64	TWIST2	0.580051	≤0.0001
C5orf64	ZEB1	0.532415	≤0.0001
C5orf64	BIRC5	-0.44134	2.15E-14
C6orf99	CXCL12	-0.43383	6.57E-14
C6orf99	FGF2	-0.38798	3.34E-11
C6orf99	ZEB1	-0.38096	7.99E-11
C6orf99	TWIST2	-0.37558	1.54E-10
C6orf99	BIRC5	0.358515	1.14E-09
C6orf99	FZD4	-0.35322	2.06E-09
C6orf99	HIF1A	0.340425	8.33E-09
C6orf99	TWIST1	-0.30996	1.81E-07
C7orf69	ZEB1	0.452449	4.00E-15
C7orf69	FZD4	0.305832	2.68E-07
C9orf139	TGFB1	0.446139	1.04E-14
C9orf163	BIRC5	0.422002	3.59E-13
C9orf163	ZEB1	-0.41511	9.38E-13
C9orf163	TWIST2	-0.39176	2.07E-11
C9orf163	FZD4	-0.38408	5.44E-11
C9orf163	CXCL12	-0.37897	1.02E-10
C9orf163	FGF2	-0.36101	8.54E-10
C9orf170	CDH2	0.363023	6.77E-10
CACNA1C	CDH2	0.336408	1.28E-08
CACNA1C	CDH2	0.322611	5.26E-08
CACNA2D	CDH2	0.353992	1.89E-09
CACTIN-A	TGFB1	0.309546	1.89E-07
CADM2-A	CDH2	0.360465	9.09E-10
CADM2-A	ZEB1	0.313589	1.28E-07
CADM2-A	CDH2	0.352945	2.13E-09
CADM3-A	CXCL12	0.831921	≤0.0001
CADM3-A	BIRC5	-0.61939	≤0.0001
CADM3-A	FZD4	0.664822	≤0.0001
CADM3-A	FGF2	0.650977	≤0.0001
CADM3-A	TWIST2	0.537913	≤0.0001
CADM3-A	ZEB1	0.667016	≤0.0001
CADM3-A	TWIST1	0.351247	2.57E-09
CARD8-AS	ZEB1	0.32049	6.50E-08
CARD8-AS	CXCL12	0.306807	2.45E-07
CASC15	CDH2	0.387163	3.70E-11
CASC19	CDH2	0.326068	3.71E-08
CASC2	TGFB1	-0.30302	3.49E-07
CASC22	TWIST1	0.470405	2.22E-16
CCDC144N	CDH2	0.353434	2.01E-09
CCDC39-A	ZEB1	0.388636	3.07E-11
CCDC39-A	FGF2	0.368488	3.58E-10
CCDC39-A	CXCL12	0.34834	3.54E-09
CCDC39-A	BIRC5	-0.32674	3.47E-08
CCDC39-A	FZD4	0.308969	1.99E-07
CCND2-AS	ZEB1	0.528298	≤0.0001

CCND2-A:	BIRC5	-0.39526	1.32E-11
CCND2-A:	CXCL12	0.333535	1.72E-08
CCNT2-AS	EGF	0.330486	2.36E-08
CD27-AS1	ZEB1	-0.33684	1.22E-08
CD27-AS1	CDH2	0.313852	1.25E-07
CDKN2B-/	CXCL12	-0.36989	3.03E-10
CDKN2B-/	BIRC5	0.337337	1.16E-08
CEBPB-AS	CXCL12	0.488005	≤0.0001
CEBPB-AS	BIRC5	-0.50081	≤0.0001
CEBPB-AS	ZEB1	0.434808	5.68E-14
CEBPB-AS	FGF2	0.364057	6.01E-10
CEBPB-AS	FZD4	0.329576	2.60E-08
CEBPB-AS	CXCR4	-0.31828	8.08E-08
CECR3	CDH2	0.321682	5.77E-08
CECR7	CDH2	0.320606	6.42E-08
CELF2-AS:	ZEB1	0.374522	1.75E-10
CELF2-AS:	ZEB1	0.301934	3.86E-07
CERS3-AS	ZEB1	0.3989	8.24E-12
CERS3-AS	CXCL12	0.317352	8.86E-08
CERS3-AS	BIRC5	-0.30772	2.24E-07
CH17-340	TGFB1	0.304746	2.97E-07
CHL1-AS1	CXCL12	0.60361	≤0.0001
CHL1-AS1	BIRC5	-0.50611	≤0.0001
CHL1-AS1	FZD4	0.513294	≤0.0001
CHL1-AS1	FGF2	0.540609	≤0.0001
CHL1-AS1	ZEB1	0.592219	≤0.0001
CHL1-AS1	TWIST2	0.402261	5.30E-12
CHL1-AS2	CXCL12	0.663309	≤0.0001
CHL1-AS2	BIRC5	-0.53411	≤0.0001
CHL1-AS2	TWIST2	0.45194	4.22E-15
CHL1-AS2	FGF2	0.419306	5.24E-13
CHL1-AS2	FZD4	0.395	1.37E-11
CHL1-AS2	TWIST1	0.358325	1.16E-09
CHL1-AS2	ZEB1	0.332602	1.90E-08
CHL1-AS2	HIF1A	-0.3119	1.50E-07
CHRM3-A	CDH2	0.370689	2.76E-10
CIRBP-AS:	FGF2	-0.30479	2.96E-07
CKMT2-A:	CXCL12	0.500657	≤0.0001
CKMT2-A:	BIRC5	-0.55329	≤0.0001
CKMT2-A:	EGF	0.566567	≤0.0001
CKMT2-A:	FGF2	0.335559	1.39E-08
CKMT2-A:	SNAI1	-0.33412	1.62E-08
CKMT2-A:	ZEB1	0.324515	4.34E-08
CLSTN2-A	ZEB1	0.309916	1.82E-07
CNTFR-AS	FZD4	0.697563	≤0.0001
CNTFR-AS	FGF2	0.5702	≤0.0001
CNTFR-AS	TWIST2	0.527498	≤0.0001
CNTFR-AS	ZEB1	0.637874	≤0.0001
CNTFR-AS	CXCL12	0.37681	1.33E-10
CNTFR-AS	TWIST1	0.373165	2.06E-10
CNTFR-AS	BIRC5	-0.31756	8.68E-08
CNTN4-A:	ZEB1	0.317257	8.94E-08
CNTN4-A:	CDH2	0.351006	2.64E-09
COL4A2-/	ZEB1	0.532864	≤0.0001
COL4A2-/	FZD4	0.347882	3.72E-09
COL4A2-/	TWIST2	0.327918	3.07E-08
COL4A2-/	TWIST1	0.314175	1.21E-07
COL4A2-/	ZEB1	0.338042	1.07E-08

COX10-AS	EGF	0.339864	8.85E-09
COX10-AS	SNAI1	-0.32003	6.80E-08
CPB2-AS1	EGF	0.40814	2.42E-12
CPB2-AS1	SNAI1	-0.30203	3.83E-07
CPEB1-AS	ZEB1	0.410212	1.83E-12
CRNDE	EGF	0.31997	6.84E-08
CSPG4P1Y	PDGFB	0.443921	1.47E-14
CTBP1-AS	ZEB1	0.305238	2.84E-07
CTD-2201	CDH2	0.344439	5.41E-09
CTD-2270	EGF	0.51211	≤0.0001
CTD-2270	BIRC5	-0.40152	5.85E-12
CTD-2270	CXCL12	0.345312	4.92E-09
CTD-2270	SNAI1	-0.3126	1.41E-07
CTD-3080	TWIST2	0.339145	9.55E-09
CTD-3080	FZD4	0.331543	2.12E-08
CTD-3080	CXCL12	0.322495	5.32E-08
CYB561D2	TGFB1	0.313192	1.33E-07
CYP4F26P	CDH2	0.307964	2.19E-07
CYYR1-AS	HIF1A	0.41435	1.04E-12
DAB1-AS1	CDH2	0.338988	9.71E-09
DANCR	ZEB1	-0.47909	≤0.0001
DANCR	BIRC5	0.439599	2.80E-14
DANCR	FZD4	-0.40778	2.54E-12
DANCR	CXCL12	-0.40538	3.51E-12
DANCR	FGF2	-0.38005	8.93E-11
DANCR	TWIST2	-0.3464	4.38E-09
DARS-AS1	FZD4	-0.32889	2.78E-08
DARS-AS1	TWIST2	-0.32112	6.10E-08
DARS-AS1	FGF2	-0.31277	1.38E-07
DDC-AS1	HIF1A	0.302135	3.79E-07
DDX11-AS	CXCL12	-0.5077	≤0.0001
DDX11-AS	BIRC5	0.500541	≤0.0001
DDX11-AS	FZD4	-0.40485	3.76E-12
DDX11-AS	CDH2	0.391836	2.05E-11
DDX11-AS	FGF2	-0.38743	3.58E-11
DDX11-AS	TWIST2	-0.37097	2.67E-10
DDX11-AS	ZEB1	-0.37043	2.85E-10
DEPDC1-A	BIRC5	0.491024	≤0.0001
DEPDC1-A	CXCL12	-0.43036	1.09E-13
DEPDC1-A	FGF2	-0.34045	8.31E-09
DEPDC1-A	FZD4	-0.32005	6.79E-08
DEPDC1-A	ZEB1	-0.31539	1.07E-07
DGCR5	BIRC5	0.405187	3.60E-12
DGCR5	CXCL12	-0.39284	1.80E-11
DGCR5	ZEB1	-0.34926	3.20E-09
DGCR5	FZD4	-0.34353	5.97E-09
DGCR5	FGF2	-0.32557	3.90E-08
DGCR9	CXCL12	-0.35307	2.10E-09
DGCR9	BIRC5	0.329059	2.74E-08
DGCR9	FGF2	-0.32	6.82E-08
DGCR9	FZD4	-0.31632	9.80E-08
DGUOK-A	ZEB1	-0.3683	3.66E-10
DGUOK-A	CXCL12	-0.34917	3.23E-09
DGUOK-A	FZD4	-0.3129	1.37E-07
DGUOK-A	FGF2	-0.30248	3.67E-07
DHRS4-AS	TGFB1	-0.31848	7.93E-08
DICER1-AS	EGF	0.315091	1.11E-07
DIO3OS	FZD4	0.537576	≤0.0001

DIO3OS	TWIST2	0.545315	≤0.0001
DIO3OS	TWIST1	0.553686	≤0.0001
DIO3OS	CXCL12	0.451924	4.22E-15
DIO3OS	ZEB1	0.45029	5.55E-15
DIO3OS	FGF2	0.414354	1.04E-12
DIO3OS	BIRC5	-0.37995	9.05E-11
DKFZP434	CDH2	0.322284	5.43E-08
DKFZp779	FZD4	0.436359	4.53E-14
DKFZp779	TWIST2	0.41765	6.60E-13
DLEU2	BIRC5	0.350015	2.94E-09
DLEU2	CXCL12	-0.32781	3.11E-08
DLEU2	CDH2	0.300768	4.30E-07
DLEU2L	ZEB1	0.356941	1.36E-09
DLEU7-AS	TGFB1	0.372766	2.16E-10
DLG5-AS1	ZEB1	-0.30191	3.87E-07
DLGAP1-A	CDH2	0.372281	2.28E-10
DLGAP1-A	CDH2	0.342613	6.59E-09
DLGAP2-A	CDH2	0.367968	3.81E-10
DLX6-AS1	CDH2	0.321007	6.17E-08
DLX6-AS1	CXCL12	-0.30726	2.34E-07
DNM3-IT1	CDH2	0.319592	7.10E-08
DNMBP-A	EGF	0.44112	2.22E-14
DNMBP-A	BIRC5	-0.37449	1.75E-10
DNMBP-A	SNAI1	-0.34415	5.58E-09
DOCK4-AS	CDH2	0.331929	2.04E-08
DPP10-AS	CDH2	0.352292	2.29E-09
DPP10-AS	CDH2	0.353652	1.96E-09
DSCAM-IT	CDH2	0.34271	6.52E-09
DSCR9	CXCL12	-0.32655	3.54E-08
DUXAP8	CXCL12	-0.31859	7.84E-08
EFCAB14-.	ZEB1	0.304866	2.94E-07
EGFR-AS1	CDH2	0.353123	2.08E-09
EHD4-AS1	ZEB1	0.316044	1.01E-07
EIF1B-AS1	CXCL12	0.615106	≤0.0001
EIF1B-AS1	BIRC5	-0.59709	≤0.0001
EIF1B-AS1	FZD4	0.577477	≤0.0001
EIF1B-AS1	FGF2	0.572355	≤0.0001
EIF1B-AS1	TWIST2	0.482689	≤0.0001
EIF1B-AS1	ZEB1	0.657997	≤0.0001
EIF1B-AS1	TWIST1	0.358852	1.09E-09
EIF1B-AS1	CXCR4	-0.32696	3.39E-08
EIF1B-AS1	SNAI1	-0.32088	6.25E-08
EIF1B-AS1	EGF	0.310048	1.80E-07
ELMO1-AS	CDH2	0.34901	3.29E-09
ELOVL2-A	EGF	0.349306	3.18E-09
ELOVL2-A	BIRC5	-0.34201	7.03E-09
EMX2OS	CXCL12	0.632485	≤0.0001
EMX2OS	FZD4	0.748872	≤0.0001
EMX2OS	FGF2	0.660917	≤0.0001
EMX2OS	TWIST2	0.707343	≤0.0001
EMX2OS	ZEB1	0.859927	≤0.0001
EMX2OS	TWIST1	0.506585	≤0.0001
EMX2OS	BIRC5	-0.46574	4.44E-16
ENOX1-AS	CDH2	0.305476	2.77E-07
ENOX1-AS	CDH2	0.353979	1.89E-09
ENTPD3-A	CXCL12	0.45423	3.11E-15
ENTPD3-A	BIRC5	-0.4475	8.44E-15
ENTPD3-A	FGF2	0.385515	4.55E-11

ENTPD3-A	TWIST2	0.37444	1.76E-10
ENTPD3-A	FZD4	0.371657	2.46E-10
ENTPD3-A	HIF1A	-0.3352	1.45E-08
ENTPD3-A	ZEB1	0.332026	2.02E-08
ENTPD3-A	CXCR4	-0.32	6.82E-08
EPB41L4A-	EGF	0.542829	≤0.0001
EPB41L4A-	CXCL12	0.424356	2.57E-13
EPB41L4A-	BIRC5	-0.39854	8.64E-12
EPB41L4A-	HIF1A	-0.39401	1.55E-11
EPHA1-AS	HIF1A	0.332178	1.98E-08
EPHA5-AS	TWIST1	0.303205	3.43E-07
EPN2-AS1	CDH2	0.33877	9.94E-09
ERC2-IT1	CDH2	0.321303	5.99E-08
ERICH6-AS	CDH2	0.330938	2.26E-08
ERVH48-1	PDGFB	0.435221	5.35E-14
ETV5-AS1	CDH2	0.327647	3.16E-08
ETV5-AS1	ZEB1	0.30529	2.82E-07
EWSAT1	EGF	0.387	3.78E-11
EWSAT1	BIRC5	-0.35226	2.29E-09
EXTL3-AS	EGF	0.318032	8.29E-08
FAM138B	CXCL12	0.341248	7.63E-09
FAM13A-A	BIRC5	-0.45616	2.22E-15
FAM13A-A	CXCL12	0.379129	1.00E-10
FAM13A-A	ZEB1	0.374904	1.67E-10
FAM13A-A	EGF	0.330359	2.39E-08
FAM155A-	CDH2	0.337833	1.10E-08
FAM157A	HIF1A	0.340077	8.65E-09
FAM170B-	ZEB1	0.347092	4.06E-09
FAM201A	ZEB1	-0.32398	4.58E-08
FAM201A	FZD4	-0.31495	1.12E-07
FAM222A-	FZD4	0.488984	≤0.0001
FAM222A-	FGF2	0.530674	≤0.0001
FAM222A-	TWIST2	0.438552	3.26E-14
FAM222A-	ZEB1	0.372756	2.16E-10
FAM222A-	TWIST1	0.32715	3.33E-08
FAM225A	TGFB1	0.539794	≤0.0001
FAM225B	TGFB1	0.529201	≤0.0001
FAM41C	EGF	0.34279	6.47E-09
FAM66B	EGF	0.449761	6.00E-15
FAM66B	BIRC5	-0.43213	8.39E-14
FAM66B	SNAI1	-0.36124	8.32E-10
FAM66B	CXCL12	0.358757	1.10E-09
FAM66B	ZEB1	0.314995	1.12E-07
FAM66C	CDH2	0.44326	1.60E-14
FAM66D	EGF	0.413489	1.17E-12
FAM66D	CXCL12	0.323088	5.01E-08
FAM83C-A	CXCL12	-0.42681	1.81E-13
FAM83C-A	BIRC5	0.406456	3.04E-12
FAM83C-A	ZEB1	-0.36552	5.07E-10
FAM83C-A	FZD4	-0.33706	1.19E-08
FAM83C-A	FGF2	-0.32307	5.02E-08
FAM87A	CDH2	0.34906	3.27E-09
FAM87B	ZEB1	0.576436	≤0.0001
FAM87B	FZD4	0.333843	1.67E-08
FAM87B	TWIST2	0.323662	4.73E-08
FEZF1-AS	CDH2	0.473545	≤0.0001
FGD5-AS1	FGF2	0.515658	≤0.0001
FGD5-AS1	ZEB1	0.517119	≤0.0001

FGD5-AS1	FZD4	0.465601	4.44E-16
FGD5-AS1	CXCL12	0.444758	1.29E-14
FGD5-AS1	TWIST2	0.33848	1.02E-08
FGF10-AS	CXCL12	0.640599	≤0.0001
FGF10-AS	BIRC5	-0.53953	≤0.0001
FGF10-AS	FZD4	0.525804	≤0.0001
FGF10-AS	FGF2	0.558526	≤0.0001
FGF10-AS	TWIST2	0.56308	≤0.0001
FGF10-AS	ZEB1	0.660696	≤0.0001
FGF10-AS	TWIST1	0.498779	≤0.0001
FGF10-AS	EGF	0.330918	2.26E-08
FGF12-AS	CDH2	0.31252	1.42E-07
FGF12-AS	FZD4	0.555139	≤0.0001
FGF12-AS	ZEB1	0.465332	4.44E-16
FGF12-AS	FGF2	0.46162	8.88E-16
FGF12-AS	TWIST2	0.374378	1.78E-10
FGF12-AS	CXCL12	0.326319	3.62E-08
FGF12-AS	TWIST1	0.302976	3.51E-07
FGF13-AS	CXCL12	0.525769	≤0.0001
FGF13-AS	FZD4	0.714563	≤0.0001
FGF13-AS	FGF2	0.633872	≤0.0001
FGF13-AS	TWIST2	0.573459	≤0.0001
FGF13-AS	ZEB1	0.65103	≤0.0001
FGF13-AS	BIRC5	-0.47019	2.22E-16
FGF13-AS	TWIST1	0.309238	1.94E-07
FGF13-AS	CXCR4	-0.30307	3.48E-07
FGF14-AS	CDH2	0.357727	1.24E-09
FGF14-AS	CXCL12	0.584969	≤0.0001
FGF14-AS	FZD4	0.740692	≤0.0001
FGF14-AS	FGF2	0.617512	≤0.0001
FGF14-AS	TWIST2	0.691528	≤0.0001
FGF14-AS	ZEB1	0.582491	≤0.0001
FGF14-AS	BIRC5	-0.44977	6.00E-15
FGF14-AS	TWIST1	0.42721	1.71E-13
FGF14-AS	HIF1A	-0.35619	1.48E-09
FGF14-AS	CXCR4	-0.35019	2.89E-09
FGF14-IT1	CDH2	0.356396	1.44E-09
FLG-AS1	ZEB1	0.422354	3.42E-13
FLG-AS1	CDH2	0.312904	1.37E-07
FLJ20021	TWIST1	0.482404	≤0.0001
FLJ20021	TWIST2	0.386596	3.97E-11
FLJ22447	TGFB1	0.3349	1.49E-08
FLJ30679	CXCL12	-0.3117	1.53E-07
FLJ31104	CXCL12	0.376422	1.39E-10
FLJ31104	BIRC5	-0.345	5.09E-09
FLJ31104	EGF	0.344054	5.64E-09
FLJ37035	EGF	0.459551	1.33E-15
FLJ37035	BIRC5	-0.35606	1.50E-09
FLJ46284	BIRC5	-0.36434	5.81E-10
FOXC2-AS	TGFB1	0.302854	3.55E-07
FOXD2-AS	BIRC5	0.36508	5.33E-10
FOXD2-AS	CXCL12	-0.35014	2.90E-09
FOXD3-AS	BIRC5	0.472811	≤0.0001
FOXD3-AS	CXCL12	-0.39525	1.32E-11
FOXD3-AS	ZEB1	-0.34419	5.56E-09
FOXD3-AS	FZD4	-0.30613	2.61E-07
FOXG1-AS	CDH2	0.360938	8.61E-10
FREM2-AS	CDH2	0.332286	1.96E-08

FRMPD3-/	CDH2	0.32277	5.18E-08
FRMPD4-/	CDH2	0.348129	3.62E-09
FTX	ZEB1	0.351048	2.62E-09
GABRG3-/	CDH2	0.355038	1.68E-09
GACAT2	SNAI1	0.331771	2.07E-08
GACAT2	BIRC5	0.305687	2.72E-07
GAS5	ZEB1	-0.31425	1.20E-07
GAS5	PDGFB	-0.30145	4.04E-07
GAS5-AS1	ZEB1	0.420149	4.66E-13
GAS5-AS1	BIRC5	-0.36445	5.74E-10
GAS5-AS1	CXCL12	0.326134	3.69E-08
GAS5-AS1	FGF2	0.301188	4.14E-07
GATA2-AS	HIF1A	0.305508	2.77E-07
GDNF-AS:	CDH2	0.361846	7.75E-10
GHET1	TWIST2	-0.38235	6.73E-11
GHET1	FGF2	-0.33822	1.05E-08
GHET1	FZD4	-0.33351	1.73E-08
GHET1	CXCL12	-0.31357	1.28E-07
GLIDR	CXCL12	0.331035	2.23E-08
GLIDR	BIRC5	-0.33019	2.44E-08
GLIDR	EGF	0.327031	3.37E-08
GLIDR	SNAI1	-0.30661	2.49E-07
GLYCTK-A	ZEB1	0.34293	6.37E-09
GNG12-AS	ZEB1	0.399923	7.21E-12
GNG12-AS	BIRC5	-0.3745	1.75E-10
GPC6-AS1	CDH2	0.305897	2.67E-07
GPC6-AS2	CDH2	0.311949	1.50E-07
GRID1-AS	ZEB1	0.347761	3.77E-09
GRIK1-AS:	BIRC5	-0.34482	5.19E-09
GRIK1-AS:	EGF	0.31084	1.67E-07
GRM7-AS:	CDH2	0.353101	2.09E-09
GRPEL2-A	ZEB1	0.459954	1.33E-15
GRPEL2-A	FZD4	0.369199	3.29E-10
GRPEL2-A	CXCL12	0.339053	9.64E-09
GS1-600G	CDH2	0.354115	1.86E-09
GYG2-AS1	ZEB1	0.388999	2.94E-11
GYG2-AS1	FZD4	0.313466	1.29E-07
HAND2-A	CXCL12	0.477976	⩽0.0001
HAND2-A	ZEB1	0.634757	⩽0.0001
HAND2-A	FGF2	0.422029	3.57E-13
HAND2-A	FZD4	0.384567	5.12E-11
HAND2-A	TWIST1	0.380953	8.00E-11
HAND2-A	BIRC5	-0.37963	9.40E-11
HAND2-A	TWIST2	0.368336	3.64E-10
HAR1B	SNAI1	0.324743	4.24E-08
HCG11	CXCL12	0.423202	3.03E-13
HCG11	FGF2	0.413246	1.21E-12
HCG11	EGF	0.36059	8.96E-10
HCG11	ZEB1	0.34906	3.27E-09
HCG11	SNAI1	-0.34235	6.78E-09
HCG11	BIRC5	-0.33213	1.99E-08
HCG11	TGFB1	-0.31366	1.27E-07
HCG14	ZEB1	0.376786	1.33E-10
HCG14	FZD4	0.375712	1.51E-10
HCG14	TWIST2	0.331018	2.24E-08
HCG14	TWIST1	0.310809	1.67E-07
HCG18	CDH2	0.37002	2.99E-10
HCG21	TWIST1	0.375355	1.58E-10

HCG22	CXCL12	0.389638	2.71E-11
HCG22	BIRC5	-0.30178	3.92E-07
HCG24	CDH2	0.452102	4.00E-15
HCG9	ZEB1	0.371454	2.52E-10
HCP5B	CXCL12	0.36651	4.51E-10
HCP5B	BIRC5	-0.33596	1.34E-08
HDAC11-/	CXCL12	-0.30511	2.87E-07
HEIH	CXCL12	0.365071	5.34E-10
HEIH	BIRC5	-0.3297	2.56E-08
HEXA-AS1	EGF	0.391467	2.15E-11
HEXA-AS1	BIRC5	-0.39032	2.48E-11
HHIP-AS1	FGF2	0.329706	2.56E-08
HHIP-AS1	TWIST1	0.31308	1.34E-07
HID1-AS1	CXCL12	0.812266	≤0.0001
HID1-AS1	BIRC5	-0.58302	≤0.0001
HID1-AS1	FZD4	0.815532	≤0.0001
HID1-AS1	FGF2	0.734973	≤0.0001
HID1-AS1	TWIST2	0.682859	≤0.0001
HID1-AS1	ZEB1	0.768743	≤0.0001
HID1-AS1	TWIST1	0.507574	≤0.0001
HID1-AS1	CXCR4	-0.33831	1.04E-08
HIF1A-AS:	HIF1A	0.52272	≤0.0001
HLA-F-AS	BIRC5	-0.34025	8.49E-09
HLA-F-AS	CXCL12	0.32136	5.96E-08
HLX-AS1	CXCL12	0.601625	≤0.0001
HLX-AS1	FZD4	0.547587	≤0.0001
HLX-AS1	FGF2	0.534574	≤0.0001
HLX-AS1	ZEB1	0.590469	≤0.0001
HLX-AS1	TWIST2	0.459418	1.33E-15
HLX-AS1	BIRC5	-0.45669	2.00E-15
HORMAD:	FZD4	0.636975	≤0.0001
HORMAD:	ZEB1	0.513917	≤0.0001
HORMAD:	TWIST2	0.447622	8.44E-15
HORMAD:	FGF2	0.44543	1.15E-14
HORMAD:	CXCL12	0.404749	3.81E-12
HORMAD:	TWIST1	0.301007	4.21E-07
HOTAIR	CXCL12	-0.34317	6.21E-09
HOTAIR	FGF2	-0.32921	2.69E-08
HOXA-AS:	BIRC5	-0.37747	1.22E-10
HOXA-AS:	CXCL12	0.338062	1.07E-08
HOXA-AS:	EGF	0.328029	3.04E-08
HOXA-AS:	EGF	0.321148	6.09E-08
HOXA-AS:	BIRC5	-0.31065	1.70E-07
HOXB-AS:	CXCL12	0.580399	≤0.0001
HOXB-AS:	BIRC5	-0.47305	≤0.0001
HOXB-AS:	FZD4	0.899671	≤0.0001
HOXB-AS:	FGF2	0.709274	≤0.0001
HOXB-AS:	TWIST2	0.848445	≤0.0001
HOXB-AS:	ZEB1	0.737311	≤0.0001
HOXB-AS:	TWIST1	0.584469	≤0.0001
HOXB-AS:	CXCR4	-0.31956	7.12E-08
HOXB-AS:	ZEB1	0.428942	1.33E-13
HOXC13-/	FGF2	-0.3226	5.26E-08
HOXC-AS:	HIF1A	-0.339	9.70E-09
HOXC-AS:	TWIST2	0.325412	3.97E-08
HOXC-AS:	BIRC5	-0.32445	4.37E-08
HOXC-AS:	TWIST1	0.318018	8.30E-08
HOXC-AS:	FZD4	0.300371	4.46E-07

HOXC-AS2	CDH2	0.348111	3.63E-09
HOXC-AS2	CXCL12	-0.31569	1.04E-07
HOXC-AS3	BIRC5	0.402875	4.89E-12
HOXC-AS3	CXCL12	-0.39737	1.01E-11
HOXC-AS3	CDH2	0.378606	1.07E-10
HOXC-AS3	FGF2	-0.34107	7.78E-09
HOXC-AS3	ZEB1	-0.31028	1.76E-07
HOXC-AS3	TWIST2	-0.30413	3.15E-07
HOXD-AS	CXCL12	0.606003	≤0.0001
HOXD-AS	BIRC5	-0.51225	≤0.0001
HOXD-AS	FZD4	0.611917	≤0.0001
HOXD-AS	FGF2	0.477685	≤0.0001
HOXD-AS	TWIST2	0.621953	≤0.0001
HOXD-AS	ZEB1	0.607055	≤0.0001
HOXD-AS	TWIST1	0.443722	1.51E-14
HPN-AS1	CXCL12	-0.44776	7.99E-15
HPN-AS1	ZEB1	-0.43824	3.42E-14
HPN-AS1	BIRC5	0.409967	1.89E-12
HPN-AS1	FZD4	-0.40694	2.85E-12
HPN-AS1	TWIST2	-0.36466	5.60E-10
HS1BP3-IT	ZEB1	0.52873	≤0.0001
HS1BP3-IT	FZD4	0.411344	1.57E-12
HS1BP3-IT	FGF2	0.340789	8.02E-09
HS1BP3-IT	BIRC5	-0.33815	1.06E-08
HS1BP3-IT	TWIST2	0.31664	9.50E-08
HS1BP3-IT	CXCL12	0.316209	9.91E-08
HS6ST2-A	CDH2	0.317162	9.03E-08
HSD52	ZEB1	0.541907	≤0.0001
HSD52	TWIST2	0.443476	1.55E-14
HSD52	FZD4	0.438387	3.35E-14
HSD52	FGF2	0.432047	8.53E-14
HSD52	CXCL12	0.431621	9.06E-14
HTR2A-AS	CDH2	0.352685	2.19E-09
HUNK-AS	HIF1A	0.464152	6.66E-16
HYI-AS1	TGFB1	0.512322	≤0.0001
IGF2-AS	TGFB1	0.357206	1.32E-09
IGF2BP2-A	CDH2	0.355361	1.62E-09
IGFBP7-AS	PDGFB	0.320828	6.28E-08
IQCH-AS1	EGF	0.519401	≤0.0001
IQCH-AS1	TGFB1	-0.31943	7.22E-08
IQCH-AS1	SNAI1	-0.31188	1.51E-07
ITGA9-AS	EGF	0.584135	≤0.0001
ITGA9-AS	BIRC5	-0.44686	9.33E-15
ITGA9-AS	CXCL12	0.391827	2.05E-11
ITGA9-AS	SNAI1	-0.35182	2.41E-09
ITGB2-AS1	TGFB1	0.352338	2.27E-09
JAZF1-AS1	ZEB1	0.511914	≤0.0001
JAZF1-AS1	CXCL12	0.451702	4.44E-15
JAZF1-AS1	BIRC5	-0.37795	1.15E-10
JAZF1-AS1	TWIST2	0.348249	3.57E-09
JAZF1-AS1	FZD4	0.325448	3.95E-08
JAZF1-AS1	TWIST1	0.301256	4.11E-07
JAZF1-AS1	FGF2	0.300153	4.55E-07
KANSL1-A	BIRC5	-0.30212	3.79E-07
KCNAB1-A	EGF	0.530723	≤0.0001
KCNAB1-A	TGFB1	0.411069	1.63E-12
KCNAB1-A	TWIST1	0.32559	3.90E-08
KCNIP2-A	FZD4	0.762029	≤0.0001

KCNIP2-A	FGF2	0.59146	≤0.0001
KCNIP2-A	TWIST2	0.552198	≤0.0001
KCNIP2-A	ZEB1	0.631453	≤0.0001
KCNIP2-A	CXCL12	0.398121	9.12E-12
KCNIP2-A	TWIST1	0.380318	8.64E-11
KCNIP2-A	BIRC5	-0.31963	7.07E-08
KCNJ2-AS	CXCL12	0.345573	4.79E-09
KCNJ2-AS	EGF	0.312798	1.38E-07
KCNMA1-	CDH2	0.341726	7.25E-09
KCNMA1-	ZEB1	0.303777	3.25E-07
KCNQ1-A	ZEB1	0.486815	≤0.0001
KCNQ1-A	CXCL12	0.349903	2.98E-09
KCNQ1-A	FZD4	0.341777	7.21E-09
KCNQ1-A	BIRC5	-0.31609	1.00E-07
KIAA0087	CXCL12	0.565871	≤0.0001
KIAA0087	BIRC5	-0.48041	≤0.0001
KIAA0087	FZD4	0.479907	≤0.0001
KIAA0087	FGF2	0.475355	≤0.0001
KIAA0087	ZEB1	0.548623	≤0.0001
KIAA0087	TWIST2	0.350095	2.92E-09
KIF9-AS1	BIRC5	-0.31471	1.15E-07
KIRREL3-A	BIRC5	0.33588	1.35E-08
KIRREL3-A	CXCL12	-0.31933	7.29E-08
KIRREL3-A	CDH2	0.602907	≤0.0001
KIZ-AS1	ZEB1	0.315548	1.06E-07
KIZ-AS1	CDH2	0.302079	3.81E-07
KLF3-AS1	EGF	0.495591	≤0.0001
KLF3-AS1	BIRC5	-0.41993	4.80E-13
KLF3-AS1	CXCL12	0.391534	2.13E-11
KLF3-AS1	ZEB1	0.387863	3.39E-11
KLF3-AS1	SNAI1	-0.34745	3.90E-09
KLF3-AS1	FGF2	0.308513	2.08E-07
KMT2E-AS	FGF2	-0.34505	5.07E-09
KMT2E-AS	CXCL12	-0.32911	2.72E-08
KMT2E-AS	ZEB1	-0.32382	4.66E-08
KMT2E-AS	FZD4	-0.30668	2.48E-07
KRTAP5-A	FGF2	-0.32485	4.20E-08
KRTAP5-A	HIF1A	0.321008	6.17E-08
KTN1-AS1	EGF	0.548699	≤0.0001
KTN1-AS1	BIRC5	-0.41612	8.16E-13
KTN1-AS1	CXCL12	0.394312	1.49E-11
L3MBTL4-	CDH2	0.320754	6.33E-08
LAMP5-AS	CDH2	0.389998	2.59E-11
LAMTOR5	CDH2	0.323572	4.78E-08
LANCL1-A	ZEB1	0.399984	7.15E-12
LARS2-AS	CDH2	0.319105	7.45E-08
LDLRAD4-	ZEB1	0.319921	6.87E-08
LEF1-AS1	TGFB1	0.693009	≤0.0001
LENG8-AS	EGF	0.326736	3.47E-08
LIFR-AS1	BIRC5	-0.48752	≤0.0001
LIFR-AS1	EGF	0.624115	≤0.0001
LIFR-AS1	CXCL12	0.442895	1.69E-14
LIFR-AS1	SNAI1	-0.38127	7.69E-11
LIFR-AS1	FGF2	0.308002	2.19E-07
LIMD1-AS	EGF	0.448829	6.88E-15
LINC0011	CXCL12	-0.39073	2.36E-11
LINC0011	FZD4	-0.3699	3.03E-10
LINC0011	FGF2	-0.35591	1.52E-09

LINC00115	TWIST2	-0.30781	2.23E-07
LINC00173	EGF	0.464531	4.44E-16
LINC00174	HIF1A	0.314525	1.17E-07
LINC00189	TGFB1	0.377308	1.25E-10
LINC00205	FZD4	-0.38053	8.42E-11
LINC00205	TWIST2	-0.37126	2.58E-10
LINC00205	ZEB1	-0.36455	5.68E-10
LINC00205	CXCL12	-0.3496	3.08E-09
LINC00210	CDH2	0.352097	2.34E-09
LINC00211	CXCL12	0.353102	2.09E-09
LINC00211	BIRC5	-0.30787	2.21E-07
LINC00229	HIF1A	0.300065	4.59E-07
LINC00239	FZD4	-0.35751	1.27E-09
LINC00239	ZEB1	-0.34127	7.61E-09
LINC00239	FGF2	-0.31039	1.74E-07
LINC00240	FZD4	-0.32583	3.80E-08
LINC00240	ZEB1	-0.31376	1.26E-07
LINC00242	CXCL12	0.399339	7.78E-12
LINC00242	TWIST2	0.333243	1.78E-08
LINC00265	BIRC5	-0.35323	2.06E-09
LINC00266	CXCL12	0.319185	7.39E-08
LINC00266	BIRC5	-0.3187	7.76E-08
LINC00266	EGF	0.317192	9.00E-08
LINC00271	BIRC5	-0.48226	≤0.0001
LINC00271	EGF	0.465762	4.44E-16
LINC00271	CXCL12	0.431503	9.21E-14
LINC00305	CDH2	0.576785	≤0.0001
LINC00310	ZEB1	0.427626	1.61E-13
LINC00314	CDH2	0.331754	2.07E-08
LINC00314	ZEB1	0.30203	3.83E-07
LINC00324	BIRC5	-0.43644	4.46E-14
LINC00324	EGF	0.367501	4.02E-10
LINC00324	CXCL12	0.344631	5.30E-09
LINC00332	ZEB1	0.451302	4.66E-15
LINC00332	FZD4	0.358397	1.15E-09
LINC00332	TWIST2	0.336028	1.33E-08
LINC00336	HIF1A	0.334081	1.63E-08
LINC00337	CXCL12	-0.47238	≤0.0001
LINC00337	ZEB1	-0.42006	4.72E-13
LINC00337	BIRC5	0.400043	7.10E-12
LINC00337	FZD4	-0.37515	1.62E-10
LINC00337	TWIST2	-0.33481	1.51E-08
LINC00354	CDH2	0.34131	7.58E-09
LINC00368	ZEB1	0.407526	2.63E-12
LINC00377	ZEB1	0.369505	3.18E-10
LINC00398	CXCL12	0.479619	≤0.0001
LINC00398	BIRC5	-0.47696	≤0.0001
LINC00398	FZD4	0.589355	≤0.0001
LINC00398	FGF2	0.522159	≤0.0001
LINC00398	ZEB1	0.550092	≤0.0001
LINC00398	TWIST2	0.424096	2.67E-13
LINC00402	CXCR4	0.333418	1.74E-08
LINC00404	CDH2	0.560611	≤0.0001
LINC00421	CXCR4	0.362387	7.29E-10
LINC00426	CXCR4	0.382973	6.23E-11
LINC00426	TGFB1	0.307184	2.36E-07
LINC00428	TGFB1	0.305643	2.73E-07
LINC00431	CDH2	0.325343	3.99E-08

LINC00440	CDH2	0.325832	3.80E-08
LINC00445	EGF	0.463022	8.88E-16
LINC00454	PDGFB	0.316162	9.95E-08
LINC00461	BIRC5	0.362689	7.04E-10
LINC00461	CXCL12	-0.34895	3.31E-09
LINC00466	BIRC5	0.380696	8.25E-11
LINC00472	EGF	0.536283	≤0.0001
LINC00472	BIRC5	-0.40749	2.64E-12
LINC00472	CXCL12	0.391609	2.11E-11
LINC00472	SNAI1	-0.30289	3.53E-07
LINC00474	HIF1A	0.355184	1.65E-09
LINC00484	ZEB1	0.50955	≤0.0001
LINC00484	FZD4	0.385261	4.69E-11
LINC00484	TWIST2	0.331717	2.08E-08
LINC00499	CDH2	0.336686	1.24E-08
LINC00500	CDH2	0.312308	1.45E-07
LINC00504	BIRC5	-0.48543	≤0.0001
LINC00504	CXCL12	0.44142	2.13E-14
LINC00504	EGF	0.426481	1.90E-13
LINC00506	EGF	0.323871	4.63E-08
LINC00507	HIF1A	0.314736	1.14E-07
LINC00511	CXCL12	-0.4524	4.00E-15
LINC00511	BIRC5	0.444617	1.31E-14
LINC00511	FZD4	-0.35444	1.80E-09
LINC00511	ZEB1	-0.3172	8.99E-08
LINC00511	TWIST2	-0.31375	1.26E-07
LINC00517	CXCL12	0.372898	2.12E-10
LINC00517	BIRC5	-0.33323	1.78E-08
LINC00517	ZEB1	0.300833	4.28E-07
LINC00526	SNAI1	-0.31866	7.78E-08
LINC00528	TGFB1	0.373625	1.95E-10
LINC00528	CXCR4	0.353522	1.99E-09
LINC00535	ZEB1	0.32995	2.50E-08
LINC00538	CDH2	0.364773	5.53E-10
LINC00552	CXCR4	0.388505	3.12E-11
LINC00561	ZEB1	0.31189	1.51E-07
LINC00562	BIRC5	-0.35923	1.05E-09
LINC00562	ZEB1	0.35827	1.17E-09
LINC00562	CXCL12	0.349103	3.25E-09
LINC00565	PDGFB	0.360174	9.39E-10
LINC00570	FGF2	0.369249	3.27E-10
LINC00571	ZEB1	0.318407	7.99E-08
LINC00574	CXCL12	0.503208	≤0.0001
LINC00574	BIRC5	-0.4008	6.43E-12
LINC00574	FGF2	0.367158	4.18E-10
LINC00574	EGF	0.335637	1.38E-08
LINC00574	ZEB1	0.330189	2.44E-08
LINC00574	FZD4	0.322483	5.33E-08
LINC00581	CDH2	0.383309	5.98E-11
LINC00592	ZEB1	-0.33133	2.17E-08
LINC00592	FZD4	-0.32318	4.96E-08
LINC00592	FGF2	-0.30065	4.35E-07
LINC00598	ZEB1	0.569262	≤0.0001
LINC00598	BIRC5	-0.35196	2.37E-09
LINC00598	TWIST1	0.319045	7.50E-08
LINC00598	FZD4	0.300431	4.44E-07
LINC00599	CDH2	0.418666	5.73E-13
LINC00599	TWIST1	0.323032	5.04E-08

LINC00603	CXCL12	0.383913	5.55E-11
LINC00607	TGFB1	0.392789	1.81E-11
LINC00624	HIF1A	0.39578	1.24E-11
LINC00626	TGFB1	0.489218	≤0.0001
LINC00632	FZD4	0.473071	≤0.0001
LINC00632	TWIST1	0.485613	≤0.0001
LINC00632	TWIST2	0.457768	1.78E-15
LINC00632	ZEB1	0.454165	3.11E-15
LINC00632	FGF2	0.328502	2.90E-08
LINC00635	FZD4	0.464657	4.44E-16
LINC00635	TWIST2	0.406548	3.00E-12
LINC00635	ZEB1	0.388389	3.17E-11
LINC00635	FGF2	0.338323	1.04E-08
LINC00635	CXCL12	0.329053	2.74E-08
LINC00636	CXCL12	0.4771	≤0.0001
LINC00640	EGF	0.675018	≤0.0001
LINC00640	BIRC5	-0.41363	1.15E-12
LINC00640	CXCL12	0.368037	3.77E-10
LINC00641	CXCL12	0.628127	≤0.0001
LINC00641	BIRC5	-0.58649	≤0.0001
LINC00641	FZD4	0.621626	≤0.0001
LINC00641	FGF2	0.629981	≤0.0001
LINC00641	ZEB1	0.679503	≤0.0001
LINC00641	TWIST2	0.470135	2.22E-16
LINC00641	EGF	0.358221	1.17E-09
LINC00641	CXCR4	-0.32467	4.28E-08
LINC00641	SNAI1	-0.32464	4.29E-08
LINC00641	TGFB1	-0.30277	3.57E-07
LINC00643	FZD4	0.466404	4.44E-16
LINC00643	TWIST2	0.45042	5.33E-15
LINC00643	FGF2	0.42829	1.47E-13
LINC00643	ZEB1	0.389224	2.85E-11
LINC00645	ZEB1	0.496862	≤0.0001
LINC00654	ZEB1	0.421535	3.83E-13
LINC00654	FGF2	0.34801	3.67E-09
LINC00654	FZD4	0.328042	3.04E-08
LINC00665	CXCL12	-0.42912	1.30E-13
LINC00665	BIRC5	0.414441	1.03E-12
LINC00665	ZEB1	-0.4046	3.89E-12
LINC00665	FZD4	-0.39864	8.53E-12
LINC00665	TWIST2	-0.38503	4.83E-11
LINC00665	FGF2	-0.37204	2.35E-10
LINC00667	CXCL12	0.618861	≤0.0001
LINC00667	FZD4	0.617302	≤0.0001
LINC00667	FGF2	0.654524	≤0.0001
LINC00667	TWIST2	0.477844	≤0.0001
LINC00667	ZEB1	0.563172	≤0.0001
LINC00667	BIRC5	-0.44462	1.31E-14
LINC00667	TWIST1	0.341754	7.23E-09
LINC00667	SNAI1	-0.30445	3.05E-07
LINC00670	ZEB1	0.427896	1.55E-13
LINC00670	CXCL12	0.362412	7.26E-10
LINC00670	FZD4	0.358503	1.14E-09
LINC00670	FGF2	0.35303	2.11E-09
LINC00671	CXCL12	0.626688	≤0.0001
LINC00671	BIRC5	-0.47503	≤0.0001
LINC00671	FZD4	0.813189	≤0.0001
LINC00671	FGF2	0.6188	≤0.0001

LINC00671	TWIST2	0.687138	≤0.0001
LINC00671	ZEB1	0.644975	≤0.0001
LINC00671	TWIST1	0.514211	≤0.0001
LINC00671	CXCR4	-0.30707	2.39E-07
LINC00680	BIRC5	0.437014	4.11E-14
LINC00680	CXCL12	-0.41125	1.59E-12
LINC00680	FZD4	-0.37337	2.01E-10
LINC00680	FGF2	-0.37226	2.29E-10
LINC00680	TWIST2	-0.33425	1.60E-08
LINC00680	CXCR4	0.316748	9.40E-08
LINC00681	CDH2	0.495341	≤0.0001
LINC00702	CXCL12	0.576992	≤0.0001
LINC00702	FZD4	0.581698	≤0.0001
LINC00702	FGF2	0.501057	≤0.0001
LINC00702	TWIST2	0.479373	≤0.0001
LINC00702	ZEB1	0.672844	≤0.0001
LINC00702	BIRC5	-0.41811	6.19E-13
LINC00702	TWIST1	0.305576	2.75E-07
LINC00710	ZEB1	0.457317	1.78E-15
LINC00839	CXCL12	-0.30541	2.79E-07
LINC00840	CXCL12	0.537381	≤0.0001
LINC00840	ZEB1	0.543189	≤0.0001
LINC00840	FZD4	0.450879	4.88E-15
LINC00840	FGF2	0.404203	4.10E-12
LINC00840	BIRC5	-0.38989	2.62E-11
LINC00840	TWIST2	0.369386	3.22E-10
LINC00843	ZEB1	0.311007	1.64E-07
LINC00844	BIRC5	-0.43623	4.62E-14
LINC00844	CXCL12	0.423499	2.90E-13
LINC00844	EGF	0.356956	1.35E-09
LINC00853	ZEB1	-0.30206	3.82E-07
LINC00861	CXCR4	0.34611	4.51E-09
LINC00862	HIF1A	0.372688	2.18E-10
LINC00863	BIRC5	-0.30499	2.90E-07
LINC00866	EGF	0.440309	2.51E-14
LINC00877	TGFB1	0.413452	1.18E-12
LINC00882	CXCL12	0.362352	7.32E-10
LINC00882	ZEB1	0.349527	3.10E-09
LINC00882	BIRC5	-0.33886	9.84E-09
LINC00885	ZEB1	-0.3702	2.92E-10
LINC00885	FZD4	-0.32411	4.53E-08
LINC00886	BIRC5	-0.52361	≤0.0001
LINC00886	CXCL12	0.4566	2.00E-15
LINC00886	EGF	0.435471	5.15E-14
LINC00886	FGF2	0.38657	3.98E-11
LINC00886	ZEB1	0.318217	8.14E-08
LINC00886	FZD4	0.315584	1.05E-07
LINC00891	CXCL12	0.528031	≤0.0001
LINC00891	FZD4	0.5713	≤0.0001
LINC00891	FGF2	0.495307	≤0.0001
LINC00891	TWIST2	0.521341	≤0.0001
LINC00891	ZEB1	0.579414	≤0.0001
LINC00891	BIRC5	-0.38113	7.82E-11
LINC00892	TGFB1	0.351507	2.49E-09
LINC00892	CXCR4	0.334539	1.55E-08
LINC00893	EGF	0.465543	4.44E-16
LINC00893	BIRC5	-0.43482	5.68E-14
LINC00893	CXCL12	0.351842	2.40E-09

LINC00893	ZEB1	0.306936	2.42E-07
LINC00894	ZEB1	0.300687	4.33E-07
LINC00899	EGF	0.339271	9.42E-09
LINC00901	CDH2	0.352384	2.26E-09
LINC00903	CDH2	0.352306	2.28E-09
LINC00906	FZD4	0.497029	≤0.0001
LINC00906	ZEB1	0.480718	≤0.0001
LINC00906	FGF2	0.455041	2.66E-15
LINC00906	CXCL12	0.443809	1.49E-14
LINC00906	TWIST2	0.37546	1.56E-10
LINC00906	TWIST1	0.352908	2.13E-09
LINC00906	BIRC5	-0.32494	4.16E-08
LINC00908	CXCL12	0.645618	≤0.0001
LINC00908	BIRC5	-0.5165	≤0.0001
LINC00908	EGF	0.426309	1.95E-13
LINC00908	ZEB1	0.31832	8.05E-08
LINC00908	FGF2	0.308835	2.02E-07
LINC00909	EGF	0.344005	5.67E-09
LINC0091(	BIRC5	-0.4297	1.20E-13
LINC0091(	CXCL12	0.34236	6.77E-09
LINC0091(	EGF	0.3187	7.76E-08
LINC0091(	FGF2	0.300443	4.43E-07
LINC0092(	EGF	0.457738	1.78E-15
LINC0092(	BIRC5	-0.37174	2.44E-10
LINC00921	CXCL12	0.561086	≤0.0001
LINC00921	BIRC5	-0.58279	≤0.0001
LINC00921	FZD4	0.492493	≤0.0001
LINC00921	FGF2	0.497591	≤0.0001
LINC00921	ZEB1	0.457138	1.78E-15
LINC00921	TWIST2	0.40381	4.32E-12
LINC00921	HIF1A	-0.30472	2.98E-07
LINC00924	CXCL12	0.630872	≤0.0001
LINC00924	ZEB1	0.509562	≤0.0001
LINC00924	BIRC5	-0.43164	9.02E-14
LINC00924	FZD4	0.427817	1.57E-13
LINC00924	FGF2	0.423628	2.85E-13
LINC00924	TWIST2	0.339761	8.94E-09
LINC00924	TWIST1	0.329936	2.50E-08
LINC00928	CDH2	0.317863	8.42E-08
LINC00944	BIRC5	0.326093	3.70E-08
LINC00957	HIF1A	-0.31022	1.77E-07
LINC00968	FZD4	0.510782	≤0.0001
LINC00968	ZEB1	0.510168	≤0.0001
LINC00968	FGF2	0.440816	2.33E-14
LINC00968	TWIST2	0.408871	2.19E-12
LINC00968	CXCL12	0.374609	1.73E-10
LINC00974	TWIST2	0.397158	1.03E-11
LINC00974	FZD4	0.394561	1.45E-11
LINC00982	CXCL12	0.492112	≤0.0001
LINC00982	FZD4	0.543044	≤0.0001
LINC00982	TWIST2	0.510414	≤0.0001
LINC00982	ZEB1	0.459665	1.33E-15
LINC00982	FGF2	0.434785	5.71E-14
LINC00982	BIRC5	-0.40566	3.38E-12
LINC00982	TWIST1	0.3247	4.26E-08
LINC00989	CXCL12	0.62656	≤0.0001
LINC00989	BIRC5	-0.51663	≤0.0001
LINC00989	FZD4	0.672576	≤0.0001

LINC0098[illegible]	FGF2	0.577935	≤0.0001
LINC0098[illegible]	TWIST2	0.588301	≤0.0001
LINC0098[illegible]	ZEB1	0.682394	≤0.0001
LINC0098[illegible]	TWIST1	0.455433	2.44E-15
LINC0099[illegible]	BIRC5	-0.41454	1.01E-12
LINC0099[illegible]	EGF	0.382535	6.58E-11
LINC0099[illegible]	CXCL12	0.366962	4.28E-10
LINC0099[illegible]	ZEB1	0.379612	9.42E-11
LINC0099[illegible]	CXCL12	0.425877	2.07E-13
LINC0099[illegible]	BIRC5	-0.34026	8.48E-09
LINC0100[illegible]	CXCL12	-0.45422	3.11E-15
LINC0100[illegible]	ZEB1	-0.41431	1.05E-12
LINC0100[illegible]	BIRC5	0.373278	2.03E-10
LINC0100[illegible]	TWIST2	-0.36523	5.24E-10
LINC0100[illegible]	FZD4	-0.34436	5.46E-09
LINC0100[illegible]	FGF2	-0.33885	9.85E-09
LINC0100[illegible]	TWIST1	-0.31409	1.22E-07
LINC0101[illegible]	CXCL12	-0.33239	1.94E-08
LINC0101[illegible]	CDH2	0.329719	2.56E-08
LINC0101[illegible]	FZD4	-0.32925	2.68E-08
LINC0101[illegible]	TWIST2	-0.31509	1.11E-07
LINC0101[illegible]	TGFB1	0.421843	3.67E-13
LINC0101[illegible]	ZEB1	0.302251	3.75E-07
LINC0101[illegible]	HIF1A	0.308371	2.11E-07
LINC0102[illegible]	TGFB1	0.423137	3.06E-13
LINC0102[illegible]	TWIST1	0.348346	3.54E-09
LINC0102[illegible]	CXCL12	0.340316	8.43E-09
LINC0103[illegible]	CDH2	0.321848	5.68E-08
LINC0103[illegible]	CDH2	0.332434	1.93E-08
LINC0104[illegible]	ZEB1	0.329233	2.69E-08
LINC0104[illegible]	FZD4	0.31981	6.95E-08
LINC0104[illegible]	FZD4	0.713667	≤0.0001
LINC0104[illegible]	FGF2	0.554336	≤0.0001
LINC0104[illegible]	TWIST2	0.575637	≤0.0001
LINC0104[illegible]	ZEB1	0.563196	≤0.0001
LINC0104[illegible]	CXCL12	0.373452	1.99E-10
LINC0104[illegible]	TWIST1	0.312118	1.47E-07
LINC0105[illegible]	TGFB1	0.413935	1.10E-12
LINC0105[illegible]	SNAI1	0.314685	1.15E-07
LINC0105[illegible]	CXCL12	0.410318	1.80E-12
LINC0105[illegible]	CXCL12	0.573849	≤0.0001
LINC0105[illegible]	BIRC5	-0.53955	≤0.0001
LINC0105[illegible]	FZD4	0.618072	≤0.0001
LINC0105[illegible]	FGF2	0.623506	≤0.0001
LINC0105[illegible]	ZEB1	0.546306	≤0.0001
LINC0105[illegible]	TWIST2	0.460179	1.11E-15
LINC0106[illegible]	EGF	0.349961	2.96E-09
LINC0106[illegible]	CXCL12	-0.35726	1.31E-09
LINC0106[illegible]	BIRC5	0.3428	6.46E-09
LINC0106[illegible]	ZEB1	-0.33307	1.81E-08
LINC0106[illegible]	FGF2	-0.31911	7.45E-08
LINC0107[illegible]	CXCL12	0.614474	≤0.0001
LINC0107[illegible]	FZD4	0.836583	≤0.0001
LINC0107[illegible]	FGF2	0.69566	≤0.0001
LINC0107[illegible]	TWIST2	0.700154	≤0.0001
LINC0107[illegible]	ZEB1	0.666748	≤0.0001
LINC0107[illegible]	BIRC5	-0.42581	2.09E-13
LINC0107[illegible]	TWIST1	0.39835	8.85E-12

LINC01080	HIF1A	0.314581	1.16E-07
LINC01082	FZD4	0.664629	≤0.0001
LINC01082	TWIST2	0.607364	≤0.0001
LINC01082	ZEB1	0.514921	≤0.0001
LINC01082	FGF2	0.455622	2.44E-15
LINC01082	CXCL12	0.381683	7.31E-11
LINC01085	CXCL12	0.438592	3.24E-14
LINC01085	BIRC5	-0.39173	2.08E-11
LINC01085	EGF	0.315212	1.09E-07
LINC01091	ZEB1	0.450995	4.88E-15
LINC01094	HIF1A	0.409085	2.13E-12
LINC01094	CXCL12	-0.352	2.36E-09
LINC01094	TWIST2	-0.35053	2.78E-09
LINC01094	FZD4	-0.34809	3.64E-09
LINC01097	CDH2	0.370822	2.72E-10
LINC01108	TGFB1	0.331634	2.10E-08
LINC01109	CDH2	0.400779	6.44E-12
LINC01122	CDH2	0.321092	6.12E-08
LINC01123	SNAI1	0.313504	1.29E-07
LINC01128	CXCL12	0.554637	≤0.0001
LINC01128	BIRC5	-0.47354	≤0.0001
LINC01128	FZD4	0.786445	≤0.0001
LINC01128	FGF2	0.681888	≤0.0001
LINC01128	TWIST2	0.645144	≤0.0001
LINC01128	ZEB1	0.697979	≤0.0001
LINC01128	TWIST1	0.430359	1.09E-13
LINC01132	HIF1A	0.398119	9.12E-12
LINC01135	BIRC5	-0.36017	9.39E-10
LINC01135	ZEB1	0.344872	5.16E-09
LINC01135	CXCL12	0.33361	1.71E-08
LINC01135	FGF2	0.318756	7.71E-08
LINC01135	FZD4	0.301984	3.84E-07
LINC01140	CXCL12	0.514668	≤0.0001
LINC01140	FZD4	0.766118	≤0.0001
LINC01140	FGF2	0.653206	≤0.0001
LINC01140	TWIST2	0.728385	≤0.0001
LINC01140	ZEB1	0.649806	≤0.0001
LINC01140	TWIST1	0.446599	9.77E-15
LINC01140	BIRC5	-0.32667	3.49E-08
LINC01142	TGFB1	0.454347	2.89E-15
LINC01142	TWIST1	0.33278	1.86E-08
LINC01143	CXCL12	-0.36741	4.06E-10
LINC01143	BIRC5	0.318885	7.62E-08
LINC01143	FZD4	-0.30448	3.04E-07
LINC01144	ZEB1	-0.36575	4.94E-10
LINC01144	FZD4	-0.32411	4.52E-08
LINC01150	TGFB1	0.408519	2.30E-12
LINC01152	EGF	0.434509	5.95E-14
LINC01153	CDH2	0.554042	≤0.0001
LINC01179	HIF1A	0.386912	3.82E-11
LINC01184	EGF	0.308617	2.06E-07
LINC01186	FZD4	0.591704	≤0.0001
LINC01186	FGF2	0.475665	≤0.0001
LINC01186	TWIST2	0.521429	≤0.0001
LINC01186	ZEB1	0.455871	2.22E-15
LINC01186	CXCL12	0.39978	7.34E-12
LINC01186	BIRC5	-0.31169	1.54E-07
LINC01197	CXCL12	0.775981	≤0.0001

LINC01197	BIRC5	-0.54032	≤0.0001
LINC01197	FZD4	0.642322	≤0.0001
LINC01197	FGF2	0.582583	≤0.0001
LINC01197	TWIST2	0.529828	≤0.0001
LINC01197	ZEB1	0.705506	≤0.0001
LINC01197	TWIST1	0.410822	1.69E-12
LINC01198	EGF	0.331515	2.13E-08
LINC0120:	CDH2	0.326831	3.44E-08
LINC01206	CXCR4	0.328715	2.83E-08
LINC01214	TGFB1	0.3019	3.87E-07
LINC01215	CXCR4	0.354594	1.77E-09
LINC0122(	CXCL12	0.521503	≤0.0001
LINC0122(	FZD4	0.619009	≤0.0001
LINC0122(	FGF2	0.536249	≤0.0001
LINC0122(	TWIST2	0.605315	≤0.0001
LINC0122(	ZEB1	0.554785	≤0.0001
LINC0122(	BIRC5	-0.44478	1.29E-14
LINC0122(	TWIST1	0.434264	6.15E-14
LINC0122(	CXCR4	-0.30563	2.73E-07
LINC01221	TGFB1	0.384818	4.96E-11
LINC0123(	FZD4	0.510451	≤0.0001
LINC0123(	FGF2	0.519426	≤0.0001
LINC0123(	TWIST2	0.580896	≤0.0001
LINC0123(	TWIST1	0.475824	≤0.0001
LINC0123(	ZEB1	0.459767	1.33E-15
LINC0123(	CXCL12	0.317522	8.71E-08
LINC01237	EGF	0.47861	≤0.0001
LINC01237	BIRC5	-0.31827	8.09E-08
LINC01239	FZD4	0.612897	≤0.0001
LINC01239	FGF2	0.538032	≤0.0001
LINC01239	TWIST2	0.525084	≤0.0001
LINC01239	ZEB1	0.543357	≤0.0001
LINC01239	TWIST1	0.386224	4.16E-11
LINC01239	CXCL12	0.320539	6.47E-08
LINC01241	CDH2	0.308518	2.08E-07
LINC01248	BIRC5	0.376697	1.34E-10
LINC01262	FZD4	0.441638	2.04E-14
LINC01262	TWIST2	0.351226	2.57E-09
LINC01266	EGF	0.562871	≤0.0001
LINC01266	BIRC5	-0.42679	1.82E-13
LINC01266	CXCL12	0.416874	7.35E-13
LINC01267	BIRC5	-0.42289	3.17E-13
LINC01267	CXCL12	0.407223	2.74E-12
LINC01267	EGF	0.370825	2.72E-10
LINC01278	EGF	0.330162	2.44E-08
LINC01281	CXCR4	0.403209	4.68E-12
LINC01281	TGFB1	0.327257	3.29E-08
LINC01282	TGFB1	0.429363	1.26E-13
LINC01305	TWIST1	0.358405	1.15E-09
LINC01305	TGFB1	0.316126	9.99E-08
LINC01311	BIRC5	0.439658	2.78E-14
LINC01311	ZEB1	-0.41443	1.03E-12
LINC01311	CXCL12	-0.3885	3.13E-11
LINC01311	FGF2	-0.38218	6.88E-11
LINC01311	FZD4	-0.38183	7.18E-11
LINC01311	TWIST2	-0.31293	1.36E-07
LINC01315	ZEB1	-0.4176	6.64E-13
LINC01315	BIRC5	0.369273	3.26E-10

LINC01315	CXCL12	-0.35487	1.71E-09
LINC01315	FZD4	-0.35344	2.01E-09
LINC01315	FGF2	-0.32484	4.20E-08
LINC01315	TWIST2	-0.30366	3.29E-07
LINC0133(	CXCL12	0.479829	≤0.0001
LINC0133(	BIRC5	-0.50063	≤0.0001
LINC0133(	EGF	0.590527	≤0.0001
LINC0133(	ZEB1	0.354661	1.75E-09
LINC0133(	FGF2	0.306227	2.58E-07
LINC0134(	EGF	0.476681	≤0.0001
LINC0134(	BIRC5	-0.34402	5.66E-09
LINC0134:	HIF1A	0.388216	3.24E-11
LINC0135:	CXCL12	0.70737	≤0.0001
LINC0135:	BIRC5	-0.49437	≤0.0001
LINC0135:	FZD4	0.571745	≤0.0001
LINC0135:	FGF2	0.537634	≤0.0001
LINC0135:	TWIST2	0.488226	≤0.0001
LINC0135:	ZEB1	0.640947	≤0.0001
LINC0135:	TWIST1	0.345891	4.62E-09
LINC0135‹	ZEB1	0.367997	3.79E-10
LINC0135‹	FZD4	0.360987	8.56E-10
LINC0135‹	CXCL12	0.329027	2.75E-08
LINC0135‹	FGF2	0.306516	2.52E-07
LINC0135‹	TWIST2	0.300689	4.33E-07
LINC0135(	BIRC5	0.307574	2.28E-07
LINC0135(	ZEB1	0.379321	9.76E-11
LINC0135(	BIRC5	-0.37544	1.56E-10
LINC0135(	EGF	0.329337	2.66E-08
LINC0135(	CXCL12	0.311437	1.57E-07
LINC0136(	CXCL12	0.643947	≤0.0001
LINC0136(	FZD4	0.701269	≤0.0001
LINC0136(	FGF2	0.595437	≤0.0001
LINC0136(	TWIST2	0.62184	≤0.0001
LINC0136(	ZEB1	0.661882	≤0.0001
LINC0136(	BIRC5	-0.45406	3.11E-15
LINC0136(	TWIST1	0.440281	2.53E-14
LINC01381	FZD4	0.570123	≤0.0001
LINC01381	ZEB1	0.535382	≤0.0001
LINC01381	TWIST2	0.466973	4.44E-16
LINC01381	FGF2	0.434167	6.24E-14
LINC01381	CXCL12	0.388831	3.00E-11
LINC01381	TWIST1	0.349632	3.07E-09
LINC01381	BIRC5	-0.31009	1.79E-07
LINC0139:	TWIST2	0.34781	3.75E-09
LINC0139:	FZD4	0.346002	4.57E-09
LINC0139(	ZEB1	0.337686	1.11E-08
LINC0139;	TWIST1	0.33953	9.17E-09
LINC0140:	CXCL12	0.529914	≤0.0001
LINC0140:	BIRC5	-0.48525	≤0.0001
LINC0140:	FZD4	0.623664	≤0.0001
LINC0140:	FGF2	0.553884	≤0.0001
LINC0140:	TWIST2	0.474796	≤0.0001
LINC0140:	ZEB1	0.597563	≤0.0001
LINC0141:	CXCL12	0.586087	≤0.0001
LINC0141:	TWIST2	0.492873	≤0.0001
LINC0141:	ZEB1	0.468846	4.44E-16
LINC0141:	BIRC5	-0.45052	5.33E-15
LINC0141:	FGF2	0.436479	4.44E-14

LINC01412	FZD4	0.417538	6.70E-13
LINC01412	TWIST1	0.384532	5.14E-11
LINC01415	ZEB1	0.664657	≤0.0001
LINC01415	FZD4	0.468232	4.44E-16
LINC01415	TWIST2	0.399659	7.46E-12
LINC01415	FGF2	0.39857	8.60E-12
LINC01415	TWIST1	0.379056	1.01E-10
LINC01415	CXCL12	0.327515	3.20E-08
LINC01415	BIRC5	-0.30123	4.12E-07
LINC01422	ZEB1	0.382994	6.22E-11
LINC01422	BIRC5	-0.35483	1.72E-09
LINC01423	TGFB1	0.382109	6.94E-11
LINC01426	TGFB1	0.361659	7.92E-10
LINC01435	CDH2	0.319802	6.96E-08
LINC01440	ZEB1	0.311373	1.58E-07
LINC01440	FZD4	0.304852	2.94E-07
LINC01443	HIF1A	0.317437	8.79E-08
LINC01456	HIF1A	0.373824	1.90E-10
LINC01471	CDH2	0.310717	1.69E-07
LINC01474	CXCL12	0.660744	≤0.0001
LINC01474	BIRC5	-0.51055	≤0.0001
LINC01474	FZD4	0.663412	≤0.0001
LINC01474	FGF2	0.646787	≤0.0001
LINC01474	TWIST2	0.509858	≤0.0001
LINC01474	ZEB1	0.661673	≤0.0001
LINC01474	TWIST1	0.32778	3.12E-08
LINC01478	CDH2	0.303241	3.42E-07
LINC01484	FZD4	0.749905	≤0.0001
LINC01484	FGF2	0.535526	≤0.0001
LINC01484	TWIST2	0.607295	≤0.0001
LINC01484	ZEB1	0.5808	≤0.0001
LINC01484	TWIST1	0.383117	6.12E-11
LINC01484	CXCL12	0.367661	3.95E-10
LINC01485	FZD4	0.757607	≤0.0001
LINC01485	FGF2	0.563176	≤0.0001
LINC01485	TWIST2	0.752436	≤0.0001
LINC01485	ZEB1	0.572858	≤0.0001
LINC01485	TWIST1	0.501342	≤0.0001
LINC01485	CXCL12	0.464076	6.66E-16
LINC01485	BIRC5	-0.38175	7.25E-11
LINC01504	ZEB1	0.393966	1.56E-11
LINC01504	FZD4	0.357076	1.34E-09
LINC01504	TWIST2	0.324983	4.14E-08
LINC01504	CXCL12	0.306852	2.44E-07
LINC01506	TWIST2	0.42835	1.45E-13
LINC01506	CXCL12	0.345504	4.82E-09
LINC01506	ZEB1	0.32627	3.64E-08
LINC01506	TWIST1	0.311599	1.55E-07
LINC01506	FZD4	0.309208	1.95E-07
LINC01508	CXCL12	-0.35563	1.57E-09
LINC01508	BIRC5	0.35138	2.53E-09
LINC01508	ZEB1	-0.3499	2.98E-09
LINC01515	EGF	0.370127	2.95E-10
LINC01523	HIF1A	0.3683	3.66E-10
LINC01524	EGF	0.348361	3.53E-09
LINC01527	TGFB1	0.42623	1.97E-13
LINC01532	CXCL12	0.608818	≤0.0001
LINC01532	BIRC5	-0.38231	6.77E-11

LINC01537	CXCL12	0.784902	≤0.0001
LINC01537	BIRC5	-0.54549	≤0.0001
LINC01537	FZD4	0.837012	≤0.0001
LINC01537	FGF2	0.744134	≤0.0001
LINC01537	TWIST2	0.734829	≤0.0001
LINC01537	ZEB1	0.733182	≤0.0001
LINC01537	TWIST1	0.480257	≤0.0001
LINC01537	CXCR4	-0.36008	9.50E-10
LINC01544	CDH2	0.306724	2.47E-07
LINC01547	FZD4	-0.37041	2.85E-10
LINC01547	ZEB1	-0.35769	1.25E-09
LINC01547	TWIST2	-0.35582	1.54E-09
LINC01547	CXCL12	-0.34789	3.72E-09
LINC01547	TWIST1	-0.30175	3.93E-07
LINC01550	FGF2	0.476382	≤0.0001
LINC01550	BIRC5	-0.45269	3.77E-15
LINC01550	FZD4	0.445908	1.07E-14
LINC01550	CXCL12	0.401876	5.58E-12
LINC01550	ZEB1	0.363273	6.58E-10
LINC01550	TWIST2	0.356013	1.51E-09
LINC01559	HIF1A	0.360535	9.01E-10
LINC01562	EGF	0.343038	6.30E-09
LINC01562	BIRC5	-0.303	3.50E-07
LINC01568	CDH2	0.34516	5.01E-09
LINC01569	TWIST2	-0.32754	3.20E-08
LINC01569	ZEB1	-0.32066	6.39E-08
LINC01569	FZD4	-0.31282	1.38E-07
LINC01572	CDH2	0.333961	1.65E-08
LINC01578	EGF	0.328189	2.99E-08
LINC01579	TGFB1	0.350631	2.75E-09
LINC01582	HIF1A	0.360802	8.74E-10
LINC01583	TGFB1	0.360818	8.73E-10
LINC01586	FZD4	0.649934	≤0.0001
LINC01586	FGF2	0.565172	≤0.0001
LINC01586	TWIST2	0.631722	≤0.0001
LINC01586	ZEB1	0.594306	≤0.0001
LINC01586	TWIST1	0.646725	≤0.0001
LINC01586	CXCL12	0.319484	7.18E-08
LINC01586	BIRC5	-0.31737	8.84E-08
LINC01588	FGF2	-0.36935	3.23E-10
LINC01588	FZD4	-0.35304	2.10E-09
LINC01588	CXCL12	-0.34933	3.17E-09
LINC01588	ZEB1	-0.34635	4.40E-09
LINC01588	TWIST2	-0.30768	2.25E-07
LINC-PINT	ZEB1	0.345282	4.94E-09
LINGO1-A	CDH2	0.335926	1.34E-08
LIPE-AS1	CXCL12	0.485908	≤0.0001
LIPE-AS1	FZD4	0.788465	≤0.0001
LIPE-AS1	FGF2	0.607929	≤0.0001
LIPE-AS1	TWIST2	0.784505	≤0.0001
LIPE-AS1	ZEB1	0.597262	≤0.0001
LIPE-AS1	TWIST1	0.613113	≤0.0001
LIPE-AS1	BIRC5	-0.40106	6.21E-12
LIPE-AS1	HIF1A	-0.32646	3.57E-08
LL22NC03	CXCL12	-0.33711	1.18E-08
LMF1-AS1	EGF	0.421014	4.13E-13
LMO7DN-	CXCL12	0.543711	≤0.0001
LMO7DN-	BIRC5	-0.44995	5.77E-15

LMO7DN-	ZEB1	0.392695	1.84E-11
LMO7DN-	EGF	0.373119	2.07E-10
LMO7DN-	FGF2	0.327651	3.16E-08
LPP-AS2	BIRC5	-0.5092	≤0.0001
LPP-AS2	CXCL12	0.45208	4.22E-15
LPP-AS2	FGF2	0.378655	1.06E-10
LPP-AS2	EGF	0.361743	7.85E-10
LPP-AS2	FZD4	0.31146	1.57E-07
LPP-AS2	SNAI1	-0.30596	2.65E-07
LRP4-AS1	CDH2	0.343968	5.70E-09
LRRC75A-	CDH2	0.426025	2.03E-13
LSINCT5	BIRC5	-0.31884	7.65E-08
LY86-AS1	FZD4	0.411104	1.62E-12
LY86-AS1	ZEB1	0.335947	1.34E-08
LY86-AS1	TWIST2	0.316275	9.85E-08
LY86-AS1	FGF2	0.314666	1.15E-07
MAFA-AS:	TGFB1	0.494629	≤0.0001
MAGI1-AS	CDH2	0.342961	6.35E-09
MAGI1-IT:	CDH2	0.309415	1.91E-07
MAGI2-AS	CDH2	0.353388	2.02E-09
MAGI2-AS	CXCL12	0.799055	≤0.0001
MAGI2-AS	BIRC5	-0.64166	≤0.0001
MAGI2-AS	FZD4	0.812518	≤0.0001
MAGI2-AS	FGF2	0.81452	≤0.0001
MAGI2-AS	TWIST2	0.666475	≤0.0001
MAGI2-AS	ZEB1	0.827094	≤0.0001
MAGI2-AS	TWIST1	0.509936	≤0.0001
MAGI2-AS	CXCR4	-0.36689	4.32E-10
MAN2C1	BIRC5	-0.39956	7.56E-12
MAN2C1	CXCL12	0.348639	3.42E-09
MAN2C1	ZEB1	0.327719	3.14E-08
MAN2C1	FGF2	0.30108	4.18E-07
MAP3K14-	TWIST2	-0.32789	3.08E-08
MAP3K14-	TWIST1	-0.31143	1.58E-07
MAP3K14-	FZD4	-0.30013	4.56E-07
MAPKAPK	ZEB1	-0.39289	1.79E-11
MAST4-AS	BIRC5	-0.44329	1.60E-14
MAST4-AS	CXCL12	0.393083	1.75E-11
MAST4-AS	EGF	0.305653	2.73E-07
MATN1-A	FZD4	-0.30138	4.07E-07
MBNL1-AS	ZEB1	0.478132	≤0.0001
MBNL1-AS	FGF2	0.43659	4.37E-14
MBNL1-AS	FZD4	0.432872	7.55E-14
MBNL1-AS	TWIST2	0.341203	7.67E-09
MBNL1-AS	CXCL12	0.336813	1.22E-08
MCF2L-AS	ZEB1	-0.3872	3.68E-11
MCF2L-AS	FZD4	-0.32526	4.03E-08
MCF2L-AS	BIRC5	0.319421	7.22E-08
MCF2L-AS	TWIST2	-0.31265	1.40E-07
MCF2L-AS	CXCL12	-0.30276	3.58E-07
MCHR2-A	TGFB1	0.353967	1.90E-09
MCM8-AS	CDH2	0.307718	2.24E-07
MED14OS	TGFB1	0.413836	1.12E-12
MEF2C-AS	CXCL12	0.539741	≤0.0001
MEF2C-AS	BIRC5	-0.51886	≤0.0001
MEF2C-AS	ZEB1	0.5356	≤0.0001
MEF2C-AS	FGF2	0.437356	3.91E-14
MEF2C-AS	EGF	0.357718	1.24E-09

MEF2C-AS	FZD4	0.349138	3.24E-09
MEF2C-AS	TWIST2	0.330277	2.41E-08
MEF2C-AS	SNAI1	-0.31831	8.06E-08
MEF2C-AS	TWIST1	0.308403	2.10E-07
MEG3	CDH2	0.327742	3.13E-08
MEG3	ZEB1	0.318743	7.72E-08
MEG8	CDH2	0.352356	2.27E-09
MEG9	BIRC5	-0.38525	4.70E-11
MEG9	ZEB1	0.378255	1.11E-10
MEG9	CXCL12	0.317393	8.82E-08
MEIS1-AS:	CDH2	0.354885	1.71E-09
MEIS1-AS:	ZEB1	0.304544	3.03E-07
MEIS1-AS:	CDH2	0.411356	1.57E-12
MESTIT1	FZD4	0.723506	≤0.0001
MESTIT1	FGF2	0.577464	≤0.0001
MESTIT1	TWIST2	0.572322	≤0.0001
MESTIT1	ZEB1	0.633806	≤0.0001
MESTIT1	CXCL12	0.450776	5.11E-15
MESTIT1	BIRC5	-0.38609	4.23E-11
MESTIT1	TWIST1	0.345204	4.98E-09
MGAT3-A	FGF2	0.399696	7.43E-12
MGAT3-A	ZEB1	0.35867	1.12E-09
MGAT3-A	FZD4	0.352804	2.16E-09
MGC1588	ZEB1	0.33489	1.50E-08
MIAT	CXCR4	0.345685	4.73E-09
MIAT	TGFB1	0.336271	1.29E-08
MID1IP1-/	FZD4	0.493624	≤0.0001
MID1IP1-/	ZEB1	0.486754	≤0.0001
MID1IP1-/	TWIST2	0.450397	5.33E-15
MID1IP1-/	TWIST1	0.405705	3.36E-12
MID1IP1-/	FGF2	0.395516	1.28E-11
MIMT1	BIRC5	0.354119	1.86E-09
MIR100HG	CXCL12	0.584484	≤0.0001
MIR100HG	BIRC5	-0.54358	≤0.0001
MIR100HG	ZEB1	0.505049	≤0.0001
MIR100HG	FGF2	0.376394	1.39E-10
MIR124-2I	TGFB1	0.473784	≤0.0001
MIR137HG	CDH2	0.361994	7.62E-10
MIR155HG	CXCR4	0.378366	1.10E-10
MIR155HG	FZD4	-0.32908	2.73E-08
MIR155HG	BIRC5	0.323422	4.85E-08
MIR155HG	TGFB1	0.321186	6.06E-08
MIR155HG	CXCL12	-0.31937	7.26E-08
MIR155HG	FGF2	-0.3119	1.51E-07
MIR17HG	CDH2	0.304245	3.11E-07
MIR181A1	CDH2	0.328713	2.84E-08
MIR202HG	CXCL12	0.49944	≤0.0001
MIR202HG	BIRC5	-0.3354	1.42E-08
MIR205HG	EGF	0.403263	4.64E-12
MIR205HG	BIRC5	-0.38733	3.62E-11
MIR205HG	CXCL12	0.325709	3.85E-08
MIR210HG	CXCL12	-0.40664	2.96E-12
MIR210HG	ZEB1	-0.34701	4.09E-09
MIR210HG	FGF2	-0.3365	1.26E-08
MIR210HG	FZD4	-0.30885	2.02E-07
MIR22HG	CXCL12	0.721803	≤0.0001
MIR22HG	BIRC5	-0.5524	≤0.0001
MIR22HG	FZD4	0.738353	≤0.0001

MIR22HG	FGF2	0.707009	≤0.0001
MIR22HG	TWIST2	0.713225	≤0.0001
MIR22HG	ZEB1	0.705149	≤0.0001
MIR22HG	TWIST1	0.413861	1.11E-12
MIR22HG	CXCR4	-0.33991	8.80E-09
MIR31HG	HIF1A	0.37363	1.94E-10
MIR381HG	CDH2	0.35033	2.84E-09
MIR4697H	CXCL12	0.494697	≤0.0001
MIR4697H	BIRC5	-0.49112	≤0.0001
MIR4697H	EGF	0.354594	1.77E-09
MIR497HG	CXCL12	0.652665	≤0.0001
MIR497HG	BIRC5	-0.58621	≤0.0001
MIR497HG	FZD4	0.515844	≤0.0001
MIR497HG	FGF2	0.473517	≤0.0001
MIR497HG	TWIST2	0.572417	≤0.0001
MIR497HG	TWIST1	0.426798	1.82E-13
MIR497HG	HIF1A	-0.40904	2.15E-12
MIR497HG	ZEB1	0.408533	2.30E-12
MIR497HG	CXCR4	-0.34224	6.86E-09
MIR503HG	TGFB1	0.580129	≤0.0001
MIR762HG	CXCL12	-0.42685	1.80E-13
MIR762HG	FZD4	-0.41577	8.56E-13
MIR762HG	FGF2	-0.3973	1.01E-11
MIR762HG	ZEB1	-0.38915	2.88E-11
MIR762HG	BIRC5	0.362632	7.08E-10
MIR762HG	TWIST2	-0.35262	2.20E-09
MIR99AHG	CXCL12	0.553262	≤0.0001
MIR99AHG	BIRC5	-0.5424	≤0.0001
MIR99AHG	FGF2	0.482751	≤0.0001
MIR99AHG	ZEB1	0.549072	≤0.0001
MIR99AHG	FZD4	0.456063	2.22E-15
MIR99AHG	TWIST2	0.374398	1.77E-10
MIR99AHG	EGF	0.353112	2.09E-09
MIR99AHG	CXCR4	-0.30867	2.05E-07
MKLN1-AS	BIRC5	-0.40202	5.47E-12
MKLN1-AS	CXCL12	0.328798	2.81E-08
MKLN1-AS	EGF	0.316115	1.00E-07
MKLN1-AS	ZEB1	0.300113	4.57E-07
MKX-AS1	CDH2	0.351199	2.58E-09
MME-AS1	EGF	0.448641	7.11E-15
MME-AS1	BIRC5	-0.43271	7.73E-14
MME-AS1	CXCL12	0.414941	9.60E-13
MME-AS1	ZEB1	0.389746	2.67E-11
MME-AS1	FGF2	0.323148	4.98E-08
MMP25-A	FZD4	-0.36819	3.71E-10
MMP25-A	TWIST2	-0.33781	1.10E-08
MNX1-AS	BIRC5	0.373769	1.91E-10
MNX1-AS	CXCL12	-0.36233	7.33E-10
MNX1-AS	ZEB1	-0.33759	1.13E-08
MORF4L2-	EGF	0.397612	9.74E-12
MPRIP-AS	CDH2	0.345901	4.62E-09
MRGPRF-/	CXCL12	0.623244	≤0.0001
MRGPRF-/	BIRC5	-0.54559	≤0.0001
MRGPRF-/	FZD4	0.532134	≤0.0001
MRGPRF-/	FGF2	0.491427	≤0.0001
MRGPRF-/	TWIST2	0.510756	≤0.0001
MRGPRF-/	ZEB1	0.504599	≤0.0001
MRGPRF-/	TWIST1	0.336246	1.30E-08

MRGPRF-/	CXCR4	-0.30828	2.13E-07
MTHFS	BIRC5	0.324911	4.17E-08
MYB-AS1	EGF	0.3104	1.74E-07
MYCBP2-/	ZEB1	0.324216	4.48E-08
MYCNOS	CXCL12	-0.30343	3.36E-07
MYLK-AS1	CXCL12	-0.33334	1.76E-08
MYLK-AS1	BIRC5	0.331114	2.21E-08
MYO16-A:	CDH2	0.367967	3.81E-10
N4BP2L2-	ZEB1	0.32246	5.34E-08
NAALADL:	CDH2	0.31027	1.76E-07
NADK2-A:	ZEB1	0.403647	4.41E-12
NALCN-A:	HIF1A	0.307119	2.38E-07
NARF-IT1	CDH2	0.326227	3.65E-08
NARF-IT1	ZEB1	0.300195	4.54E-07
NCAM1-A	CDH2	0.300326	4.48E-07
NCBP2-A!	CXCL12	-0.5068	≤0.0001
NCBP2-A!	BIRC5	0.578551	≤0.0001
NCBP2-A!	ZEB1	-0.5712	≤0.0001
NCBP2-A!	FGF2	-0.45638	2.22E-15
NCBP2-A!	FZD4	-0.43155	9.15E-14
NCBP2-A!	TWIST2	-0.31024	1.77E-07
NDUFB2-/	ZEB1	-0.46025	1.11E-15
NDUFB2-/	CXCL12	-0.43551	5.11E-14
NDUFB2-/	FZD4	-0.42007	4.71E-13
NDUFB2-/	FGF2	-0.40221	5.34E-12
NDUFB2-/	TWIST2	-0.38086	8.09E-11
NDUFB2-/	BIRC5	0.32126	6.02E-08
NDUFB2-/	TWIST1	-0.3139	1.24E-07
NEBL-AS1	ZEB1	-0.30287	3.54E-07
NEXN-AS1	ZEB1	0.486436	≤0.0001
NFIA-AS1	CDH2	0.346324	4.41E-09
NFYC-AS1	TWIST2	-0.30331	3.40E-07
NIFK-AS1	CXCL12	0.463821	6.66E-16
NIFK-AS1	FGF2	0.378649	1.06E-10
NIFK-AS1	HIF1A	-0.37357	1.96E-10
NIFK-AS1	BIRC5	-0.34302	6.31E-09
NLGN1-A!	CDH2	0.354258	1.84E-09
NOP14-A!	BIRC5	-0.37111	2.63E-10
NPTN-IT1	ZEB1	0.404707	3.83E-12
NR2F1-AS	CXCL12	0.656887	≤0.0001
NR2F1-AS	BIRC5	-0.52808	≤0.0001
NR2F1-AS	FZD4	0.557315	≤0.0001
NR2F1-AS	FGF2	0.499327	≤0.0001
NR2F1-AS	ZEB1	0.659608	≤0.0001
NR2F1-AS	TWIST1	0.516724	≤0.0001
NR2F1-AS	TWIST2	0.458109	1.78E-15
NR2F2-AS	ZEB1	0.442007	1.95E-14
NR2F2-AS	BIRC5	-0.30069	4.33E-07
NRG1-IT1	CDH2	0.352156	2.32E-09
NRG1-IT3	CDH2	0.349933	2.97E-09
NRG3-AS1	CDH2	0.354118	1.86E-09
OGFRP1	CXCL12	-0.34147	7.45E-09
OGFRP1	FZD4	-0.31799	8.32E-08
OIP5-AS1	CXCL12	0.552487	≤0.0001
OIP5-AS1	BIRC5	-0.49396	≤0.0001
OIP5-AS1	FZD4	0.485846	≤0.0001
OIP5-AS1	FGF2	0.558273	≤0.0001
OIP5-AS1	ZEB1	0.593765	≤0.0001

OIP5-AS1	EGF	0.372804	2.15E-10
OIP5-AS1	SNAI1	-0.3459	4.62E-09
OIP5-AS1	TGFB1	-0.34529	4.94E-09
OR2A1-AS	EGF	0.397594	9.77E-12
OR2A1-AS	BIRC5	-0.39286	1.80E-11
OR2A1-AS	CXCL12	0.367755	3.90E-10
OXCT1-AS	CXCL12	0.485085	≤0.0001
OXCT1-AS	FZD4	0.683691	≤0.0001
OXCT1-AS	FGF2	0.670004	≤0.0001
OXCT1-AS	TWIST2	0.627756	≤0.0001
OXCT1-AS	ZEB1	0.524331	≤0.0001
OXCT1-AS	TWIST1	0.499776	≤0.0001
OXCT1-AS	BIRC5	-0.41569	8.66E-13
OXCT1-AS	SNAI1	-0.3274	3.24E-08
OXCT1-AS	CXCR4	-0.30204	3.82E-07
P4HA2-AS	BIRC5	-0.31982	6.94E-08
PABPC5-A	ZEB1	0.408788	2.22E-12
PAQR9-AS	CDH2	0.3971	1.04E-11
PAQR9-AS	ZEB1	0.307881	2.21E-07
PARD3-AS	EGF	0.346084	4.53E-09
PART1	ZEB1	-0.33542	1.42E-08
PART1	FZD4	-0.32611	3.70E-08
PART1	TWIST2	-0.31516	1.10E-07
PAXBP1-A	ZEB1	0.389725	2.68E-11
PAXBP1-A	BIRC5	-0.3035	3.34E-07
PAXIP1-AS	ZEB1	-0.3516	2.47E-09
PAXIP1-AS	CXCL12	0.671521	≤0.0001
PAXIP1-AS	BIRC5	-0.62387	≤0.0001
PAXIP1-AS	FGF2	0.543329	≤0.0001
PAXIP1-AS	EGF	0.509931	≤0.0001
PAXIP1-AS	ZEB1	0.536679	≤0.0001
PAXIP1-AS	FZD4	0.446267	1.02E-14
PAXIP1-AS	SNAI1	-0.33947	9.22E-09
PAXIP1-AS	TWIST2	0.302098	3.80E-07
PCAT19	CXCL12	0.569474	≤0.0001
PCAT19	FZD4	0.509722	≤0.0001
PCAT19	TWIST2	0.507894	≤0.0001
PCAT19	ZEB1	0.444098	1.42E-14
PCAT19	FGF2	0.404048	4.19E-12
PCAT19	BIRC5	-0.38536	4.63E-11
PCAT19	TWIST1	0.317127	9.06E-08
PCAT4	ZEB1	0.408651	2.26E-12
PCAT6	CXCL12	-0.46266	8.88E-16
PCAT6	ZEB1	-0.43598	4.80E-14
PCAT6	FZD4	-0.36531	5.19E-10
PCAT6	FGF2	-0.35876	1.10E-09
PCAT6	BIRC5	0.354554	1.78E-09
PCAT6	TWIST2	-0.32181	5.69E-08
PCAT7	CXCL12	-0.3351	1.46E-08
PCAT7	CDH2	0.301551	4.00E-07
PCBP1-AS	CDH2	0.34468	5.27E-09
PCBP1-AS	ZEB1	0.330006	2.48E-08
PCDH9-AS	CDH2	0.344265	5.52E-09
PCDH9-AS	CDH2	0.349471	3.12E-09
PCED1B-A	TGFB1	0.410676	1.72E-12
PCED1B-A	CXCR4	0.370831	2.71E-10
PDZRN3-A	ZEB1	0.346119	4.51E-09
PDZRN3-A	CDH2	0.332981	1.83E-08

PEX5L-AS:	CDH2	0.380808	8.14E-11
PEX5L-AS:	CDH2	0.397507	9.88E-12
PGM5-AS:	CXCL12	0.799087	≤0.0001
PGM5-AS:	BIRC5	-0.56475	≤0.0001
PGM5-AS:	FZD4	0.733072	≤0.0001
PGM5-AS:	FGF2	0.667451	≤0.0001
PGM5-AS:	TWIST2	0.71236	≤0.0001
PGM5-AS:	ZEB1	0.700356	≤0.0001
PGM5-AS:	TWIST1	0.483992	≤0.0001
PGM5-AS:	CXCR4	-0.34948	3.12E-09
PGM5-AS:	HIF1A	-0.31932	7.29E-08
PGM5P2	ZEB1	0.374475	1.76E-10
PGM5P3-/	CXCL12	0.59681	≤0.0001
PGM5P3-/	BIRC5	-0.4735	≤0.0001
PGM5P3-/	FZD4	0.730871	≤0.0001
PGM5P3-/	FGF2	0.704257	≤0.0001
PGM5P3-/	TWIST2	0.672992	≤0.0001
PGM5P3-/	ZEB1	0.622593	≤0.0001
PGM5P3-/	TWIST1	0.553318	≤0.0001
PGM5P3-/	CXCR4	-0.30372	3.27E-07
PGM5P4-/	CXCL12	0.544813	≤0.0001
PGM5P4-/	FZD4	0.644764	≤0.0001
PGM5P4-/	FGF2	0.653595	≤0.0001
PGM5P4-/	TWIST2	0.618274	≤0.0001
PGM5P4-/	ZEB1	0.55024	≤0.0001
PGM5P4-/	TWIST1	0.545075	≤0.0001
PGM5P4-/	BIRC5	-0.41659	7.65E-13
PHEX-AS1	EGF	0.433967	6.44E-14
PHKA2-AS	TWIST1	0.30136	4.07E-07
PIK3CD-A:	ZEB1	-0.33941	9.29E-09
PIK3CD-A:	FZD4	-0.31281	1.38E-07
PINK1-AS	EGF	0.361732	7.86E-10
PITPNA-A	HIF1A	-0.36178	7.81E-10
PLBD1-AS	ZEB1	0.33004	2.47E-08
PLBD1-AS	FGF2	0.300906	4.25E-07
PLCE1-AS:	CDH2	0.321115	6.11E-08
PLCE1-AS:	CDH2	0.323093	5.01E-08
PLCG1-AS	ZEB1	0.331541	2.12E-08
PLCG1-AS	CDH2	0.30149	4.02E-07
POU6F2-A	CDH2	0.310085	1.79E-07
PP7080	ZEB1	-0.45391	3.11E-15
PP7080	FZD4	-0.40985	1.92E-12
PP7080	CXCL12	-0.34585	4.64E-09
PP7080	BIRC5	0.336405	1.28E-08
PP7080	TWIST2	-0.32567	3.86E-08
PP7080	TWIST1	-0.31504	1.11E-07
PP7080	FGF2	-0.30163	3.97E-07
PPEF1-AS:	CDH2	0.305726	2.71E-07
PPP3CB-A	BIRC5	-0.50994	≤0.0001
PPP3CB-A	CXCL12	0.445936	1.07E-14
PPP3CB-A	EGF	0.422489	3.35E-13
PPP3CB-A	ZEB1	0.306737	2.46E-07
PRICKLE2-	ZEB1	0.460972	8.88E-16
PRICKLE2-	ZEB1	0.343625	5.91E-09
PRKAR2A-	BIRC5	-0.39189	2.03E-11
PRKCQ-AS	FZD4	-0.3072	2.36E-07
PROX1-AS	CXCL12	0.334301	1.59E-08
PRSS51	EGF	0.333113	1.80E-08

PSMA3-AS	CXCL12	0.474965	≤0.0001
PSMA3-AS	BIRC5	-0.4949	≤0.0001
PSMA3-AS	EGF	0.479206	≤0.0001
PSMB8-AS	TGFB1	0.341261	7.62E-09
PSMD6-AS	ZEB1	0.360098	9.48E-10
PSMG3-AS	CXCL12	0.510804	≤0.0001
PSMG3-AS	BIRC5	-0.45048	5.33E-15
PSMG3-AS	FZD4	0.426168	1.99E-13
PSMG3-AS	FGF2	0.387838	3.40E-11
PSMG3-AS	ZEB1	0.381052	7.90E-11
PSMG3-AS	EGF	0.3146	1.16E-07
PSMG3-AS	TWIST2	0.310892	1.66E-07
PSORS1C3	PDGFB	0.37398	1.86E-10
PTENP1-A	FZD4	0.647346	≤0.0001
PTENP1-A	FGF2	0.647484	≤0.0001
PTENP1-A	TWIST2	0.606268	≤0.0001
PTENP1-A	ZEB1	0.593862	≤0.0001
PTENP1-A	TWIST1	0.517021	≤0.0001
PTENP1-A	CXCL12	0.462531	8.88E-16
PTENP1-A	BIRC5	-0.42497	2.36E-13
PTOV1-AS	FZD4	-0.39659	1.11E-11
PTOV1-AS	TWIST2	-0.37054	2.81E-10
PTOV1-AS	CXCL12	-0.31244	1.43E-07
PTOV1-AS	FGF2	-0.31119	1.61E-07
PTPRD-AS	ZEB1	0.408486	2.31E-12
PTPRD-AS	CXCL12	0.392096	1.98E-11
PTPRG-AS	FZD4	-0.3082	2.15E-07
PWAR5	ZEB1	0.402045	5.45E-12
PWAR6	CXCL12	0.499639	≤0.0001
PWAR6	BIRC5	-0.51965	≤0.0001
PWAR6	EGF	0.503893	≤0.0001
PWAR6	ZEB1	0.454208	3.11E-15
PWAR6	FGF2	0.367577	3.98E-10
PYCARD-A	ZEB1	-0.30931	1.93E-07
RAB11B-A	TWIST1	0.333534	1.72E-08
RAB11B-A	TWIST2	0.331702	2.08E-08
RAB11B-A	HIF1A	-0.30304	3.48E-07
RAD51-AS	BIRC5	-0.5109	≤0.0001
RAD51-AS	EGF	0.565201	≤0.0001
RAD51-AS	CXCL12	0.426068	2.02E-13
RAD51-AS	SNAI1	-0.30258	3.64E-07
RAMP2-AS	CXCL12	0.578789	≤0.0001
RAMP2-AS	FZD4	0.71435	≤0.0001
RAMP2-AS	FGF2	0.696471	≤0.0001
RAMP2-AS	TWIST2	0.62432	≤0.0001
RAMP2-AS	ZEB1	0.665232	≤0.0001
RAMP2-AS	TWIST1	0.569942	≤0.0001
RAMP2-AS	BIRC5	-0.46539	4.44E-16
RAMP2-AS	CXCR4	-0.30109	4.18E-07
RAP2C-AS	ZEB1	0.380544	8.41E-11
RAP2C-AS	BIRC5	-0.33451	1.56E-08
RAPGEF4-	ZEB1	0.476089	≤0.0001
RARA-AS1	CXCL12	0.311027	1.64E-07
RARA-AS1	BIRC5	-0.30943	1.91E-07
RASGRF2-	CXCL12	0.605416	≤0.0001
RASGRF2-	FZD4	0.7458	≤0.0001
RASGRF2-	FGF2	0.628523	≤0.0001
RASGRF2-	TWIST2	0.601933	≤0.0001

RASGRF2-	ZEB1	0.689851	≤0.0001
RASGRF2-	BIRC5	-0.46947	2.22E-16
RASGRF2-	TWIST1	0.452755	3.77E-15
RASGRF2-	CXCR4	-0.30324	3.42E-07
RASSF8-A	ZEB1	0.429697	1.20E-13
RASSF8-A	CXCL12	0.323859	4.64E-08
RBAKDN	TGFB1	0.361561	8.01E-10
RBM26-AS	EGF	0.38744	3.57E-11
RBM26-AS	BIRC5	-0.37693	1.31E-10
RBM26-AS	FGF2	0.376039	1.45E-10
RBM26-AS	CXCL12	0.351149	2.60E-09
RBM26-AS	SNAI1	-0.34209	6.97E-09
RBM26-AS	TGFB1	-0.32847	2.91E-08
RBM26-AS	ZEB1	0.32515	4.07E-08
RBM5-AS1	ZEB1	0.402914	4.86E-12
RBM5-AS1	BIRC5	-0.31549	1.06E-07
RBMS3-AS	CDH2	0.347227	4.00E-09
RBMS3-AS	CDH2	0.348873	3.34E-09
RBMS3-AS	CXCL12	0.547601	≤0.0001
RBMS3-AS	BIRC5	-0.52648	≤0.0001
RBMS3-AS	FZD4	0.543168	≤0.0001
RBMS3-AS	FGF2	0.539724	≤0.0001
RBMS3-AS	TWIST2	0.519066	≤0.0001
RBMS3-AS	ZEB1	0.619232	≤0.0001
RBMS3-AS	TWIST1	0.472454	≤0.0001
RBMS3-AS	CXCR4	-0.31754	8.70E-08
RBPMS-AS	FZD4	0.709686	≤0.0001
RBPMS-AS	FGF2	0.606897	≤0.0001
RBPMS-AS	TWIST2	0.660077	≤0.0001
RBPMS-AS	ZEB1	0.529616	≤0.0001
RBPMS-AS	TWIST1	0.618324	≤0.0001
RBPMS-AS	CXCL12	0.445024	1.24E-14
RBPMS-AS	BIRC5	-0.41311	1.23E-12
RERG-IT1	EGF	0.481723	≤0.0001
RERG-IT1	BIRC5	-0.45392	3.11E-15
RERG-IT1	CXCL12	0.42599	2.04E-13
RGS5	CXCL12	0.547646	≤0.0001
RGS5	FZD4	0.583288	≤0.0001
RGS5	FGF2	0.508998	≤0.0001
RGS5	ZEB1	0.637932	≤0.0001
RGS5	BIRC5	-0.46047	1.11E-15
RGS5	TWIST2	0.417293	6.93E-13
RGS5	TWIST1	0.327505	3.21E-08
RHOA-IT1	ZEB1	0.30201	3.83E-07
RHPN1-AS	CXCL12	-0.50531	≤0.0001
RHPN1-AS	BIRC5	0.494397	≤0.0001
RHPN1-AS	ZEB1	-0.4714	2.22E-16
RHPN1-AS	FZD4	-0.43529	5.28E-14
RHPN1-AS	TWIST2	-0.42801	1.53E-13
RHPN1-AS	FGF2	-0.3398	8.91E-09
RHPN1-AS	TWIST1	-0.32175	5.73E-08
RMST	EGF	0.337391	1.15E-08
RNASEH1-	BIRC5	0.514877	≤0.0001
RNASEH1-	ZEB1	-0.43579	4.93E-14
RNASEH1-	CXCL12	-0.38222	6.84E-11
RNASEH1-	FGF2	-0.36262	7.09E-10
RNASEH1-	FZD4	-0.32725	3.29E-08
RNASEH1-	TWIST2	-0.302	3.84E-07

RNF139-A	CXCL12	-0.33186	2.05E-08
RNF139-A	FZD4	-0.32984	2.53E-08
RNF139-A	TWIST2	-0.31568	1.04E-07
RNF144A-	BIRC5	0.344077	5.63E-09
RNF144A-	CXCL12	-0.34125	7.63E-09
RNF144A-	CDH2	0.303245	3.42E-07
RNF217-A	ZEB1	0.338876	9.83E-09
RNU6ATA(	FZD4	0.812268	≤0.0001
RNU6ATA(	FGF2	0.654602	≤0.0001
RNU6ATA(	TWIST2	0.721657	≤0.0001
RNU6ATA(	ZEB1	0.569338	≤0.0001
RNU6ATA(	TWIST1	0.475731	≤0.0001
RNU6ATA(	CXCL12	0.438333	3.38E-14
RNU6ATA(	BIRC5	-0.34092	7.90E-09
ROR1-AS1	CDH2	0.313565	1.28E-07
ROR1-AS1	ZEB1	0.311535	1.56E-07
RORA-AS2	CDH2	0.319281	7.32E-08
RORB-AS1	TWIST1	0.372668	2.18E-10
RORB-AS1	ZEB1	0.301149	4.15E-07
RPARP-AS	ZEB1	-0.35555	1.59E-09
RPARP-AS	TWIST2	-0.3177	8.56E-08
RPARP-AS	FZD4	-0.31254	1.41E-07
RTCA-AS1	EGF	0.350201	2.88E-09
RTCA-AS1	BIRC5	-0.34604	4.55E-09
RTCA-AS1	SNAI1	-0.3336	1.71E-08
RUVBL1-A	ZEB1	0.328312	2.95E-08
SALRNA1	CDH2	0.366791	4.37E-10
SALRNA1	ZEB1	0.312993	1.35E-07
SBF2-AS1	TGFB1	-0.30594	2.66E-07
SCAMP1-/	EGF	0.416204	8.06E-13
SCAMP1-/	BIRC5	-0.32073	6.34E-08
SCARNA9	ZEB1	0.306516	2.52E-07
SCHLAP1	HIF1A	0.326398	3.59E-08
SDCBP2-A	ZEB1	0.449062	6.66E-15
SEC24B-A	EGF	0.331009	2.24E-08
SEC62-AS	ZEB1	0.370477	2.83E-10
SEMA3B-/	TWIST2	0.512705	≤0.0001
SEMA3B-/	FZD4	0.47122	2.22E-16
SEMA3B-/	ZEB1	0.364485	5.72E-10
SEMA3B-/	FGF2	0.351756	2.43E-09
SEMA3B-/	TWIST1	0.319577	7.11E-08
SEMA3F-A	BIRC5	-0.43364	6.75E-14
SEMA3F-A	CXCL12	0.423558	2.88E-13
SEMA3F-A	ZEB1	0.401737	5.68E-12
SEMA3F-A	EGF	0.30706	2.39E-07
SEMA6A-/	ZEB1	0.400496	6.69E-12
SENCR	TWIST2	0.475778	≤0.0001
SENCR	CXCL12	0.426487	1.90E-13
SENCR	ZEB1	0.376044	1.45E-10
SENCR	TWIST1	0.352602	2.21E-09
SENCR	FZD4	0.327765	3.12E-08
SEPT7-AS	BIRC5	-0.54924	≤0.0001
SEPT7-AS	ZEB1	0.479607	≤0.0001
SEPT7-AS	CXCL12	0.458351	1.55E-15
SEPT7-AS	FGF2	0.380634	8.32E-11
SEPT7-AS	EGF	0.352343	2.27E-09
SEPT7-AS	FZD4	0.313523	1.29E-07
SERTAD4-	PDGFB	0.325951	3.76E-08

SFTA1P	FZD4	0.400092	7.05E-12
SFTA1P	TGFB1	0.365614	5.01E-10
SFTA1P	TWIST2	0.326233	3.65E-08
SFTA1P	ZEB1	0.313926	1.24E-07
SFTA1P	TWIST1	0.306504	2.52E-07
SGMS1-AS	CXCL12	0.641947	≤0.0001
SGMS1-AS	BIRC5	-0.65704	≤0.0001
SGMS1-AS	EGF	0.518936	≤0.0001
SGMS1-AS	FGF2	0.446867	9.33E-15
SGMS1-AS	ZEB1	0.436087	4.71E-14
SGMS1-AS	SNAI1	-0.37158	2.48E-10
SGMS1-AS	FZD4	0.341103	7.75E-09
SGMS1-AS	CXCR4	-0.3017	3.95E-07
SH3BP5-A	ZEB1	0.434628	5.84E-14
SH3BP5-A	BIRC5	-0.4277	1.59E-13
SH3BP5-A	CXCL12	0.415978	8.32E-13
SH3BP5-A	EGF	0.312371	1.44E-07
SH3RF3-A	ZEB1	0.406999	2.82E-12
SH3RF3-A	TWIST2	0.360725	8.82E-10
SH3RF3-A	FZD4	0.327594	3.18E-08
SHANK3	CXCL12	0.603138	≤0.0001
SHANK3	FZD4	0.684477	≤0.0001
SHANK3	FGF2	0.594401	≤0.0001
SHANK3	TWIST2	0.612237	≤0.0001
SHANK3	ZEB1	0.654036	≤0.0001
SHANK3	BIRC5	-0.46125	8.88E-16
SHANK3	TWIST1	0.436879	4.17E-14
SLC14A2-A	FZD4	0.615608	≤0.0001
SLC14A2-A	FGF2	0.54842	≤0.0001
SLC14A2-A	TWIST2	0.58527	≤0.0001
SLC14A2-A	ZEB1	0.611329	≤0.0001
SLC14A2-A	CXCL12	0.472027	2.22E-16
SLC14A2-A	BIRC5	-0.34443	5.42E-09
SLC16A1-A	CDH2	0.346329	4.41E-09
SLC25A21	CXCL12	0.513068	≤0.0001
SLC25A21	BIRC5	-0.53048	≤0.0001
SLC25A21	EGF	0.481744	≤0.0001
SLC25A21	FGF2	0.408672	2.25E-12
SLC25A21	SNAI1	-0.36033	9.23E-10
SLC25A21	TGFB1	-0.34059	8.18E-09
SLC25A25	CDH2	0.301213	4.13E-07
SLC25A30	EGF	0.467285	4.44E-16
SLC25A30	BIRC5	-0.44692	9.33E-15
SLC25A30	CXCL12	0.37299	2.10E-10
SLC7A11-A	CDH2	0.357397	1.29E-09
SLC8A1-A	CDH2	0.344113	5.61E-09
SLC9A9-A	CDH2	0.305685	2.72E-07
SLC9A9-A	CDH2	0.332265	1.97E-08
SLIT2-IT1	CDH2	0.350691	2.73E-09
SMAD1-A	CDH2	0.322269	5.44E-08
SMAD9-IT	ZEB1	0.356123	1.49E-09
SMAD9-IT	CDH2	0.322098	5.54E-08
SMCR5	CDH2	0.317613	8.63E-08
SMG7-AS	FZD4	-0.31331	1.31E-07
SMG7-AS	CXCL12	-0.30934	1.92E-07
SMG7-AS	TWIST2	-0.30074	4.31E-07
SNAI3-AS	TGFB1	0.357237	1.31E-09
SNCA-AS1	CXCL12	0.367489	4.03E-10

SNHG1	BIRC5	0.510644	≤0.0001
SNHG1	CXCL12	-0.42293	3.15E-13
SNHG1	FZD4	-0.40557	3.42E-12
SNHG1	ZEB1	-0.39777	9.54E-12
SNHG1	CDH2	0.374523	1.75E-10
SNHG1	TWIST2	-0.35698	1.35E-09
SNHG1	FGF2	-0.35358	1.98E-09
SNHG10	ZEB1	-0.38125	7.71E-11
SNHG10	BIRC5	0.375453	1.56E-10
SNHG10	FZD4	-0.3537	1.95E-09
SNHG10	CXCL12	-0.3455	4.83E-09
SNHG11	ZEB1	-0.30834	2.12E-07
SNHG12	BIRC5	0.454348	2.89E-15
SNHG12	FZD4	-0.41159	1.52E-12
SNHG12	ZEB1	-0.41019	1.84E-12
SNHG12	CXCL12	-0.40927	2.08E-12
SNHG12	FGF2	-0.40133	5.99E-12
SNHG12	TWIST2	-0.35118	2.59E-09
SNHG14	BIRC5	-0.43271	7.73E-14
SNHG14	EGF	0.424006	2.70E-13
SNHG14	ZEB1	0.414683	9.94E-13
SNHG14	CXCL12	0.366458	4.54E-10
SNHG15	BIRC5	0.436642	4.35E-14
SNHG15	ZEB1	-0.40819	2.41E-12
SNHG15	FZD4	-0.35826	1.17E-09
SNHG15	FGF2	-0.34448	5.39E-09
SNHG15	CXCL12	-0.33727	1.17E-08
SNHG16	BIRC5	0.467651	4.44E-16
SNHG16	CDH2	0.324572	4.32E-08
SNHG16	CXCL12	-0.31849	7.92E-08
SNHG17	CXCL12	-0.43319	7.19E-14
SNHG17	ZEB1	-0.42388	2.75E-13
SNHG17	BIRC5	0.420044	4.73E-13
SNHG17	FGF2	-0.41494	9.60E-13
SNHG17	FZD4	-0.40779	2.54E-12
SNHG17	CDH2	0.316675	9.47E-08
SNHG17	TWIST2	-0.30024	4.52E-07
SNHG20	BIRC5	0.44304	1.67E-14
SNHG20	FZD4	-0.37668	1.35E-10
SNHG20	CDH2	0.344787	5.21E-09
SNHG20	ZEB1	-0.33809	1.07E-08
SNHG20	FGF2	-0.33033	2.40E-08
SNHG20	TWIST2	-0.31153	1.56E-07
SNHG21	BIRC5	0.392338	1.92E-11
SNHG21	ZEB1	-0.3438	5.80E-09
SNHG21	FZD4	-0.32346	4.83E-08
SNHG21	CXCL12	-0.31762	8.63E-08
SNHG3	BIRC5	0.434888	5.62E-14
SNHG3	CXCL12	-0.37696	1.30E-10
SNHG3	FGF2	-0.33833	1.04E-08
SNHG3	FZD4	-0.33118	2.20E-08
SNHG4	EGF	0.322704	5.21E-08
SNHG6	BIRC5	0.444039	1.42E-14
SNHG6	ZEB1	-0.38912	2.89E-11
SNHG6	CXCL12	-0.35035	2.83E-09
SNHG7	HIF1A	-0.31329	1.32E-07
SNHG8	HIF1A	-0.34269	6.53E-09
SNHG8	EGF	0.327904	3.08E-08

SNHG9	ZEB1	-0.34599	4.57E-09
SOCS2-AS	BIRC5	-0.36357	6.36E-10
SORCS3-A	CDH2	0.352935	2.13E-09
SOX9-AS1	TWIST2	-0.30174	3.93E-07
SOX9-AS1	FZD4	-0.30099	4.21E-07
SP2-AS1	ZEB1	0.318478	7.93E-08
SPATA13	EGF	0.447113	8.88E-15
SPATA41	CXCL12	0.333532	1.72E-08
SPATA41	TWIST2	0.30734	2.33E-07
SPIN4-AS	BIRC5	-0.32058	6.44E-08
SPIN4-AS	EGF	0.300272	4.50E-07
SRGAP2-A	CDH2	0.335861	1.35E-08
SRGAP3-A	CDH2	0.310372	1.74E-07
SRP14-AS	CXCL12	0.565717	≤0.0001
SRP14-AS	BIRC5	-0.48563	≤0.0001
SRP14-AS	FZD4	0.538564	≤0.0001
SRP14-AS	FGF2	0.541042	≤0.0001
SRP14-AS	ZEB1	0.514814	≤0.0001
SRP14-AS	TWIST2	0.435987	4.77E-14
SRP14-AS	HIF1A	-0.36713	4.20E-10
SRP14-AS	CXCR4	-0.33128	2.18E-08
SSBP3-AS	ZEB1	0.357031	1.34E-09
SSSCA1-A	TWIST2	-0.31587	1.02E-07
SSSCA1-A	FZD4	-0.31017	1.78E-07
ST3GAL5-	ZEB1	0.344369	5.45E-09
ST7-AS2	CDH2	0.355418	1.61E-09
ST7-OT4	CDH2	0.35623	1.47E-09
ST8SIA6-A	CXCL12	-0.37845	1.09E-10
ST8SIA6-A	FGF2	-0.36436	5.80E-10
ST8SIA6-A	HIF1A	0.335606	1.39E-08
ST8SIA6-A	TWIST2	-0.3321	2.00E-08
STAM-AS	BIRC5	0.37612	1.44E-10
STAM-AS	CXCL12	-0.35341	2.02E-09
STAM-AS	TWIST2	-0.32564	3.88E-08
STAM-AS	FZD4	-0.3237	4.71E-08
STARD4-A	CDH2	0.373487	1.98E-10
STARD7-A	CDH2	0.345972	4.58E-09
STAU2-AS	BIRC5	0.309973	1.81E-07
STX17-AS	EGF	0.421422	3.89E-13
STXBP5-A	CXCL12	0.538374	≤0.0001
STXBP5-A	BIRC5	-0.51639	≤0.0001
STXBP5-A	ZEB1	0.521544	≤0.0001
STXBP5-A	FGF2	0.404378	4.01E-12
STXBP5-A	FZD4	0.356695	1.40E-09
SUCLG2-A	ZEB1	0.47094	2.22E-16
SUCLG2-A	BIRC5	-0.42788	1.55E-13
SUCLG2-A	EGF	0.420158	4.65E-13
SUCLG2-A	FGF2	0.397209	1.03E-11
SUCLG2-A	CXCL12	0.373825	1.90E-10
SUCLG2-A	TGFB1	-0.34032	8.43E-09
SUCLG2-A	FZD4	0.324687	4.27E-08
SYNE1-AS	FZD4	0.484909	≤0.0001
SYNE1-AS	FGF2	0.531021	≤0.0001
SYNE1-AS	ZEB1	0.494165	≤0.0001
SYNE1-AS	CXCL12	0.459345	1.33E-15
SYNE1-AS	TWIST2	0.419139	5.36E-13
SYNE1-AS	BIRC5	-0.38084	8.11E-11
TAPT1-AS	BIRC5	-0.46325	6.66E-16

TAPT1-AS	ZEB1	0.464162	6.66E-16
TAPT1-AS	CXCL12	0.362229	7.42E-10
TAPT1-AS	EGF	0.342897	6.39E-09
TAT-AS1	EGF	0.336979	1.20E-08
TAT-AS1	BIRC5	-0.30166	3.96E-07
TBL1XR1-/	CDH2	0.321703	5.76E-08
TBX2-AS1	TWIST1	0.468384	4.44E-16
TBX2-AS1	TGFB1	0.463467	6.66E-16
TBX2-AS1	ZEB1	0.337134	1.18E-08
TBX5-AS1	CXCL12	0.710026	≤0.0001
TBX5-AS1	BIRC5	-0.51887	≤0.0001
TBX5-AS1	FZD4	0.473701	≤0.0001
TBX5-AS1	FGF2	0.485345	≤0.0001
TBX5-AS1	TWIST2	0.487398	≤0.0001
TBX5-AS1	ZEB1	0.61663	≤0.0001
TESC-AS1	EGF	0.454026	3.11E-15
TESC-AS1	BIRC5	-0.307	2.40E-07
TEX26-AS:	ZEB1	0.402021	5.47E-12
TFAP2A-A	FGF2	-0.32701	3.37E-08
TFAP2A-A	FZD4	-0.32352	4.80E-08
TFAP2A-A	CXCL12	-0.32113	6.10E-08
TFAP2A-A	TWIST2	-0.316	1.01E-07
TGFB2-AS	CDH2	0.394946	1.38E-11
THRB-AS1	CXCL12	0.548936	≤0.0001
THRB-AS1	BIRC5	-0.56249	≤0.0001
THRB-AS1	FZD4	0.486564	≤0.0001
THRB-AS1	FGF2	0.467722	4.44E-16
THRB-AS1	ZEB1	0.454119	3.11E-15
THRB-AS1	TWIST2	0.423094	3.08E-13
THRB-AS1	EGF	0.418687	5.71E-13
THRB-AS1	CXCR4	-0.35887	1.09E-09
THRB-AS1	TWIST1	0.317499	8.73E-08
THUMPD3	TWIST2	-0.32053	6.47E-08
THUMPD3	FZD4	-0.31475	1.14E-07
TINCR	CXCL12	-0.3319	2.04E-08
TINCR	FGF2	-0.3152	1.09E-07
TINCR	ZEB1	-0.30145	4.04E-07
TIPARP-A!	ZEB1	-0.44071	2.35E-14
TIPARP-A!	FZD4	-0.34841	3.51E-09
TMEM108	CDH2	0.311849	1.51E-07
TMEM147	FZD4	-0.32005	6.79E-08
TMEM161I	EGF	0.328848	2.80E-08
TMEM191/	FZD4	-0.47257	≤0.0001
TMEM191/	ZEB1	-0.55833	≤0.0001
TMEM191/	CXCL12	-0.45661	2.00E-15
TMEM191/	FGF2	-0.44524	1.20E-14
TMEM191/	TWIST2	-0.37949	9.56E-11
TMEM191/	BIRC5	0.334881	1.50E-08
TMEM191(	ZEB1	-0.40579	3.32E-12
TMEM191(	FZD4	-0.36079	8.76E-10
TMEM191(	FGF2	-0.31994	6.86E-08
TMEM191(	TWIST2	-0.30967	1.86E-07
TMEM191(	CXCL12	-0.30654	2.51E-07
TMEM220	CXCL12	0.676192	≤0.0001
TMEM220	BIRC5	-0.6185	≤0.0001
TMEM220	FZD4	0.499503	≤0.0001
TMEM220	FGF2	0.561282	≤0.0001
TMEM220	ZEB1	0.502031	≤0.0001

TMEM220	EGF	0.458743	1.33E-15
TMEM220	TWIST2	0.434087	6.31E-14
TMEM220	SNAI1	-0.33002	2.48E-08
TMEM220	CXCR4	-0.31989	6.90E-08
TMEM246	FZD4	0.581696	≤0.0001
TMEM246	FGF2	0.51934	≤0.0001
TMEM246	TWIST2	0.472533	≤0.0001
TMEM246	ZEB1	0.587338	≤0.0001
TMEM246	CXCL12	0.380557	8.39E-11
TMEM246	TWIST1	0.307738	2.24E-07
TMEM246	BIRC5	-0.30351	3.33E-07
TMEM26-/	EGF	0.418868	5.57E-13
TMEM26-/	BIRC5	-0.38507	4.80E-11
TMEM75	CDH2	0.344532	5.36E-09
TMEM9B-/	EGF	0.59816	≤0.0001
TMEM9B-/	BIRC5	-0.46873	4.44E-16
TMEM9B-/	CXCL12	0.413725	1.13E-12
TMEM9B-/	SNAI1	-0.31631	9.82E-08
TMPO-AS	CXCL12	-0.52651	≤0.0001
TMPO-AS	BIRC5	0.627753	≤0.0001
TMPO-AS	ZEB1	-0.44447	1.33E-14
TMPO-AS	FGF2	-0.44012	2.58E-14
TMPO-AS	FZD4	-0.41379	1.12E-12
TMPO-AS	TWIST2	-0.4068	2.90E-12
TMPO-AS	CDH2	0.402018	5.47E-12
TP73-AS1	CXCL12	0.438763	3.15E-14
TP73-AS1	EGF	0.435321	5.28E-14
TP73-AS1	BIRC5	-0.42062	4.36E-13
TP73-AS1	SNAI1	-0.32721	3.30E-08
TPRG1-AS	CXCL12	0.537419	≤0.0001
TPRG1-AS	BIRC5	-0.4884	≤0.0001
TPRG1-AS	FZD4	0.753331	≤0.0001
TPRG1-AS	FGF2	0.617393	≤0.0001
TPRG1-AS	TWIST2	0.569154	≤0.0001
TPRG1-AS	ZEB1	0.58505	≤0.0001
TPRG1-AS	TWIST1	0.356049	1.50E-09
TPRG1-AS	CXCR4	-0.34872	3.39E-09
TPT1-AS1	EGF	0.485889	≤0.0001
TPT1-AS1	BIRC5	-0.39061	2.39E-11
TPT1-AS1	CXCL12	0.311464	1.57E-07
TPTEP1	EGF	0.446873	9.33E-15
TPTEP1	BIRC5	-0.31303	1.35E-07
TPTEP1	SNAI1	-0.31209	1.48E-07
TRAF3IP2-	CDH2	0.316354	9.77E-08
TRAF3IP2-	ZEB1	0.316283	9.84E-08
TRAM2-AS	FGF2	0.453047	3.55E-15
TRAM2-AS	CXCL12	0.441981	1.95E-14
TRAM2-AS	ZEB1	0.415196	9.27E-13
TRAM2-AS	FZD4	0.413822	1.12E-12
TRAM2-AS	BIRC5	-0.38056	8.39E-11
TRAM2-AS	SNAI1	-0.35647	1.43E-09
TRG-AS1	CXCR4	0.35257	2.22E-09
TRG-AS1	TGFB1	0.310178	1.78E-07
TRHDE-AS	CXCL12	0.494148	≤0.0001
TRHDE-AS	FZD4	0.888263	≤0.0001
TRHDE-AS	FGF2	0.771976	≤0.0001
TRHDE-AS	TWIST2	0.788942	≤0.0001
TRHDE-AS	ZEB1	0.698605	≤0.0001

TRHDE-AS	TWIST1	0.615565	≤0.0001
TRHDE-AS	BIRC5	-0.38517	4.75E-11
TRIM52-A	CXCL12	0.489659	≤0.0001
TRIM52-A	FZD4	0.523466	≤0.0001
TRIM52-A	TWIST2	0.571095	≤0.0001
TRIM52-A	TWIST1	0.495226	≤0.0001
TRIM52-A	FGF2	0.448796	6.88E-15
TRIM52-A	ZEB1	0.408539	2.30E-12
TRIM52-A	HIF1A	-0.38811	3.28E-11
TRIM52-A	BIRC5	-0.37174	2.44E-10
TRIM52-A	CXCR4	-0.31124	1.60E-07
TRPM2-AS	FGF2	-0.41026	1.82E-12
TRPM2-AS	ZEB1	-0.39986	7.27E-12
TRPM2-AS	CXCL12	-0.37426	1.80E-10
TRPM2-AS	FZD4	-0.35112	2.60E-09
TRPM2-AS	TWIST2	-0.31554	1.06E-07
TSPEAR-A	CDH2	0.318386	8.00E-08
TSPEAR-A	CDH2	0.323708	4.71E-08
TSTD3	CXCL12	0.566374	≤0.0001
TSTD3	FZD4	0.640603	≤0.0001
TSTD3	FGF2	0.580679	≤0.0001
TSTD3	TWIST2	0.566537	≤0.0001
TSTD3	ZEB1	0.508058	≤0.0001
TSTD3	BIRC5	-0.41433	1.04E-12
TSTD3	TWIST1	0.380819	8.13E-11
TSTD3	CXCR4	-0.31775	8.52E-08
TSTD3	HIF1A	-0.30119	4.14E-07
TTC28-AS	EGF	0.368836	3.44E-10
TTC39A-A	BIRC5	-0.34036	8.39E-09
TTC3-AS1	CDH2	0.30366	3.29E-07
TTLL10-AS	FZD4	0.553222	≤0.0001
TTLL10-AS	TWIST2	0.53499	≤0.0001
TTLL10-AS	ZEB1	0.576822	≤0.0001
TTLL10-AS	CXCL12	0.448272	7.55E-15
TTLL10-AS	FGF2	0.424369	2.57E-13
TTLL10-AS	BIRC5	-0.38798	3.34E-11
TTLL10-AS	TWIST1	0.354173	1.85E-09
TTLL11-IT	HIF1A	0.390678	2.37E-11
TTLL7-IT1	CDH2	0.315298	1.08E-07
TTN-AS1	EGF	0.444845	1.27E-14
TTN-AS1	BIRC5	-0.36233	7.34E-10
TTN-AS1	CXCL12	0.307236	2.35E-07
TUB-AS1	CDH2	0.330861	2.27E-08
TUG1	TWIST2	-0.36712	4.20E-10
TUSC7	CDH2	0.354523	1.78E-09
TUSC8	EGF	0.536588	≤0.0001
TUSC8	BIRC5	-0.40449	3.94E-12
TYMSOS	BIRC5	0.660395	≤0.0001
TYMSOS	CXCL12	-0.4423	1.87E-14
TYMSOS	ZEB1	-0.42059	4.38E-13
TYMSOS	FGF2	-0.36426	5.87E-10
TYMSOS	FZD4	-0.34671	4.23E-09
TYMSOS	TWIST2	-0.31463	1.16E-07
U91324.1	FZD4	0.600977	≤0.0001
U91324.1	TWIST2	0.498595	≤0.0001
U91324.1	ZEB1	0.539169	≤0.0001
U91324.1	FGF2	0.428236	1.48E-13
U91324.1	CXCL12	0.385202	4.73E-11

UBA6-AS1	TWIST2	-0.32968	2.57E-08
UBE2E2-A	FGF2	0.49388	≤0.0001
UBE2E2-A	FZD4	0.398066	9.19E-12
UBE2E2-A	TWIST2	0.310623	1.70E-07
UBL7-AS1	EGF	0.340016	8.70E-09
UBXN7-AS	CDH2	0.30303	3.49E-07
UCKL1-AS	CDH2	0.327534	3.20E-08
UGDH-AS	BIRC5	-0.49182	≤0.0001
UGDH-AS	EGF	0.575865	≤0.0001
UGDH-AS	CXCL12	0.42266	3.27E-13
UMAD1	BIRC5	-0.47768	≤0.0001
UMAD1	CXCL12	0.411447	1.55E-12
UMAD1	EGF	0.376549	1.37E-10
UNC5B-AS	BIRC5	0.363034	6.76E-10
UNQ6494	CXCL12	-0.3149	1.13E-07
UNQ6494	FGF2	-0.30796	2.19E-07
URB1-AS1	ZEB1	-0.42247	3.36E-13
URB1-AS1	CXCL12	-0.34452	5.36E-09
USP27X-A	EGF	0.365199	5.26E-10
USP2-AS1	CXCL12	-0.4781	≤0.0001
USP2-AS1	BIRC5	0.524024	≤0.0001
USP2-AS1	ZEB1	-0.40608	3.19E-12
USP2-AS1	FZD4	-0.38866	3.07E-11
USP2-AS1	FGF2	-0.35369	1.96E-09
USP2-AS1	TWIST2	-0.3289	2.78E-08
USP30-AS	TGFB1	0.33533	1.43E-08
USP30-AS	CXCL12	-0.31201	1.49E-07
USP46-AS	FGF2	0.482677	≤0.0001
USP46-AS	CXCL12	0.433881	6.51E-14
USP46-AS	ZEB1	0.432269	8.24E-14
USP46-AS	BIRC5	-0.4221	3.54E-13
USP46-AS	FZD4	0.405381	3.50E-12
VENTXP1	CDH2	0.345835	4.65E-09
VIM-AS1	ZEB1	-0.34552	4.81E-09
VIM-AS1	FZD4	-0.33637	1.28E-08
VIM-AS1	CXCL12	-0.325	4.13E-08
VIM-AS1	TWIST2	-0.32041	6.55E-08
VIPR1-AS1	BIRC5	-0.49716	≤0.0001
VIPR1-AS1	EGF	0.570374	≤0.0001
VIPR1-AS1	CXCL12	0.365075	5.34E-10
VIPR1-AS1	SNAI1	-0.34183	7.17E-09
VLDLR-AS	FZD4	0.358401	1.15E-09
VPS9D1-A	BIRC5	0.506902	≤0.0001
VPS9D1-A	CXCL12	-0.40812	2.43E-12
VPS9D1-A	ZEB1	-0.39643	1.14E-11
VPS9D1-A	FZD4	-0.37442	1.77E-10
VPS9D1-A	FGF2	-0.31395	1.23E-07
WARS2-IT	ZEB1	0.586354	≤0.0001
WARS2-IT	CXCL12	0.440836	2.31E-14
WARS2-IT	BIRC5	-0.3952	1.33E-11
WARS2-IT	TWIST1	0.334069	1.63E-08
WARS2-IT	TWIST2	0.320441	6.53E-08
WARS2-IT	FZD4	0.320129	6.73E-08
WASF3-AS	CDH2	0.34038	8.37E-09
WASIR1	CDH2	0.47443	≤0.0001
WASIR2	ZEB1	-0.30848	2.09E-07
WASIR2	TWIST2	-0.30356	3.32E-07
WDFY3-AS	CXCL12	0.801176	≤0.0001

WDFY3-AS	BIRC5	-0.66978	≤0.0001
WDFY3-AS	FZD4	0.640315	≤0.0001
WDFY3-AS	FGF2	0.729959	≤0.0001
WDFY3-AS	TWIST2	0.523509	≤0.0001
WDFY3-AS	ZEB1	0.691519	≤0.0001
WDFY3-AS	TWIST1	0.407473	2.65E-12
WDFY3-AS	CXCR4	-0.3969	1.07E-11
WDFY3-AS	EGF	0.338586	1.01E-08
WDFY3-AS	SNAI1	-0.30819	2.15E-07
WDR86-A	CXCL12	0.40703	2.81E-12
WDR86-A	BIRC5	-0.38575	4.41E-11
WDR86-A	FGF2	0.30804	2.18E-07
WEE2-AS1	EGF	0.379369	9.71E-11
WEE2-AS1	BIRC5	-0.3148	1.14E-07
WFDC21P	CXCL12	-0.30294	3.52E-07
WWC2-AS	CXCL12	0.564354	≤0.0001
WWC2-AS	BIRC5	-0.58095	≤0.0001
WWC2-AS	FZD4	0.567176	≤0.0001
WWC2-AS	FGF2	0.6381	≤0.0001
WWC2-AS	TWIST2	0.539309	≤0.0001
WWC2-AS	ZEB1	0.470288	2.22E-16
WWC2-AS	CXCR4	-0.35616	1.48E-09
WWC2-AS	TWIST1	0.319994	6.83E-08
WWTR1-A	FZD4	-0.36343	6.46E-10
WWTR1-A	ZEB1	-0.32266	5.24E-08
WWTR1-IT	CDH2	0.306158	2.60E-07
XIST	ZEB1	0.362882	6.88E-10
XXYLT1-A	CDH2	0.308109	2.16E-07
YEATS2-A	CDH2	0.300379	4.46E-07
YTHDF3-A	TGFB1	0.359965	9.62E-10
Z69720.2	FGF2	-0.33896	9.74E-09
ZBED3-AS	FZD4	0.523747	≤0.0001
ZBED3-AS	TWIST2	0.511153	≤0.0001
ZBED3-AS	ZEB1	0.528408	≤0.0001
ZBED3-AS	TWIST1	0.517485	≤0.0001
ZBED3-AS	FGF2	0.410066	1.87E-12
ZBED3-AS	BIRC5	-0.36453	5.69E-10
ZBED3-AS	CXCL12	0.311436	1.57E-07
ZBTB11-A	BIRC5	0.326392	3.59E-08
ZBTB20-A	CDH2	0.302408	3.70E-07
ZBTB20-A	ZEB1	0.386233	4.16E-11
ZBTB40-IT	ZEB1	0.355944	1.52E-09
ZEB2-AS1	FZD4	0.565569	≤0.0001
ZEB2-AS1	TWIST2	0.500616	≤0.0001
ZEB2-AS1	ZEB1	0.654586	≤0.0001
ZEB2-AS1	CXCL12	0.437198	4.00E-14
ZEB2-AS1	TWIST1	0.436044	4.75E-14
ZEB2-AS1	FGF2	0.412513	1.34E-12
ZEB2-AS1	BIRC5	-0.37607	1.45E-10
ZFAS1	ZEB1	-0.3373	1.16E-08
ZFHX4-AS	ZEB1	0.36194	7.67E-10
ZFHX4-AS	TWIST1	0.354327	1.82E-09
ZFHX4-AS	TWIST2	0.315669	1.04E-07
ZFPM2-AS	CDH2	0.327066	3.35E-08
ZMIZ1-AS	EGF	0.349646	3.06E-09
ZNF30-AS	EGF	0.401019	6.24E-12
ZNF30-AS	BIRC5	-0.39178	2.06E-11
ZNF30-AS	SNAI1	-0.33271	1.88E-08

ZNF341-A	CXCL12	0.306104	2.61E-07
ZNF436-A	CXCL12	0.513862	≤0.0001
ZNF436-A	FZD4	0.629488	≤0.0001
ZNF436-A	FGF2	0.526042	≤0.0001
ZNF436-A	TWIST2	0.510398	≤0.0001
ZNF436-A	ZEB1	0.645203	≤0.0001
ZNF436-A	BIRC5	-0.45365	3.11E-15
ZNF436-A	TWIST1	0.352869	2.14E-09
ZNF582-A	CXCL12	0.554551	≤0.0001
ZNF582-A	BIRC5	-0.5556	≤0.0001
ZNF582-A	EGF	0.379327	9.76E-11
ZNF582-A	FGF2	0.36824	3.69E-10
ZNF582-A	ZEB1	0.306335	2.56E-07
ZNF883	ZEB1	-0.36453	5.69E-10
ZNF883	CXCL12	-0.35472	1.74E-09
ZNF883	FZD4	-0.34196	7.07E-09
ZNF883	BIRC5	0.334864	1.50E-08
ZNF883	TWIST2	-0.33235	1.95E-08
ZRANB2-A	ZEB1	0.454729	2.66E-15
ZRANB2-A	BIRC5	-0.35896	1.08E-09
ZRANB2-A	CXCL12	0.323204	4.95E-08
ZRANB2-A	ZEB1	0.387681	3.47E-11
ZSCAN16-	TWIST2	0.533997	≤0.0001
ZSCAN16-	TWIST1	0.576353	≤0.0001
ZSCAN16-	FZD4	0.449559	6.22E-15
ZSCAN16-	ZEB1	0.328978	2.76E-08
ZSCAN16-	CXCL12	0.315045	1.11E-07
ZSCAN16-	FGF2	0.306176	2.60E-07
