# Supplementary material for: Development of a Prognostic Model Based on the Identification of EMT-Related lncRNAs in Triple-Negative Breast Cancer
Source: J Oncol. 2021 Nov 27;2021:9219961. doi: 10.1155/2021/9219961 (PMC8643262; doi:10.1155/2021/9219961)
Supplement: Supplementary Materials — Supplementary Figure 1: prognostic lncRNAs were screened out from TNBC data. (A) Cox univariate regression analysis. (B) Lasso regression analysis. Supplementary Figure 2: CeRNA network. Supplementary Table 1: primers used in qRT-PCR. Supplementary Table 2: a total of 1033 lncRNAs highly associated with EMT. Supplementary Table 3: a total of 285 prognostic lncRNAs screened by Cox regression analysis. [file 9219961.f1.zip › 9219961.f1/Table S3 (1).pdf]

gene	HR	z	pvalue	lower	upper
YTHDF3-A	1.764467	4.808526	1.52E-06	1.399889	2.223995
UBE2E2-A	1.578622	3.336083	0.00085	1.207227	2.064274
SOCS2-AS	1.367147	3.157191	0.001593	1.125906	1.660078
TINCR	1.457961	2.781001	0.005419	1.117745	1.901731
A2M-AS1	0.383716	-2.74197	0.006107	0.19349	0.760958
CYB561D2	1.441663	2.684848	0.007256	1.103804	1.882938
TUG1	0.472494	-2.66239	0.007759	0.272081	0.820531
NIFK-AS1	0.452269	-2.60589	0.009164	0.249007	0.821453
LINC0066	0.48135	-2.58671	0.00969	0.276604	0.837649
NDUFB2-A	1.448511	2.463441	0.013761	1.078671	1.945157
CASC15	0.181184	-2.31269	0.02074	0.042598	0.770639
PINK1-AS	0.521389	-2.27136	0.023125	0.297235	0.914584
ZSCAN16-	1.44251	2.19584	0.028103	1.040142	2.000531
EPB41L4A	1.353094	2.156988	0.031007	1.028006	1.780984
TRIM52-A	1.53284	2.147651	0.031741	1.038032	2.263512
LINC0083	1.462265	2.120345	0.033977	1.029159	2.077638
ASB16-AS	0.580598	-2.11988	0.034016	0.351207	0.959815
RGS5	1.304484	2.09526	0.036148	1.017312	1.672721
LINC0102	1.364682	2.079744	0.037549	1.018068	1.829304
SLC16A1-	0.383245	-2.06542	0.038883	0.154248	0.952212
MBNL1-A	0.522008	-2.0399	0.04136	0.279523	0.974847
LINC0131	0.578999	-2.01446	0.043961	0.340233	0.985326
RNF144A-	0.44389	-1.9404	0.052331	0.195431	1.008221
MCF2L-AS	0.475341	-1.92128	0.054696	0.222591	1.015086
TRAF3IP2-	0.360138	-1.87382	0.060955	0.123751	1.048069
LINC0052	0.59665	-1.87327	0.061031	0.347584	1.024187
PCAT19	1.574921	1.853632	0.063792	0.974281	2.545852
GHET1	0.54833	-1.84693	0.064758	0.28981	1.03746
ALMS1-IT	0.529822	-1.84324	0.065294	0.269644	1.041046
KIAA0087	0.430699	-1.82719	0.067671	0.174488	1.063121
LINC0089	0.604138	-1.76531	0.077512	0.345254	1.057142
LINC0067	1.303467	1.752323	0.079718	0.969084	1.753231
CLSTN2-A	0.187008	-1.7453	0.080932	0.028455	1.229011
DANCR	1.392538	1.742121	0.081487	0.95944	2.02114
DDX11-AS	0.585043	-1.71875	0.085661	0.317469	1.078137
SH3BP5-A	0.525656	-1.7115	0.086988	0.251685	1.097858
USP30-AS	0.548476	-1.70856	0.087532	0.275381	1.092399
PPP3CB-A	0.550001	-1.70506	0.088184	0.276638	1.093492
HCG11	0.659631	-1.68973	0.09108	0.407102	1.068806
CCNT2-AS	0.592556	-1.67649	0.093642	0.321388	1.092519
SNAI3-AS	1.365453	1.646023	0.099759	0.942321	1.978582
PSMD6-A	0.26444	-1.61937	0.105368	0.052864	1.322814
PSORS1C3	0.181407	-1.60847	0.107733	0.022662	1.452129
HEIH	1.365192	1.607029	0.108048	0.933918	1.995622
HCG18	0.349665	-1.60476	0.108546	0.096894	1.261855
BLACAT1	0.534349	-1.59966	0.109675	0.247939	1.151608
AGAP2-AS	1.355975	1.572601	0.115811	0.927735	1.98189
MYLK-AS1	1.2906	1.553729	0.120249	0.935476	1.780537
TRAM2-AS	0.648969	-1.55053	0.121014	0.37572	1.120945
SLC25A21	0.595131	-1.54324	0.122773	0.307868	1.150434
DKFZp779	1.295331	1.534913	0.124805	0.930849	1.802529
TUSC8	1.298765	1.505191	0.132275	0.924056	1.82542
DGCR9	0.608882	-1.50337	0.132745	0.318878	1.162629
SFTA1P	1.873386	1.494309	0.135095	0.822326	4.267864
FGD5-AS1	0.670983	-1.48516	0.137502	0.396298	1.136058
LINC0142	1.180095	1.483155	0.138033	0.948156	1.468771
LINC0047	0.565143	-1.48216	0.138297	0.265718	1.201976

LINC0114(	0.567445	-1.46929	0.141754	0.266483	1.208308
PCAT6	1.297629	1.466423	0.142533	0.916048	1.838158
ZNF883	1.219191	1.45753	0.14497	0.933963	1.591528
CELF2-AS:	0.353082	-1.40864	0.158941	0.082946	1.502987
PSMG3-AS	1.224679	1.39909	0.161786	0.921962	1.626791
RBAKDN	1.196885	1.380401	0.167463	0.92732	1.544812
MIAT	0.666307	-1.36722	0.171557	0.37231	1.192461
ACVR2B-A	0.623052	-1.36603	0.171931	0.316016	1.228399
CNTFR-AS	0.395838	-1.34914	0.177293	0.102994	1.521335
TBX5-AS1	0.646969	-1.33553	0.181702	0.341464	1.225806
LINC00997	1.310588	1.322273	0.186077	0.877707	1.956964
TTC28-AS	0.686065	-1.3149	0.188544	0.391251	1.203029
PCBP1-AS	0.366946	-1.30833	0.190762	0.081724	1.647622
STARD7-A	0.689604	-1.30212	0.192876	0.394147	1.206538
UNC5B-AS	0.59871	-1.28739	0.19796	0.274185	1.307345
ARHGEF26	0.705121	-1.28371	0.199243	0.413612	1.202082
CDKN2B-A	0.662128	-1.27485	0.202362	0.351283	1.248035
C10orf95	1.381722	1.259144	0.207978	0.835303	2.285586
LINC01278	0.724903	-1.25395	0.209861	0.438422	1.198581
RAB11B-A	1.225957	1.249458	0.211498	0.890612	1.687569
SCAMP1-A	1.249429	1.246044	0.212748	0.880216	1.773511
MIR17HG	0.654169	-1.2317	0.218059	0.33297	1.285214
CECR7	0.632974	-1.21445	0.224577	0.302586	1.324109
TRG-AS1	0.740497	-1.2021	0.229325	0.453719	1.208535
HLA-F-AS	0.714497	-1.19493	0.232115	0.411649	1.240149
LINC00893	0.586413	-1.1929	0.232907	0.243982	1.409449
FLJ20021	1.169531	1.183389	0.236655	0.902337	1.515845
FAM66D	0.617267	-1.17981	0.238076	0.276946	1.375785
HOXB-AS:	1.185576	1.172028	0.241186	0.891863	1.576016
ARHGAP5-	0.682641	-1.15685	0.247335	0.3575	1.303493
DGCR5	0.664555	-1.14684	0.251447	0.330548	1.336063
LINC00565	0.638193	-1.13694	0.255562	0.294246	1.384182
HOXD-AS.	1.169589	1.133256	0.257107	0.89201	1.533546
LINC00337	1.226007	1.12594	0.260191	0.859904	1.747977
SDCBP2-A	0.451524	-1.11412	0.265226	0.11148	1.828794
SOX9-AS1	0.725953	-1.09459	0.273695	0.409122	1.288142
LINC01281	0.742636	-1.09298	0.274402	0.43556	1.266205
RASSF8-A.	0.624459	-1.08577	0.277581	0.266907	1.460993
N4BP2L2-	0.53088	-1.07252	0.283488	0.166895	1.688691
PSMB8-AS	0.768743	-1.06716	0.285898	0.474248	1.246111
FAM13A-A	0.711377	-1.06218	0.288153	0.379481	1.333549
DHRS4-AS	0.711327	-1.0572	0.290422	0.378283	1.337587
LINC00909	0.79496	-1.01644	0.309418	0.510724	1.237385
TRPM2-AS	1.215765	1.013482	0.31083	0.83322	1.773943
RARA-AS1	1.165827	0.996054	0.319224	0.862018	1.576709
ZMIZ1-AS	0.76667	-0.98677	0.323754	0.452287	1.299581
SBF2-AS1	0.778855	-0.98136	0.326415	0.472797	1.283035
PARD3-AS	1.256719	0.978486	0.327834	0.795167	1.986179
MIR100HG	0.675813	-0.9719	0.331099	0.306654	1.489372
MIR210HG	1.170946	0.96867	0.33271	0.850868	1.611431
ZBTB11-A	0.814627	-0.96377	0.335159	0.536885	1.23605
SERTAD4-	0.815695	-0.95472	0.339719	0.536909	1.239236
SPATA13	0.779541	-0.95175	0.341222	0.466768	1.301898
LINC00886	1.152785	0.937736	0.34838	0.856423	1.551702
FOXD3-AS	0.749129	-0.93645	0.349043	0.409267	1.371219
DLGAP1-A	0.694645	-0.93283	0.350907	0.323067	1.493598
TMPO-AS	0.766167	-0.93189	0.351392	0.437554	1.341576
KANSL1-A	1.151835	0.93087	0.351921	0.855326	1.551132

TIPARP-AS	0.768641	-0.9263	0.354288	0.44048	1.341284
PP7080	1.177601	0.920709	0.357203	0.831497	1.667769
TFAP2A-A	0.71493	-0.91043	0.362595	0.347156	1.472322
NR2F1-AS	1.22008	0.896469	0.370002	0.789798	1.884781
AC005498	0.758026	-0.88526	0.376014	0.410493	1.399788
TPT1-AS1	0.788123	-0.86775	0.385534	0.460291	1.349446
LINC-PINT	0.47006	-0.86512	0.386974	0.084997	2.599589
BDNF-AS	1.170591	0.860607	0.389455	0.817745	1.675687
HOXA-AS	1.172112	0.853789	0.393222	0.814035	1.687702
PITPNA-A	1.204045	0.850684	0.394945	0.784952	1.846894
FAM83C-A	1.202341	0.850569	0.395009	0.786357	1.838381
HOXC-AS	0.793798	-0.83804	0.402008	0.462548	1.36227
TMEM161	1.16244	0.831348	0.405777	0.815182	1.657628
NOP14-AS	1.173285	0.827763	0.407805	0.803657	1.712916
LINC00957	1.12357	0.825636	0.40901	0.852082	1.481559
TYMSOS	0.841563	-0.82432	0.409756	0.558428	1.268252
THUMPD3	0.817929	-0.81546	0.414807	0.504577	1.325879
PAXBP1-A	0.701766	-0.8146	0.415304	0.299311	1.645366
ALDH1L1-	0.825298	-0.80544	0.420565	0.517239	1.316832
NADK2-AS	0.500997	-0.7962	0.425914	0.091398	2.746203
RMST	0.497901	-0.7877	0.430872	0.087816	2.823025
PRKCQ-AS	1.132941	0.779842	0.435484	0.827883	1.550406
PCED1B-A	0.815205	-0.77585	0.437836	0.486529	1.365919
SNHG4	0.832789	-0.77074	0.440861	0.522949	1.326207
RBPMS-AS	1.158909	0.766547	0.443351	0.794846	1.689725
PAXIP1-AS	0.835543	-0.76312	0.445395	0.526691	1.325507
RHPN1-AS	1.164268	0.754546	0.450522	0.784292	1.728338
MIR31HG	1.139486	0.753063	0.451412	0.811176	1.600674
LINC00944	0.839949	-0.74928	0.453689	0.532248	1.325537
AP001063.	0.793653	-0.74799	0.454469	0.433142	1.454223
GRIK1-AS	0.748864	-0.7479	0.454523	0.350961	1.597892
ARHGEF7-	1.117459	0.744408	0.45663	0.834146	1.496997
UBL7-AS1	0.829759	-0.73954	0.459582	0.506002	1.360664
RAD51-AS	1.162042	0.737832	0.460617	0.779772	1.731712
STAU2-AS	0.772125	-0.72967	0.465594	0.385481	1.546577
LINC00892	0.847741	-0.72874	0.466158	0.543658	1.321905
DLG5-AS1	0.832903	-0.72483	0.468558	0.508017	1.365559
ATP1A1-A	0.718578	-0.7105	0.477395	0.288766	1.78814
LPP-AS2	0.871164	-0.70721	0.479439	0.594416	1.276758
LINC01354	1.123268	0.703022	0.482042	0.812344	1.553198
FGF14-AS	0.843227	-0.69767	0.485383	0.522277	1.361408
LINC01018	1.153081	0.696235	0.486281	0.772181	1.721869
MAP3K14-	0.857984	-0.69541	0.486797	0.55718	1.321183
SEC24B-A	0.841141	-0.69527	0.486889	0.516502	1.369825
SSSCA1-A	1.13157	0.691617	0.489178	0.797176	1.606232
LINC01085	0.708103	-0.68728	0.491906	0.264611	1.8949
TAPT1-AS	1.111403	0.680059	0.496467	0.819723	1.506871
LINC00624	0.757745	-0.67043	0.502586	0.336755	1.70503
PGM5-AS	1.103893	0.669112	0.503424	0.82639	1.474584
IGF2-AS	0.084781	-0.66793	0.504181	6.07E-05	118.3327
NFYC-AS1	0.839277	-0.64183	0.52098	0.491514	1.433094
TMEM191	0.869598	-0.63564	0.525014	0.565208	1.337915
LINC00174	0.736544	-0.62973	0.528873	0.284359	1.907787
ADIRF-AS	0.849973	-0.62969	0.528898	0.512474	1.409737
RBM26-AS	1.124181	0.606908	0.543912	0.770309	1.640618
PHEX-AS1	0.83109	-0.59411	0.552439	0.451406	1.530131
ST7-OT4	4.40E-11	-0.5935	0.552845	2.77E-45	6.99E+23
LINC01144	1.115495	0.589287	0.555669	0.775516	1.604518

FAM201A	1.115673	0.585796	0.558012	0.773549	1.609111
NCBP2-AS	0.883107	-0.58547	0.558231	0.582483	1.338885
LINC0096	0.227185	-0.58355	0.559526	0.001565	32.96984
LINC0085	1.105405	0.580864	0.561332	0.788261	1.550147
AC024560	0.879173	-0.5649	0.572142	0.562389	1.374398
USP46-AS	0.887637	-0.5604	0.575207	0.585049	1.346725
PWAR6	0.817009	-0.55796	0.576874	0.4017	1.661698
MAN2C1	0.871772	-0.55278	0.580416	0.535909	1.418126
TP73-AS1	1.116078	0.536247	0.591788	0.747089	1.667312
ACTA2-AS	0.853154	-0.53582	0.59208	0.477239	1.525171
GLIDR	0.872651	-0.52787	0.59759	0.52624	1.447097
PTPRG-AS	0.870707	-0.52593	0.598937	0.519751	1.458641
LINC0032	1.102947	0.521828	0.60179	0.763345	1.593632
LINC0120	0.869755	-0.51965	0.603311	0.51383	1.472228
RAP2C-AS	0.859903	-0.5149	0.606621	0.484097	1.527447
WDR86-A	1.074575	0.5052	0.613418	0.812927	1.420436
APTR	0.913145	-0.49499	0.62061	0.637222	1.308544
FGF13-AS	0.899377	-0.49101	0.623419	0.588968	1.373384
SNHG17	0.895242	-0.48974	0.624321	0.574914	1.394049
DLEU2	0.887213	-0.48919	0.624708	0.549285	1.433037
RAPGEF4-	0.486194	-0.4837	0.628599	0.026167	9.03371
TPTEP1	1.105806	0.482132	0.629712	0.734708	1.664345
AC144652	0.899209	-0.47474	0.634972	0.579929	1.394269
ENTPD3-A	1.104065	0.472433	0.636618	0.732191	1.66481
LINC0089	0.791234	-0.46875	0.639246	0.297231	2.106278
FAM222A-	1.076442	0.459649	0.645768	0.786286	1.473672
ITGA9-AS	0.891422	-0.45623	0.648222	0.544056	1.460573
FGF10-AS	1.083983	0.452331	0.651031	0.76431	1.537358
NPTN-IT1	0.676362	-0.45023	0.652547	0.123283	3.710695
ERVH48-1	0.223556	-0.44316	0.657647	0.000296	168.5896
CKMT2-AS	0.896842	-0.4315	0.666104	0.546944	1.470583
ST7-AS2	3.11E-25	-0.4299	0.667272	#######	1.68E+87
ASH1L-AS	1.084646	0.426417	0.669804	0.746608	1.575735
XIST	0.635375	-0.42421	0.671416	0.078157	5.165277
LDLRAD4-	0.7639	-0.41685	0.676791	0.215321	2.710103
C6orf99	1.115149	0.410201	0.681658	0.662481	1.877124
C14orf132	1.074646	0.400983	0.688433	0.755863	1.527875
ZFAS1	1.081514	0.397977	0.690647	0.73524	1.590873
KCNQ1-A	0.900743	-0.39721	0.691213	0.537757	1.508744
OGFRP1	0.893999	-0.39375	0.693767	0.511808	1.561589
FTX	0.667157	-0.38746	0.698417	0.086116	5.168614
PWAR5	0.609506	-0.37355	0.70874	0.045372	8.18785
TTLL7-IT1	0.78427	-0.37259	0.709455	0.218429	2.815923
MAPKAPK	0.920387	-0.36626	0.71417	0.590428	1.434743
HOTAIR	1.074152	0.355533	0.72219	0.724116	1.593394
DICER1-A	0.921692	-0.35223	0.724665	0.585501	1.450924
ZNF582-A	1.074446	0.338783	0.734773	0.709207	1.627781
EPN2-AS1	0.606558	-0.33141	0.740332	0.031535	11.6669
IQCH-AS1	0.933928	-0.31415	0.753406	0.609677	1.430628
LINC0066	0.943267	-0.31006	0.756518	0.652065	1.364514
DOCK4-A	0.527874	-0.30716	0.758721	0.008954	31.1201
MNX1-AS	1.06842	0.305615	0.759897	0.6989	1.633312
TMEM75	0.463717	-0.30558	0.759925	0.003355	64.10303
COX10-AS	0.925089	-0.30325	0.7617	0.559268	1.530196
C17orf100	0.924909	-0.29179	0.77045	0.547498	1.562485
VPS9D1-A	0.940709	-0.27635	0.782278	0.609808	1.451167
LINC0126	0.946462	-0.27539	0.783013	0.639781	1.400152
MEG3	0.839582	-0.26814	0.78859	0.233887	3.013836

ELOVL2-A	0.9525	-0.26009	0.794797	0.660078	1.374469
CTBP1-AS	1.058413	0.257063	0.79713	0.686553	1.631684
LINC0011!	0.941046	-0.25705	0.79714	0.592105	1.495626
AC067956	1.046	0.25305	0.80023	0.738333	1.481872
AFAP1-AS	1.046234	0.248245	0.803945	0.732241	1.494869
NEBL-AS1	1.05148	0.241904	0.808854	0.700105	1.579207
LINC0121!	0.946871	-0.2373	0.812421	0.60321	1.486321
SHANK3	1.049053	0.228058	0.819601	0.695122	1.583193
MATN1-A	1.041264	0.227102	0.820345	0.73452	1.476109
PTOV1-AS	0.94943	-0.22568	0.821452	0.604968	1.490024
OIP5-AS1	1.047807	0.218045	0.827394	0.688614	1.594361
RPARP-AS	0.937028	-0.2154	0.829452	0.518473	1.693473
RNASEH1-	0.959807	-0.21331	0.831087	0.658387	1.399222
AC006538	0.95348	-0.20572	0.837006	0.605631	1.501118
HCG9	0.943723	-0.20479	0.837734	0.542119	1.642837
KCNJ2-AS	1.038352	0.204499	0.837964	0.723927	1.489342
MED14OS	1.052832	0.188041	0.850845	0.615612	1.800575
TTLL11-IT	0.958996	-0.17611	0.860211	0.601793	1.528222
WEE2-AS1	0.963135	-0.17029	0.864779	0.625083	1.484009
RNF139-A	0.963615	-0.16205	0.87127	0.615484	1.508657
WFDC21P	1.031787	0.159971	0.872904	0.703208	1.513896
GAS5-AS1	1.034169	0.157347	0.874971	0.68051	1.571623
GAS5	1.032304	0.155289	0.876594	0.691095	1.541974
LINC0118(	1.021578	0.146648	0.88341	0.767989	1.358902
SMCR5	0.209398	-0.14061	0.888178	7.18E-11	6.11E+08
RAMP2-AS	0.976205	-0.13842	0.889905	0.694152	1.372866
LINC0065	0.964579	-0.138	0.890238	0.577964	1.609812
KMT2E-AS	1.02433	0.12549	0.900135	0.703694	1.491064
PART1	0.978557	-0.10741	0.914463	0.658884	1.453328
FOXD2-AS	1.021065	0.104394	0.916857	0.690369	1.510169
RBMS3-AS	1.021844	0.103798	0.91733	0.679482	1.536709
LINC0109	0.981886	-0.09417	0.924972	0.671164	1.436458
LINC0112	0.982024	-0.08994	0.928335	0.661375	1.458131
LINC0086	0.972986	-0.08811	0.92979	0.529108	1.789241
LINC0064	0.978772	-0.08591	0.931536	0.599917	1.596878
KRTAP5-A	0.981271	-0.08585	0.931584	0.63728	1.51094
CIRBP-AS	0.983357	-0.08538	0.931959	0.668946	1.445544
SNHG10	0.98259	-0.07213	0.942501	0.609678	1.583595
LIPE-AS1	1.013972	0.071213	0.943229	0.692104	1.485529
MAGI2-AS	0.983892	-0.06832	0.94553	0.617494	1.567697
LINC0017	1.014066	0.06573	0.947593	0.668633	1.537958
USP27X-A	1.013718	0.061072	0.951302	0.65467	1.569683
MIR205HC	0.98574	-0.05765	0.954029	0.604899	1.606355
LINC0099	1.008905	0.040317	0.967841	0.655659	1.552467
CRNDE	1.009084	0.039731	0.968307	0.645944	1.576376
ZFHX4-AS	0.989864	-0.03197	0.974494	0.530082	1.84845
SENCR	1.006951	0.03154	0.974839	0.65473	1.548655
AC017002	0.995216	-0.03085	0.97539	0.73383	1.349705
LINC0064	0.994166	-0.02704	0.978428	0.650509	1.519372
LINC0092	1.004928	0.026208	0.979091	0.695788	1.451419
LINC0091	0.996409	-0.01742	0.986098	0.664825	1.493373
ST8SIA6-A	1.002387	0.011002	0.991222	0.655544	1.532742
ITGB2-AS	0.99833	-0.00808	0.99355	0.665663	1.497247
LINC0062	0.99586	-0.00652	0.994801	0.285944	3.468292
