# Supplementary figures and images for: Development of a Prognostic Model Based on the Identification of EMT-Related lncRNAs in Triple-Negative Breast Cancer
Source: J Oncol. 2021 Nov 27;2021:9219961. doi: 10.1155/2021/9219961 (PMC8643262; doi:10.1155/2021/9219961)

A

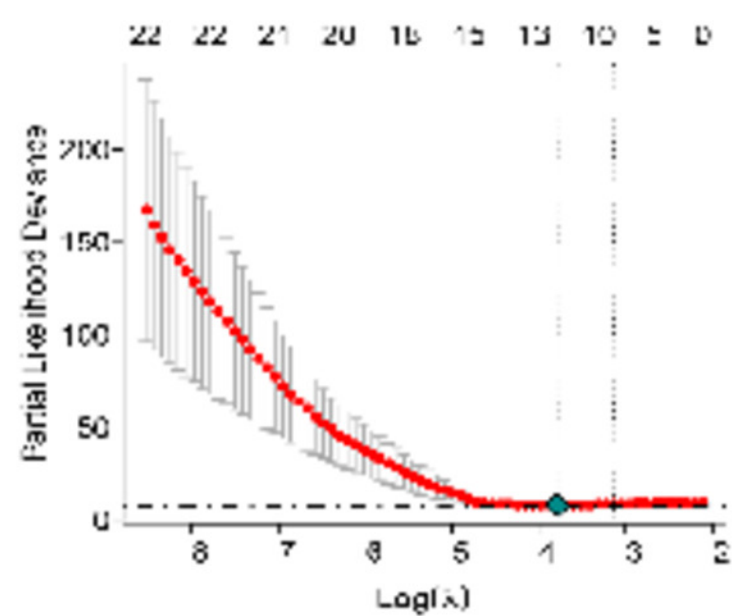

B

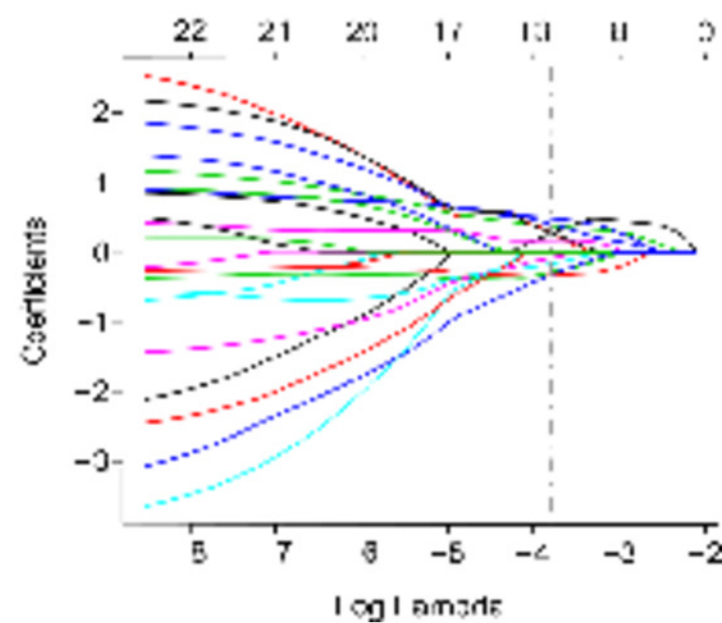

A

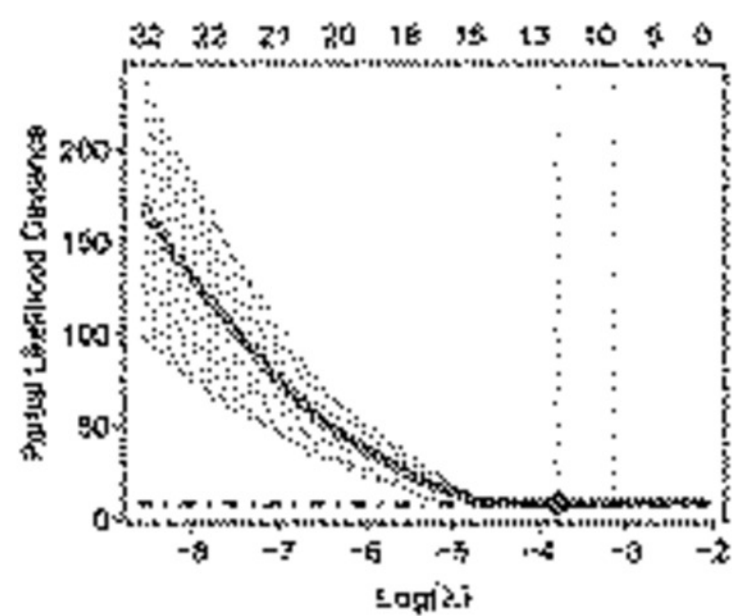

B

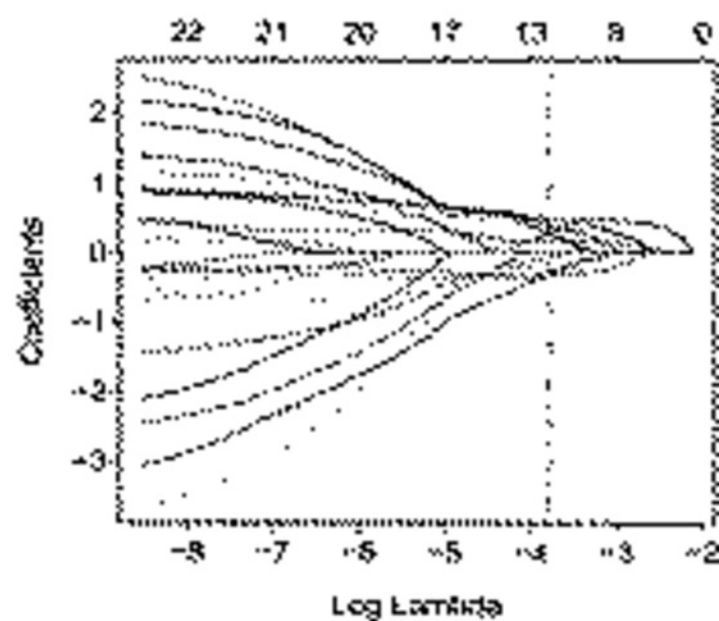

Supplement: Supplementary Materials — Supplementary Figure 1: prognostic lncRNAs were screened out from TNBC data. (A) Cox univariate regression analysis. (B) Lasso regression analysis. Supplementary Figure 2: CeRNA network. Supplementary Table 1: primers used in qRT-PCR. Supplementary Table 2: a total of 1033 lncRNAs highly associated with EMT. Supplementary Table 3: a total of 285 prognostic lncRNAs screened by Cox regression analysis. [file 9219961.f1.zip › 9219961.f1/Fig S1.pdf]

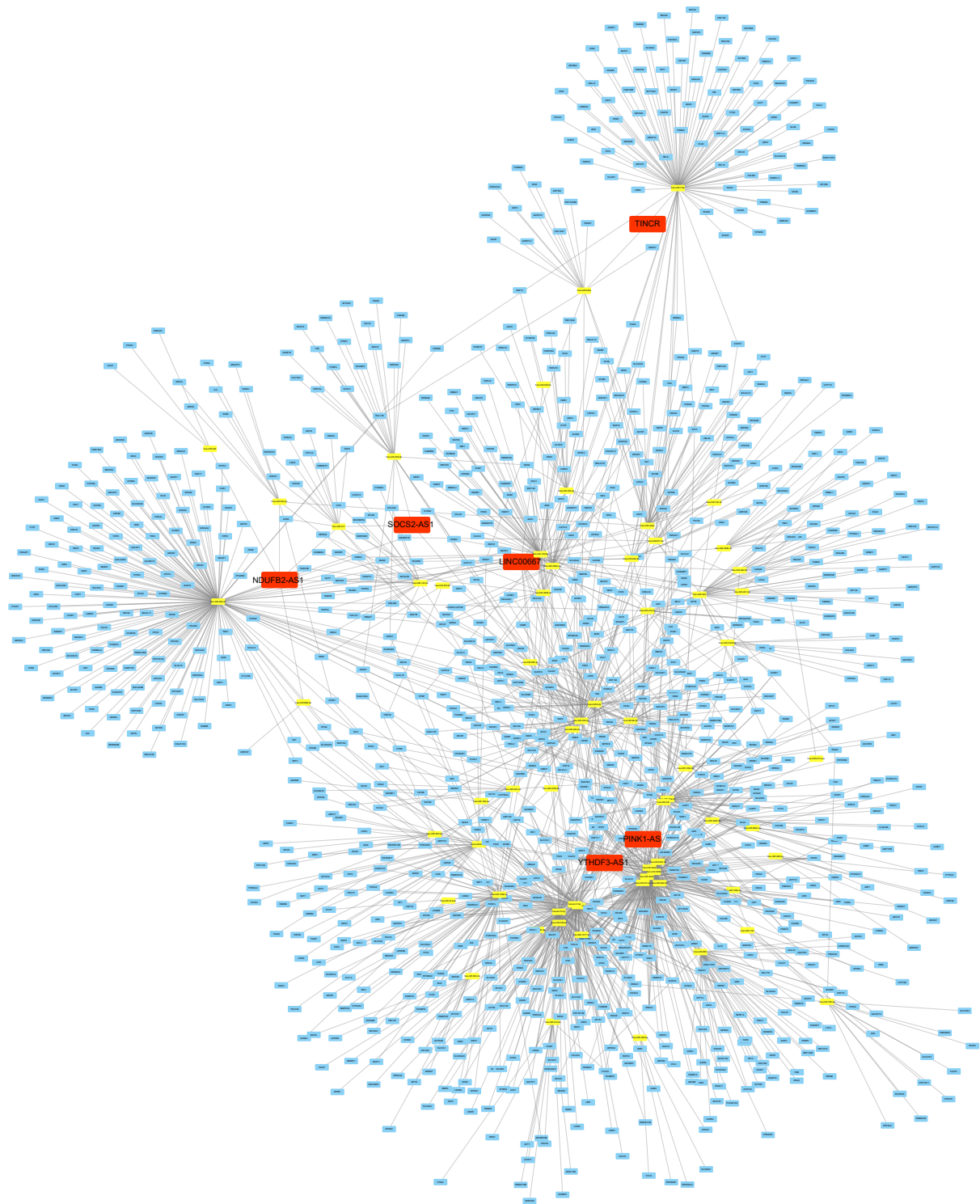

Supplement: Supplementary Materials — Supplementary Figure 1: prognostic lncRNAs were screened out from TNBC data. (A) Cox univariate regression analysis. (B) Lasso regression analysis. Supplementary Figure 2: CeRNA network. Supplementary Table 1: primers used in qRT-PCR. Supplementary Table 2: a total of 1033 lncRNAs highly associated with EMT. Supplementary Table 3: a total of 285 prognostic lncRNAs screened by Cox regression analysis. [file 9219961.f1.zip › 9219961.f1/Fig S2 (1).pdf]
